# Integrated screening identifies GPR31 as a key driver and druggable target for metabolic dysfunction–associated steatohepatitis

**DOI:** 10.1172/JCI173193

**Published:** 2025-09-02

**Authors:** Xiao-Jing Zhang, Jiajun Fu, Xu Cheng, Hong Shen, Hailong Yang, Kun Wang, Wei Li, Han Tian, Tian Tian, Junjie Zhou, Song Tian, Zhouxiang Wang, Juan Wan, Lan Bai, Hongfei Duan, Xin Zhang, Ruifeng Tian, Haibo Xu, Rufang Liao, Toujun Zou, Jing Shi, Weiyi Qu, Liang Fang, Jingjing Cai, Peng Zhang, Zhi-Gang She, Jingwei Jiang, Yufeng Hu, Yibin Wang, Hongliang Li

**Affiliations:** 1 State Key Laboratory of New Targets Discovery and Drug Development for Major Diseases, Gannan Innovation and Translational Medicine Research Institute, School of Pharmacy, First Affiliated Hospital, Gannan Medical University, Ganzhou, China.; 2 Department of Cardiology, Renmin Hospital of Wuhan University, Wuhan, China.; 3 State Key Laboratory of Quality Research in Chinese Medicine, Institute of Chinese Medical Sciences, University of Macau, Macau, China.; 4 School of Basic Medical Sciences, Wuhan University, Wuhan, China.; 5 Department of Cardiology and; 6 Department of Radiology, Zhongnan Hospital of Wuhan University, Wuhan, China.; 7 Department of Gastroenterology, Huanggang Central hospital of Yangtze University, Huanggang Institute of Translational Medicine, Huanggang, China.; 8 Department of Cardiology, The Third Xiangya Hospital, Central South University, Changsha, China.; 9 Jiangsu Key Laboratory of Drug Screening, China Pharmaceutical University, Nanjing, China.; 10 Signature Research Program in Cardiovascular and Metabolic Diseases, Duke-NUS Medical School, Singapore.; 11 Medical Science Research Center, Zhongnan Hospital of Wuhan University, Wuhan, China.

**Keywords:** Hepatology, Metabolism, G protein-coupled receptors

## Abstract

Metabolic dysfunction–associated steatohepatitis (MASH) is a globally prevalent but intractable disease lacking effective pharmacotherapies. Here, we performed an integrated multilayered screening for pathogenic genes and druggable targets for MASH. We identified the subclass of metabolite-sensing G protein–coupled receptors, specifically GPR31, a critical contributor to MASH occurrence, which, to our knowledge, was previously uncharacterized. Mechanistically, Gαi3 is the essential downstream effector for the pro-MASH efficiency of GPR31 via glycosylation-dependent interaction with GPR31 and extra activation of PKCδ-MAPK signaling. Hepatocyte-specific GPR31 deficiency robustly blocked hepatic lipotoxicity and fibrosis in a mouse model of diet-induced MASH, whereas expression of the GPR31 transgene aggravated MASH development. Of translational importance, we developed a small-molecule inhibitor, named G4451, that specifically inhibits the GPR31-Gαi3 interaction by targeting the GPR31 conformational transition. Encouragingly, oral administration of G4451 effectively blocked MASH progression in preclinical models in both rodents and nonhuman primates. Collectively, the present study provides proof of concept that interference with GPR31 constitutes an attractive therapeutic strategy for MASH.

## Abstract 2

GPR31 is identified as a key driver and potential therapeutic target for MASH via its glycosilation and G protein binding.

## Graphical abstract

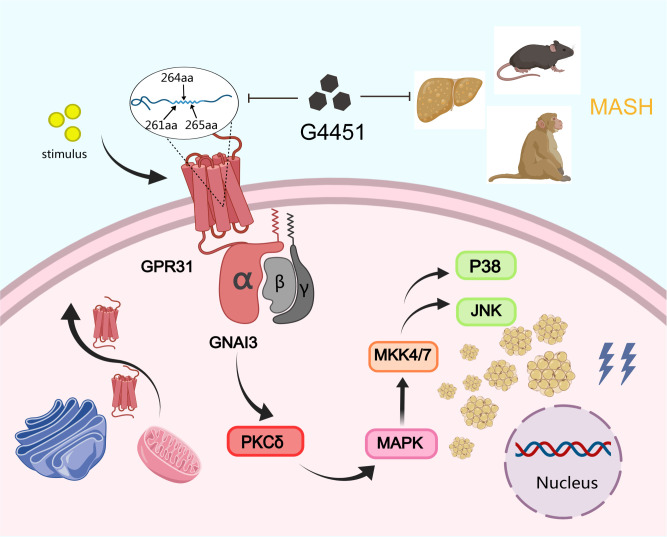

## Introduction

Metabolic dysfunction–associated steatotic liver disease (MASLD) has become the most prevalent liver disease, affecting more than 30% of adults worldwide (1). The advanced form of MASLD, metabolic dysfunction–associated steatohepatitis (MASH), puts patients at higher risk of end-stage liver diseases, such as cirrhosis, hepatocellular carcinoma, and liver failure (2). MASH is currently a leading indication for liver transplantation in both developed and developing countries (3). Furthermore, MASH-related impairment of systemic metabolic homeostasis is usually accompanied by type 2 diabetes, obesity, hyperlipidemia, cardiovascular diseases, and cancer (4). Thus, there is a huge clinical demand for anti-MASH pharmacotherapies, which has stimulated intense efforts in basic research and drug development. However, only one anti-MASH drug, resmetirom, has been approved by the US Food and Drug Administration (FDA), and comprehensive evaluation of adverse events, efficacy, cost-effectiveness, social acceptance, and accessibility remains imperative. Unfortunately, several highly promising drugs for MASH targeted to well-studied targets have failed in late-stage clinical trials over the last several years (5). Thus, there is an urgent unmet medical need for safe and efficacious pharmacotherapies for MASH.

G protein–coupled receptors (GPCRs) are among the best druggable targets, having characteristics such as easy access on the cell membrane, high specificity and sensitivity to external stimuli, and profound impact on intracellular signal transduction (6, 7). Avenues for MASH therapy based on GPCR-targeted drug discovery have recently emerged, particularly the strategies targeting a subfamily of GPCRs termed metabolite-sensing GPCRs (8). In our present study, we employed an integrated transcriptomics analysis and revealed a close correlation of the metabolite-sensing GPCR subfamily with MASH progression. This type of GPCR can sense microenvironmental metabolites to transmit signals for proper immune and metabolic functions and is therefore closely correlated with the progression of metabolic diseases (8). For instance, GPR43 binds to short-chain fatty acids to enhance insulin secretion in cells (9); GPR75 can recognize 20-HETE and regulates obesity (10); and GPR40 and GPR120 can improve glycemic control, increase insulin sensitivity, and reduce inflammation via incretin release (11–13). With advances in screening technology and the discovery of mediators, the number of identified metabolite-sensing GPCRs continues to rise (14). However, the specific metabolite-sensing GPCR contributing to MASH progression remains largely unknown.

In this study, we performed an unbiased and multilayered screening based on multiomics analysis and experimental validation. We found that GPR31 exhibited the most robust effect in promoting hepatocyte lipotoxicity and MASH by direct interaction with Gαi3 and downstream PKCδ-dependent MAPK signaling. Remarkably, the pro-MASH activity of GPR31 and its interaction with Gαi3 are mediated by a targeted glycosylation at its N-terminus, asparagine (Asn, N) 5). Of clinical importance, we have developed a small molecule that specifically blocks the GPR31-Gαi3 interaction and have demonstrated both in mice and monkeys that it can effectively reverse MASH.

## Results

### Integrated screening identifies GPR31 as a key driver for MASH.

**Figure 1:**
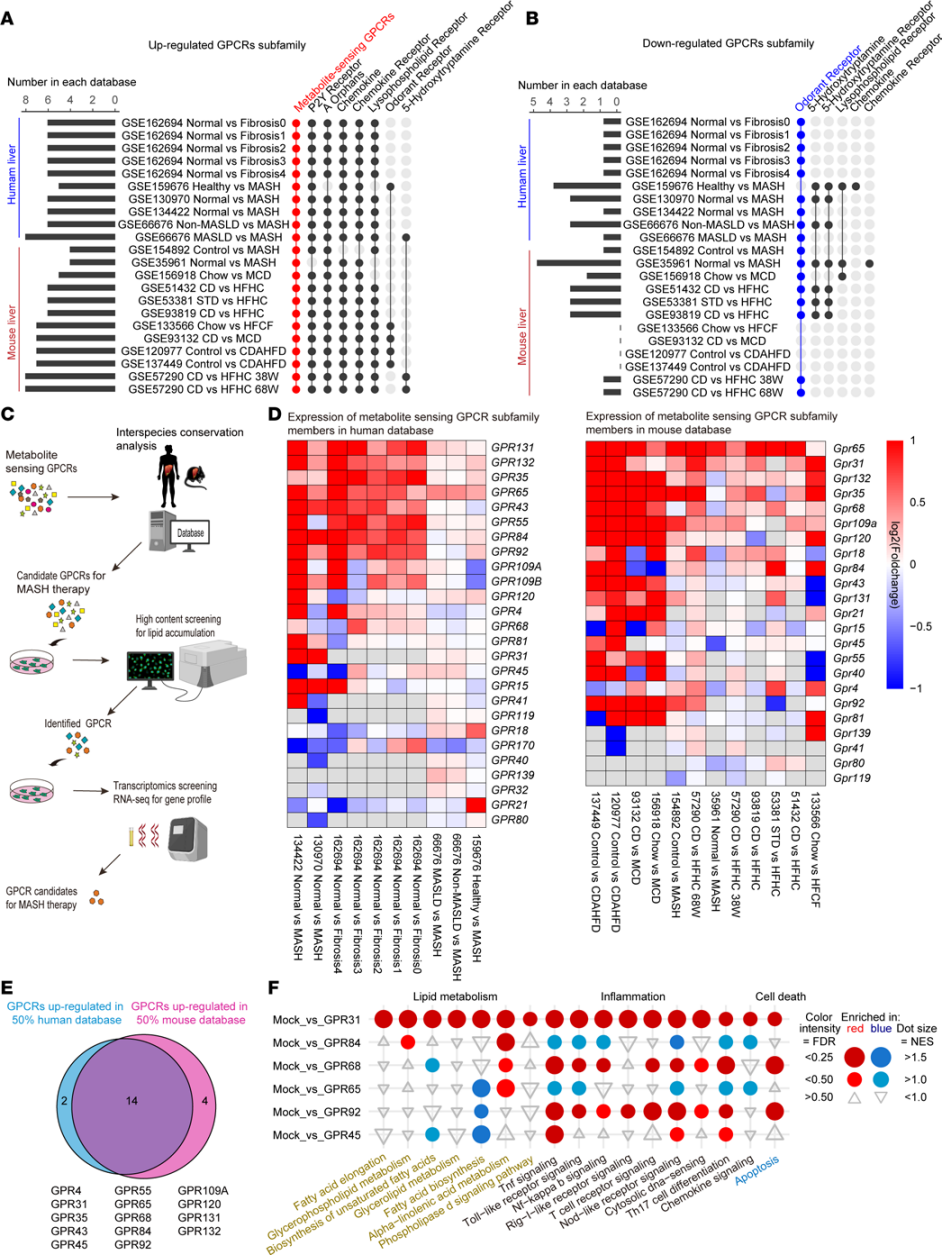
GPR31 was identified as a metabolite-sensing GPCR facilitating MASH development. (**A**) GPCR subfamilies that were upregulated in MASLD based on human and mouse liver transcriptomics. (**B**) GPCR subfamilies that were downregulated in MASLD based on human and mouse liver transcriptomics. (**C**) Screening strategy to identify key metabolite-sensing GPCR driving MASH progression. (**D**) Expression of metabolite-sensing GPCR subfamily members in human and mouse database. (**E**) Conservatively upregulated members in both human and mouse fatty livers in metabolite-sensing GPCR subfamily. (**F**) Differentially expressing genes of hepatocytes with overexpression of tested GPCRs after PA stimulation.

To identify candidate therapeutic targets for MASH, we first analyzed the expression landscape of druggable targets using the Therapeutic Target Database (http://db.idrblab.net/ttd/) (15) (Supplemental Figure 1A; supplemental material available online with this article; https://doi.org/10.1172/JCI173193DS1). The plasma membrane proteins were the most significantly enriched druggable targets (Supplemental Figure 1B), of which GPCR 1 family members were the most abundantly represented (Supplemental Figure 1C). We then collected and analyzed public transcriptomic data available in the NCBI’s Gene Expression Omnibus (GEO), including non-MASH versus MASH liver sample data from clinical samples and mouse models. Notably, among all subfamilies that positively or negatively correlated with MASH status, the metabolite-sensing GPCRs showed the highest degree of enrichment (Figure 1, A and B, and Supplemental Figure 1D). By gene set enrichment analysis (GSEA), we further verified the enrichment of metabolite-sensing GPCRs in liver samples from patients with MASH and in mouse MASH models (Supplemental Figure 1E). The activity extent of the metabolite-sensing GPCR subfamily was also found to be highly correlated with MASH-related genes (Supplemental Figure 1F), suggesting a close participation and contribution of this GPCR subfamily in MASH progression.

To explore the specific metabolite-sensing GPCR(s) that drive MASH and can also serve as potential anti-MASH therapeutic targets, we further analyzed the RNA-Seq database for individual subfamily members. Fourteen members of the metabolite-sensing GPCR subfamily were found to exhibit conserved upregulation in both human and mouse fatty liver samples (Figure 1, C–E). Since hepatocytes are the primary cell type in the liver that sense and respond to extracellular microenvironments, we systemically analyzed the participation and contribution of those 14 GPCR members to steatosis development in hepatocytes by high-content functional screening and transcriptomics assay (Figure 1F and Supplemental Figure 1, G–I). First, we expressed each Flag-tagged GPCR in hepatocytes and characterized the changes in cellular lipid accumulation in response to hepatocyte lipotoxicity induced by palmitic acid/oleic acid (PA/OA). Six of the 14 GPCR members showed significant inhibitory (GPR45) or exacerbating (GPR31, GPR65, GPR68, GPR84, and GPR92) effects on lipid accumulation in hepatocytes (Supplemental Figure 1, G and H). In addition, we performed RNA-Seq assays in hepatocytes after 3 hours of PA stimulation to further explore the influences on gene profile related to lipid metabolic disorder, inflammatory response, and cell injury from these 6 identified GPCRs. Notably, GPR31 overexpression in hepatocytes resulted in the most extensive activation of steatosis-related pathways (Figure 1F) and was also associated with the most consistent pattern of differentially expressed genes (DEGs) among all candidate GPCRs (Supplemental Figure 1I). The correlation of GPR31 expression with MASH-related molecular events were then analyzed using gene set variation analysis. The expression level of GPR31 was significantly correlated with genes and pathways of metabolic disorders, inflammatory response, fibrosis, and cell damage (Supplemental Figure 1J).

### GPR31 shows pro-MASH capacity by promoting hepatocyte lipotoxicity in vitro.

**Figure 2:**
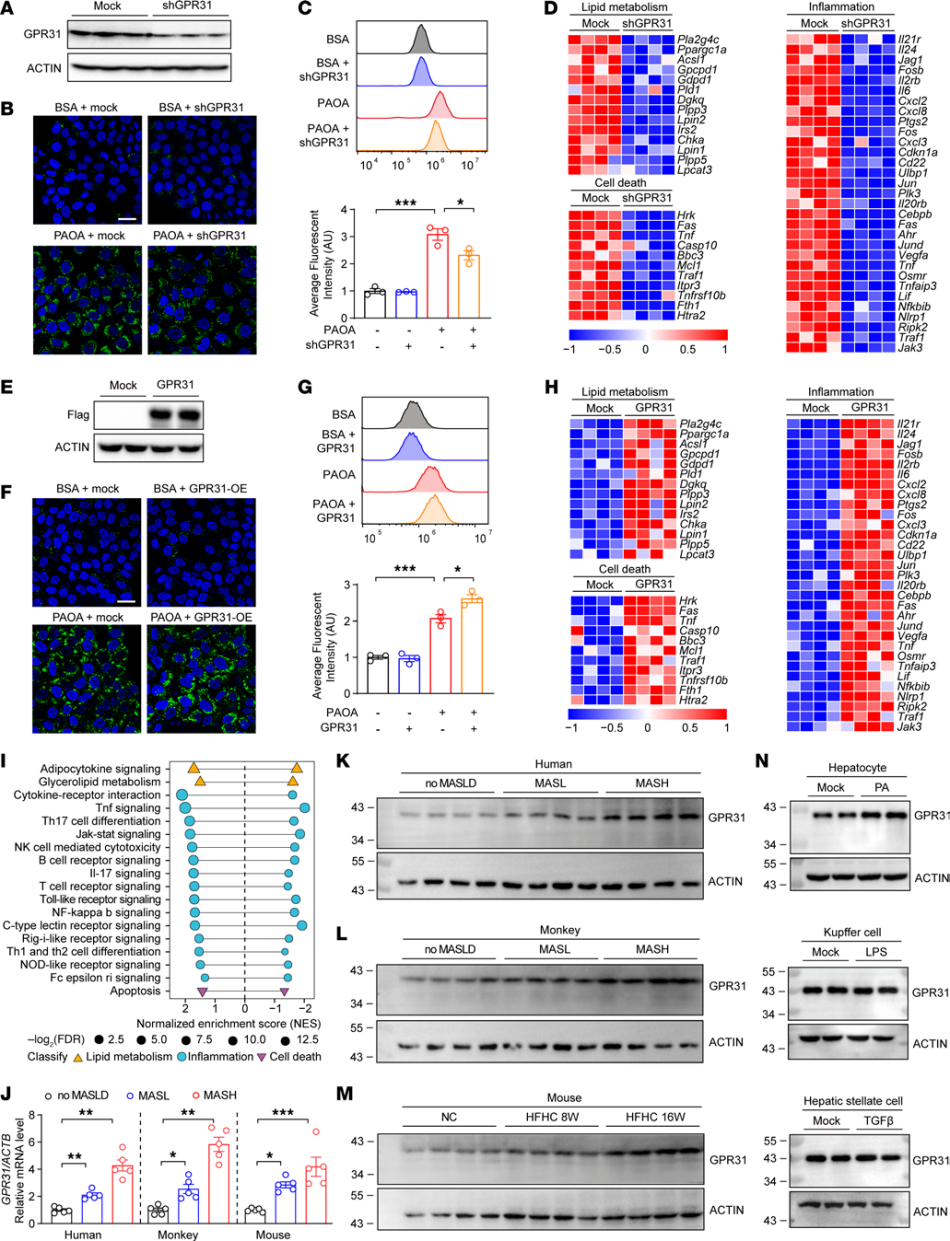
GPR31 shows pro-MASH capacity by promoting hepatocyte lipotoxicity in vitro. (**A**) The knockdown effects of GPR31 validated by Western blotting. *n* = 3. (**B**) BODIPY staining of lipid droplets in control and GPR31-deficient hepatocytes. Scale bars: 30 μm. *n* = 3. (**C**) BODIPY staining for quantification by flow cytometry in control and GPR31-deficient hepatocytes. *n* = 3. (**D**) RNA-Seq–based heatmap analysis of GPR31-deficient hepatocytes. *n* = 4. (**E**) The overexpression of GPR31-Flag validated by Western blotting. *n* = 3. (**F**) BODIPY staining of lipid droplets in control and GPR31-overexpressed hepatocytes. Scale bars: 30 μm. *n* = 3. (**G**) BODIPY staining for quantification by flow cytometry in control and GPR31-overexpressed hepatocytes. *n* = 3. (**H**) RNA-Seq–based heatmap analysis of GPR31-overexpressed hepatocytes. *n* = 4. (**I**) Pathway analysis of GPR31-overexpressed and GPR31-deficient mouse hepatocytes. *n* = 4. (**J**) mRNA levels of GPR31 in liver tissues from humans, monkeys, or mice at the stages of normal, MASL, and MASH. *n* = 5. (**K** and **L**) Protein levels of GPR31 in human (**K**) and monkey (**L**) liver tissues at the stage of MASL or MASH versus that in normal control. *n* = 4. (**M**) Protein levels of GPR31 in liver tissues from mice fed with NC or HFHC diet for 8 weeks and 16 weeks. *n* = 4. (**N**) Mouse primary hepatocytes, Kupffer cells, and hepatic stellate cells were treated with or without palmitic acid (PA), LPS, or TGF-β for 12 hours before whole cell lysate was collected for Western blot analysis to detect GPR31 expression. *n* = 3. Data are shown as mean ± SEM. *P < 0.05; **P < 0.01; ***P < 0.001.

We inhibited GPR31 in primary hepatocytes by adenovirus-mediated shRNA (Figure 2A). The decreased GPR31 expression led to significantly ameliorated lipid deposition in hepatocytes and largely inhibited the expression of genes related to lipid synthesis, inflammatory response, and cell death (Figure 2, B–D, and Supplemental Figure 2A). In contrast to the observations made in GPR31-knockdown cells, GPR31 overexpression markedly promoted lipid accumulation upon PA/OA challenge (Figure 2, E–G, and Supplemental Figure 2B). Transcriptomic analysis revealed that GPR31-induced genes in hepatocytes were significantly enriched with functions involved in lipid metabolism and inflammation (Figure 2, H and I). In patient samples, hepatic GPR31 mRNA level was progressively induced during MASL to MASH progression relative to healthy controls (Figure 2J). Likewise, GPR31 mRNA levels were also significantly increased in liver tissues from monkeys and mice during the progression of MASH (Figure 2J). Consistent with the mRNA changes, the GPR31 protein levels were also increased in fatty livers, correlating well with the disease progression (Figure 2, K–M, and Supplemental Figure 2, C and D). Fatty acid–induced GPR31 expression was found in hepatocytes instead of Kupffer cells, endothelial cells, or hepatic stellate cells (Figure 2N and Supplemental Figure 2E). In addition, the protection of GPR31 deficiency in hepatocytes could also affect Kupffer cells and endothelial cells assayed by coculture (Supplemental Figure 2F).

### Gαi3 is the specific downstream effector for GPR31 function.

**Figure 3:**
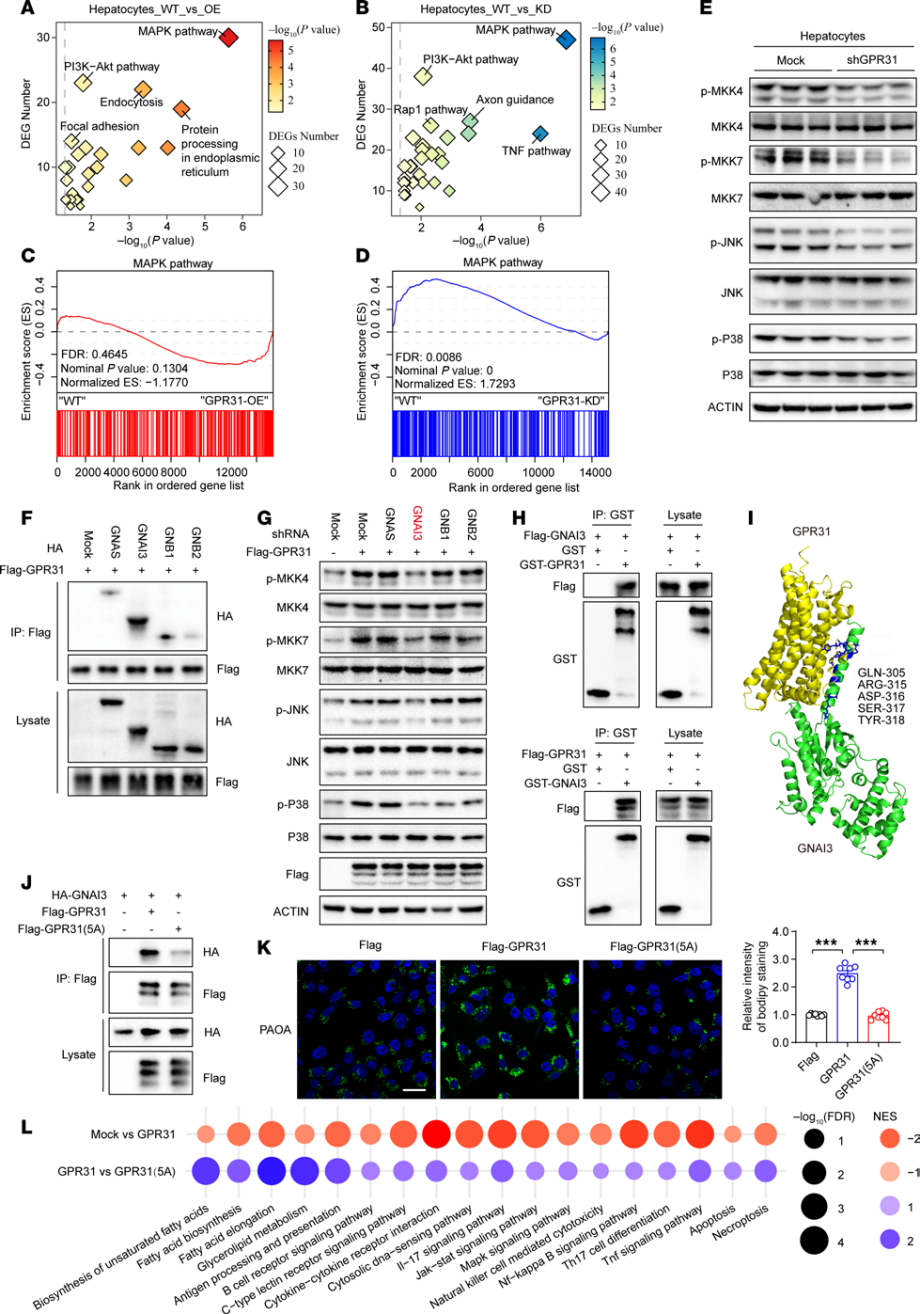
GPR31-Gαi3 interaction is essential for MASH aggravation. (**A** and **B**) The number of DEGs in enriched pathways regulated by GPR31 was analyzed integratively by comparing GPR31-overexpressed (**A**) and GPR31-deficient hepatocytes (**B**) with corresponding controls. *n* = 4. (**C** and **D**) GSEA of MAPK pathway in gain- and loss of function of GPR31 in hepatocytes and mouse liver tissues. *n* = 4. (**E**) Effect of GPR31 knockdown on the phosphorylation of key proteins in the MAPK signaling pathway in mouse primary hepatocytes. *n* = 3. (**F**) Co-IP assays were performed to examine the interaction of GPR31 and identified downstream small G proteins in HEK293T cells. *n* = 3. (**G**) Representative Western blot images showing the phosphorylation of key proteins in the MAPK signaling pathway in hepatocytes transfected with the indicated plasmids and shRNA. *n* = 3. (**H**) GST pull-down assays were performed to examine the interaction of GPR31 and GNAI3. *n* = 3. (**I**) Interacting domains of GPR31 and GNAI3 predicted by computer simulation. (**J**) Co-IP assays were performed to examine the interaction of GNAI3 and GPR31-WT or its docking mutant GPR31(5A). *n* = 3. (**K**) BODIPY staining of lipid droplets in hepatocytes with or without GNAI3 or its docking mutant GPR31(5A) overexpression. Scale bars: 30 μm. *n* = 3 with 8 images quantified. (**L**) Hepatocytes transfected with GPR31 or GPR31(5A). RNA was collected for RNA-Seq. KEGG pathway–based phenotypic characterization shown. *n* = 4. Data are shown as mean ± SEM. ***P < 0.001.

To investigate the pro-MASH mechanisms of GPR31, we performed an in-depth investigation of molecular events based on RNA-Seq datasets from cultured hepatocytes with *Gpr31* overexpression or knockdown. Our integrated transcriptome analysis found that MAPK signaling was the most significantly enriched pathway associated with GPR31 expression based on results from both GPR31 overexpressed and knockdown hepatocytes (Figure 3, A and B). GSEA results also showed the hyperactivation of MAPK signaling in *Gpr31*-overexpressing cells but a significant decrease in MAPK pathway signaling after *Gpr31* downregulation (Figure 3, C and D). The effect of GPR31 on MAPK signaling was further validated by Western blot analysis in cultured hepatocytes (Figure 3E and Supplemental Figure 3A).

We then explored the direct effector for GPR31-induced activation of MAPK signaling in hepatocytes. The canonical function of G proteins is to serve as direct effectors of GPCRs. However, different G proteins always hold specific or even converse effects in certain pathological settings. To explore the direct downstream G protein of GPR31 in the setting of MASH progression, we performed interactome assay via co-IP followed by tandem mass spectrometry (co-IP–MS/MS) (Supplemental Figure 3B). Gαs, Gαi3, Gβ1, and Gβ2 have been identified as potential G proteins binding to GPR31, and Gαi3 showed the strongest binding activity for GPR31 (Figure 3F). To further determine which G protein is the downstream mediator for GPR31 in hepatocytes, we knocked down each candidate G protein individually in *GPR31*-overexpressing hepatocytes and examined their impact on MAPK signaling activation (Figure 3G and Supplemental Figure 3C). Importantly, interruption of Gαi3 but not the other G proteins largely reversed the effect of GPR31 overexpression on the phosphorylation of MAPKs in response to PA/OA stimulation (Figure 3G and Supplemental Figure 3D). Consistent with this finding, Gαi3 overexpression led to markedly exacerbated lipid droplet formation and pro-MASH transcriptome profiles including exaggerated activation of lipid disorder pathways and inflammatory cytokine expression in hepatocytes (Supplemental Figure 3, E–J).

A direct interaction between GPR31 and Gαi3 was demonstrated by co-IP and GST-fusion protein pull-down assays (Figure 3H and Supplemental Figure 3K). An in silico–based structural docking analysis identified the binding sites for Gαi3 in GPR31 at aa 305, 315, 316, 317, and 318 (Figure 3I). Targeted mutation of these 5 binding sites to alanine (5A) greatly abrogated the direct interaction between GPR31 and Gαi3 (Figure 3J). More importantly, the mutation of those 5 binding sites in GPR31 led to an almost complete loss of MAPK activation, hepatocyte lipid deposition, and inflammatory responses (Figure 3, K and L, and Supplemental Figure 3, L and M). Thus, binding to Gαi3 is indispensable for the pro-MASH function of GPR31.

PKA and PKC have been reported to be pivotal links between Gαi3 and its downstream molecular signaling (16–20). To further explore the link between Gαi3 and MAPK activation in the setting of GPR31-mediated MASH progression, we examined the protein levels of phosphorylated and total PKA and PKC. As shown in Supplemental Figure 4, A and B, GPR31 expression significantly induced the phosphorylation of PKC but not PKA. Consistent with the pro-MASH phenotype induced by GPR31, overexpression of PKC effectively exacerbated PA-triggered hepatocyte lipid deposition, MAPK activation, and inflammation (Supplemental Figure 4, C–G). We further showed that PKCδ is required for the function of GPR31, as genetic inhibition of PKCδ reversed GPR31-triggered MAPK pathway activation, lipid accumulation, and inflammatory responses in hepatocytes (Supplemental Figure 4, H–K).

### GPR31 N-glycosylation at Asn5 is required for GPR31-Gi3 interaction and pro-MASH function.

**Figure 4:**
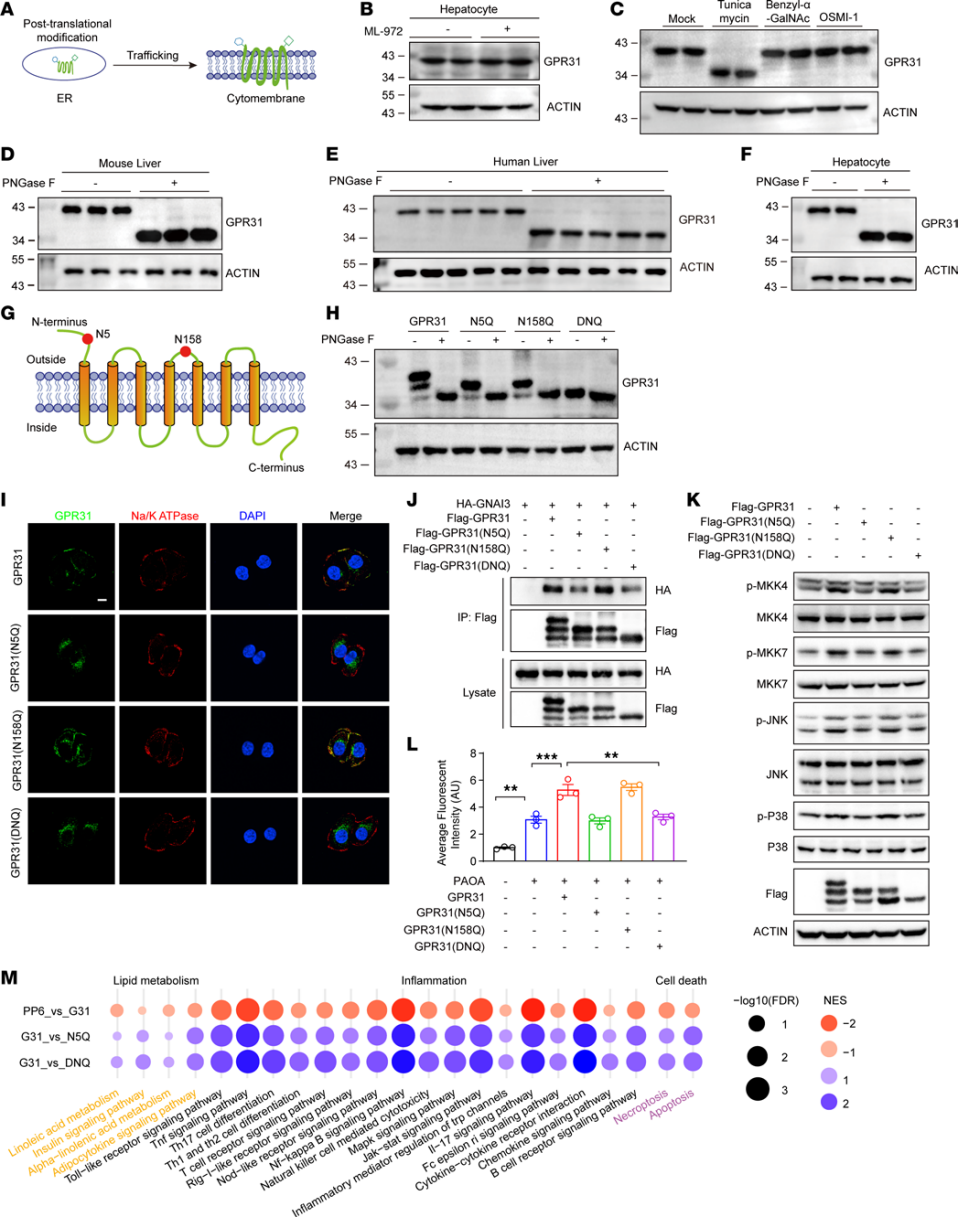
GPR31 N-glycosylation at Asn5 is required for GPR31-Gαi3 interaction and pro-MASH function. (**A**) Illustration of the trafficking of GPR31 onto cytomembrane. (**B** and **C**) The immunoblot of GPR31 in hepatocytes treated with or without sumoylation inhibitor ML-792 (**B**), or N-linked glycosylation inhibitor tunicamycin or O-linked glycosylation–specific inhibitors (benzyl-α-GalNac and OSMI-1) (**C**). *n* = 3. (**D** and **E**) GPR31 is glycosylated in mouse (**D**) and human (**E**) liver tissues evidenced by the mobility change after PNGase F treatment. *n* = 3 for **D** and *n* = 5 for **E**. (**F**) GPR31 is glycosylated in primary cultured mouse hepatocytes evidenced by the mobility change after PNGase F treatment. *n* = 3. (**G**) Illustration of potential GPR31 glycosylation site at Asn5 and Asn158 based on bioinformatic analysis of conserved glycosylation motif N-X-S/T. (**H**) Validation of Asn5 and Asn158 as the glycosylation sites in GPR31 by using PNGase F treatment. *n* = 3. (**I**) The colocalization of GPR31 and Na/K ATPase. Scale bars: 10 μm. *n* = 3. (**J**) Co-IP for the interaction of GNAI3 and GPR31 or its nonglycosylated mutants. *n* = 3. (**K**) Effect of GPR31 or its nonglycosylated mutants’ overexpression on the phosphorylation of key proteins in the MAPK signaling pathway in hepatocytes. *n* = 3. (**L**) BODIPY staining for quantification by flow cytometry in GPR31-WT and its nonglycosylated mutants’ overexpressed hepatocytes. *n* = 3. (**M**) KEGG pathway–based phenotypic characterization for hepatocytes transfected with GPR31-WT and its nonglycosylated mutants. *n* = 4. Data are shown as mean ± SEM. **P < 0.01; ***P < 0.001.

Beyond the transcriptional activation, GPCR-mediated signaling requires multistep regulation, including plasma membrane targeting from endoplasmic reticulum through Golgi (21), which can be regulated by posttranslational modifications, such as sumoylation and glycosylation (Figure 4A) (22). Indeed, we observed a mobility shift of the GPR31 band from the predicted 35 kDa to a higher molecular weight of approximately 43 kDa in immunoblot (Figure 2, K–N) (22–25). In hepatocytes treated with a sumoylation-specific inhibitor (ML-792) or O-linked glycosylation–specific inhibitors (benzyl-α-GalNac and OSMI-1), no changes in GPR31 molecular weight shifts were observed (Figure 4, B and C). In contrast, treatment with an N-linked glycosylation inhibitor, tunicamycin, resulted in a marked downward shift in the GPR31 band to the expected size of 35 kDa (Figure 4, B and C). Consistent with N-linked glycosylation of GPR31, treating samples from the liver or hepatocytes with recombinant peptide-N-glycosidase F (PNGase F) also reduced the apparent molecular weight of GPR31 to 35 kDa (Figure 4, D–F). GPR31 protein possesses 2 N-X-S/T motifs, the evolutionarily conserved N-linked glycosylation sites (22, 23), at locations of N5 and N158 (Figure 4G). When both N5 and N158 in GPR31 were mutated to glutamine (DNQ), the mutant protein also showed an apparent molecular weight of 35 kDa (Figure 4H). Single substitution of Asn with Gln at N5 (N5Q) or N158 (N158Q) lowered the apparent molecular weight in Western blot gels (Figure 4H), indicating that both N5 and N158 were glycosylation sites in GPR31.

To investigate the functional significance of N-linked glycosylation in GPR31 activity, we expressed GPR31-WT and single or double GPR31 mutants (N5Q, N158Q, and DNQ) in hepatocytes. Notably, immunofluorescence staining showed that WT and N158Q GPR31 were localized mainly in the cytomembrane of hepatocytes, colocalizing with the membrane marker Na/K ATPase, whereas the N5Q and DNQ mutations largely abolished the membrane localization of GPR31 (Figure 4I). Furthermore, the GPR31-N5Q mutation showed a decreased interaction with Gi3 and a decreased activation of PKCδ-MAPK signaling pathway (Figure 4, J and K). Phenotypic evaluation by BODIPY staining showed that the N158 mutant had a similar effect on lipid deposition as the GPR31-WT, but N5 or DNQ mutants almost completely lost the effect on lipid accumulation (Figure 4L and Supplemental Figure 5A). In addition, the overall transcriptional profiles of hepatocytes revealed by RNA-Seq analyses further indicated that the N5Q and DNQ mutations abolished the exacerbating effects of GPR31 on changes in genes associated with lipid metabolic disorder, inflammatory responses, and cell injury (Figure 4M). Thus, GPR31 directly binds to Gi3 in an N5 glycosylation–dependent manner to activate downstream MAPK signaling and promotes MASH progression.

By co-IP–MS/MS assay, we found that STT3A and STT3B, 2 conventional oligosaccharyltransferases capable of protein N-linked glycosylation (26), were among the GPR31 interacting proteins (Supplemental Figure 5, B and C). Intriguingly, through targeted gene silencing, we found STT3A was necessary for N-linked glycosylation at the N158 site of GPR31, and N5 glycosylation specifically required STT3B (Supplemental Figure 5, D and E). Consistent with our functional observations of GPR31 glycosylation, knockdown of STT3B significantly abolished the effect of GPR31 on lipid accumulation and inflammatory gene induction in hepatocytes (Supplemental Figure 5, F and G).

### GPR31 and its glycosylation at N5 are required for MASH progression in mice.

**Figure 5:**
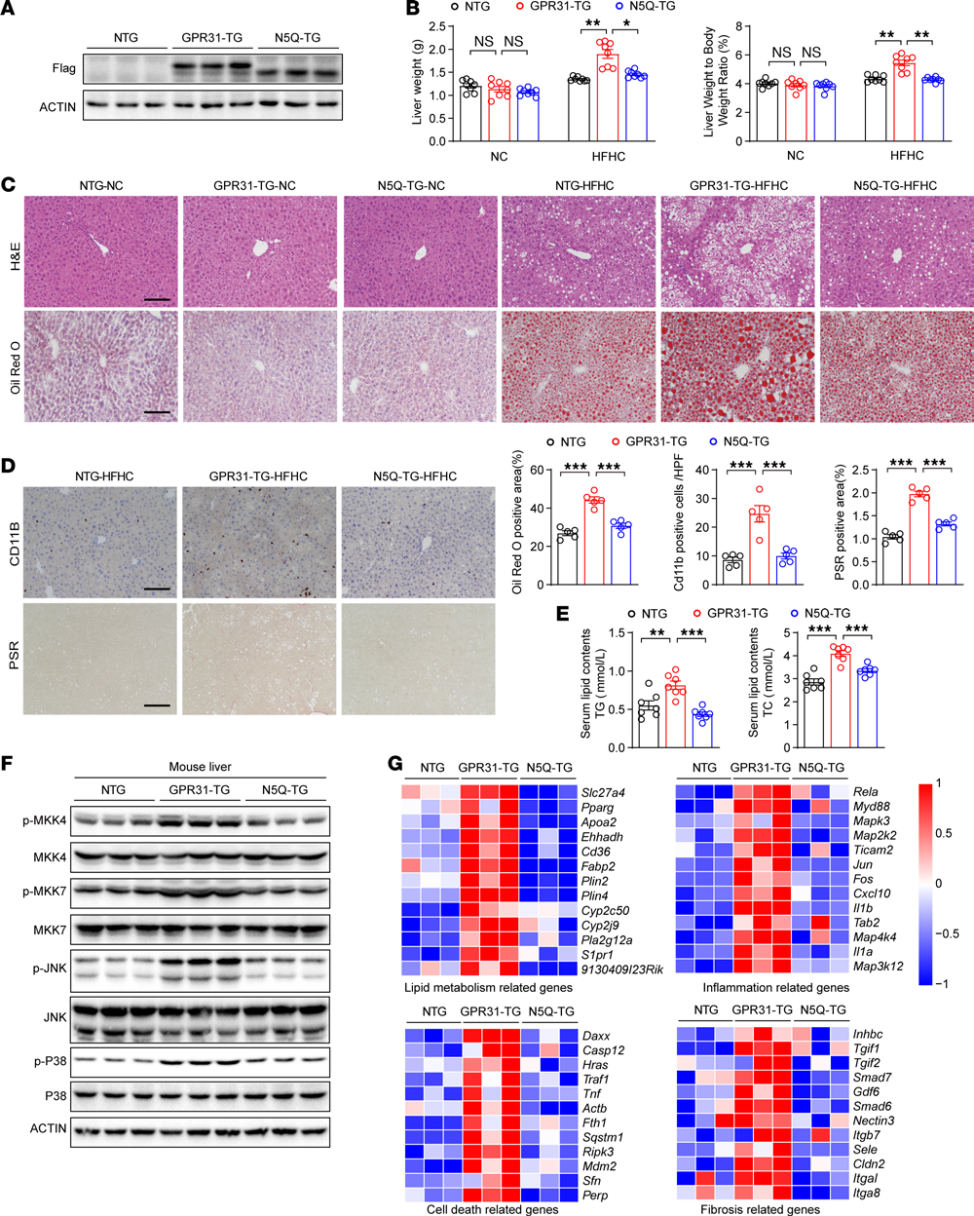
GPR31 and its glycosylation at N5 are required for MASH progression in mice. (**A**) Validation of transgenic overexpression of GPR31-WT and GPR31-N5Q mutant in mice by Western blot analysis. *n* = 3 mice. (**B**) Liver weight and liver weight/body weight ratio in GPR31-WT and GPR31-N5Q transgene mice. *n* = 8 mice. (**C**) H&E and Oil Red O staining of liver sections in GPR31-WT and GPR31-N5Q transgene mice. *n* = 8 mice. Scale bars: 100 μm. (**D**) CD11B and Picrosirius red (PSR) staining of the liver sections of the mice in the indicated group. *n* = 5 mice. Scale bars: 100 μm. (**E**) Effect of GPR31-WT and GPR31-N5Q transgene on serum triglyceride (TG) and total cholesterol (TC) levels. *n* = 7 mice. (**F**) Effect of GPR31-WT and GPR31-N5Q transgene on the phosphorylation of key proteins in the identified MAPK signaling pathway in mouse liver. *n* = 3 mice. (**G**) Transcriptomic profiling of GPR31-WT and GPR31-N5Q transgenic mouse livers. Differentially expressed genes are highlighted. *n* = 3 mice. Data are shown as mean ± SEM. *P < 0.05; **P < 0.01; ***P < 0.001; NS, no significance, *P* > 0.05.

To further explore the role of GPR31 and glycosylation in vivo, we generated transgenic mice with hepatocyte-specific expression of a Flag-labeled *Gpr31* WT protein (*Gpr31*-HepTg mice) or a Flag-labeled *Gpr31*-N5Q mutant (*Gpr31*-N5Q-HepTg mice). Overexpression of these transgenes was confirmed by immunoblotting (Figure 5A). The transgenic mice developed a comparable baseline phenotype to the WT controls under a normal chow diet in terms of liver weight and hepatic histology (Figure 5, B and C). In contrast, after 16 weeks of a high fat/high cholesterol (HFHC) diet, the *Gpr31*-HepTg mice developed significantly increased liver weight and ratio of liver weight to body weight compared with the nontransgenic control mice (Figure 5B). At a histological level, the *Gpr31*-HepTg mice exhibited exacerbated hepatic lipid accumulation, inflammatory cell infiltration, and fibrosis after 16 weeks of HFHC diet feeding (Figure 5, C and D), as well as higher levels of serum triglyceride and total cholesterol (Figure 5E). Furthermore, GPR31 overexpression enhanced the activation of MAPK signaling in livers from mice after 16 weeks of HFHC feeding (Figure 5F and Supplemental Figure 6A). In contrast, overexpression of *Gpr31*-N5Q did not induce aggravation of any of these MASH-associated pathological features or MAPK signaling activation in response to HFHC diet feeding, and MASH severity in the *Gpr31*-N5Q-HepTg mice was comparable to that in the WT mice (Figure 5, B–F). RNA-Seq of liver tissues from the WT and the *Gpr31*-HepTg mice revealed that *Gpr31* overexpression led to robust enrichment of genes implicated in lipid metabolism, inflammation, fibrosis, and cell damage (Figure 5G and Supplemental Figure 6B). However, *Gpr31*-N5Q-HepTg mice showed comparable gene expression profiles as the WT mice (Figure 5G). These findings indicate that GPR31 is a potent driver of MASH progression and that its glycosylation at N5 is indispensable for the GPR31-mediated pro-MASH function in vivo.

To investigate the functional role of Gpr31 in MASH pathogenesis, we generated mice with hepatocyte-specific *Gpr31* deficiency (*Gpr31*-Hep-KO mice; Supplemental Figure 6C), which were subjected to normal chow (NC) or HFHC diet feeding for 16 weeks in parallel with their littermate *Gpr31^fl/fl^* controls. *Gpr31* deficiency significantly reduced liver weight as well as the ratio of liver weight to body weight in response to the HFHC diet, accompanied by marked attenuation of hepatic steatosis, inflammatory cell infiltration, fibrosis, and liver injury (Supplemental Figure 6, D–H). Consistent with the phenotypic observations, the Western blot showed a significantly inhibited MAPK signaling in the *Gpr31*-Hep-KO group compared with the floxed controls (Supplemental Figure 6I). RNA-Seq analysis results also clearly showed that MASH-associated genes in lipid metabolic disorders, inflammatory responses, fibrosis, and cell injury were substantially mitigated in the *Gpr31*-Hep-KO mouse liver tissues compared with the controls (Supplemental Figure 6, J and K). Notably, hepatic deletion of *Gpr31* did not induce any gross phenotypic or histological changes in mice on the NC diet (Supplemental Figure 6, D–H).

### G4451 is an anti-MASH small molecule via blockade of GPR31-Gi3 interaction.

**Figure 6:**
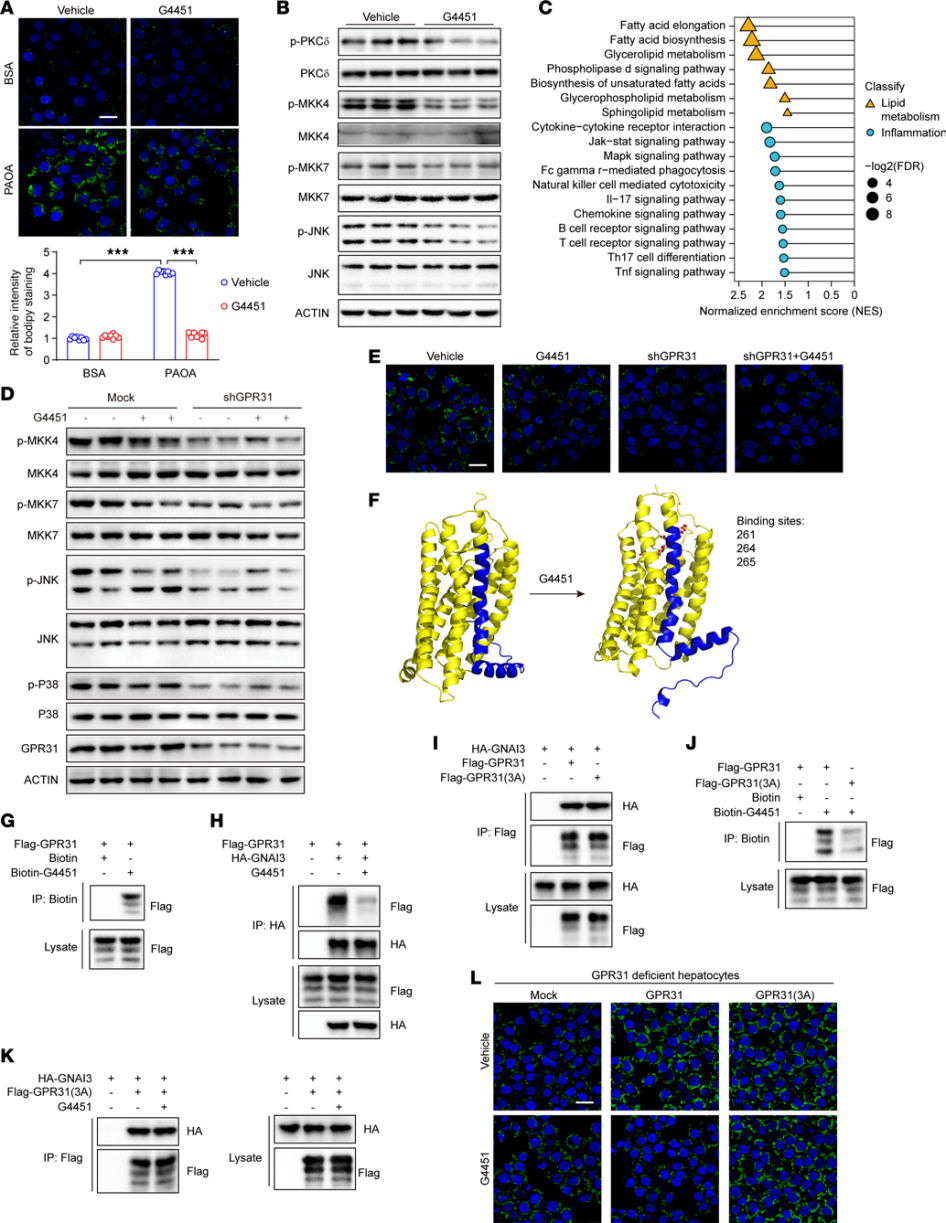
G4451 was developed as an anti-MASH candidate via blockade of the GPR31-Gαi3 interaction. (**A**) BODIPY staining and quantitative results of lipid droplets in hepatocytes with or without G4451 treatment. Scale bars: 30 μm. *n* = 3 with 8 images quantified. (**B**) Effect of G4451 on the phosphorylation of key proteins in the MAPK signaling pathway in hepatocytes. *n* = 3. (**C**) Enriched pathway analysis of hepatocytes with or without G4451 treatment. *n* = 3. (**D**) Western blotting showing the phosphorylation of key proteins in the MAPK signaling pathway in WT and GPR31-deficient hepatocytes with or without G4451 treatment. *n* = 3. (**E**) BODIPY staining of lipid droplets in WT and GPR31-deficient hepatocytes with or without G4451 treatment. Scale bars: 30 μm. *n* = 3. (**F**) The molecule docking between GPR31 and G4451 complex. (**G**) Co-IP of GPR31 and biotin-G4451. *n* = 3. (**H**) Effect of G4451 on the interaction of GPR31 and GNAI3. *n* = 3. (**I**) Co-IP of GNAI3 and GPR31 or its docking mutants GPR31(3A). *n* = 3. (**J**) Co-IP of biotin-G4451 and GPR31 or its docking mutants GPR31(3A). *n* = 3. (**K**) Effect of G4451 on the interaction of GNAI3 and GPR31(3A). *n* = 3. (**L**) BODIPY staining of lipid droplets in GPR31-deficient hepatocytes with GPR31 overexpression, GPR31(3A) overexpression, or G4451 treatment. Scale bars: 30 μm. *n* = 3. Data are shown as mean ± SEM. ***P < 0.001; NS, no significance, *P* > 0.05.

Considering the robust effects of GPR31 on promoting MASH as well as the highly druggable features of GPCRs (6, 7), we designed a series of small-molecule candidates to target GPR31. First, the potential protein structure of human GPR31 was simulated by 3D structure homology modeling, and 18 potential small-molecule inhibitors were designed based on in-silico fitting (Supplemental Figure 7A). We then evaluated the efficacy of those 18 candidates in inhibiting hepatocyte lipid deposition upon PA/OA stimulation using high-content screening. Notably, a candidate inhibitor, G4451, showed the greatest inhibition of PA/OA-induced lipid accumulation in hepatocytes (Supplemental Figure 7, B and C). We validated the robust inhibitory effects of G4451 on lipid accumulation, inflammatory response, and MAPK signaling upon PA/OA treatment in primary hepatocytes (Figure 6, A–C, and Supplemental Figure 7D). Importantly, we also demonstrated that such G4451-mediated inhibitory effects were dependent on GPR31 expression (Figure 6, D and E, and Supplemental Figure 7, E and F).

To clarify the detailed pharmacological mechanisms by which G4451 inhibits GPR31-regulated MASH, we reperformed additional molecular docking analysis for the GPR31-G4451 complex. G4451 showed potential interactions with GPR31 at aa 261, 264, and 265 via hydrogen bonds, leading to marked allosteric change at the C-terminal loop of GPR31 (Figure 6F). This direct binding of G4451 to GPR31 was confirmed by co-IP using biotin-linked G4451 and a Flag-GPR31 (Figure 6G). Notably, the binding of G4451 to GPR31 greatly decreased the GPR31-Gαi3 interaction (Figure 6H). When aa 261, 264, and 265 were mutated to alanine (3A), the mutant GPR31 maintained its interaction with Gαi3 but lost its capacity to interact with G4451 (Figure 6, I and J). Consequently, G4451 treatment failed to block the binding of GPR31(3A) mutant to Gαi3 (Figure 6K). Finally, G4451 showed no inhibitory effects on GPR31(3A)-exacerbated lipid accumulation, inflammatory responses, and MAPK signaling activation in hepatocytes (Figure 6L and Supplemental Figure 7, G–I).

### G4451 effectively blocks MASH in mice and monkeys.

**Figure 7:**
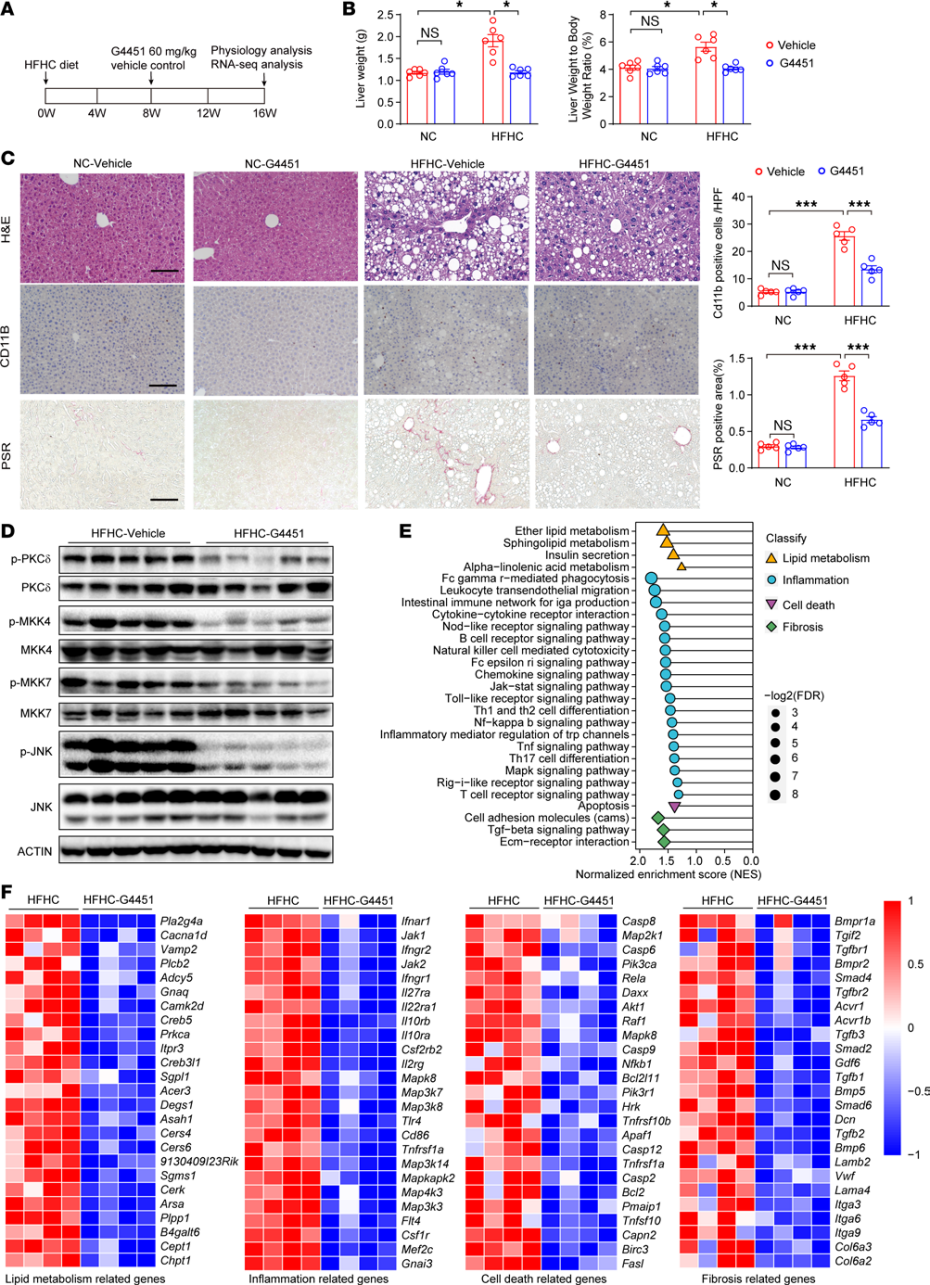
G4451 effectively blocks MASH in mice. (**A**) Timeline for the experimental procedure on mice fed an HFHC diet and treated with vehicle or G4451 (60 mg/kg). (**B**) Liver weight and liver weight/body weight ratio in mice treated with vehicle or G4451. *n* = 6 mice. (**C**) Representative images of H&E, CD11B, and Picrosirius red (PSR) staining on mouse liver sections in G4451- and vehicle-treated mice. Scale bars: 100 μm. *n* = 5 mice. (**D**) Western blot showing the effect of G4451 on MAPK signaling pathway in HFHC-fed mice liver. *n* = 5 mice. (**E**) KEGG pathway enrichment analysis in G4451-treated mouse liver tissues suggest suppressive effects on inflammation, lipid metabolism, cell death, and the fibrosis process by G4451. *n* = 4 mice. (**F**) Heatmaps showing differentially expressed genes in pathways of cell death, lipid metabolism, inflammatory response, and fibrosis in liver tissues from HFHC mice treated with vehicle or G4451. *n* = 4 mice. Data are shown as mean ± SEM. *P < 0.05; ***P < 0.001; NS, no significance, *P* > 0.05.

Next, we examined the safety and anti-MASH efficacy of G4451 in mice. To investigate the in vivo dose-response relationship of G4451, C57BL/6J mice were administered daily doses of 15, 30, 60, and 120 mg/kg for 1 week. Significant MAPK inhibitory effects were observed at the 60 mg/kg dose, which was used in vivo (Supplemental Figure 8A). Male C57BL/6J mice were first fed an HFHC diet for 8 weeks and then treated with G4451 or vehicle control for an additional 8 weeks (Figure 7A). Compared with vehicle treatment, the G4451 treatment significantly reduced liver weight and liver lipid accumulation after 16 weeks of HFHC diet feeding (Figure 7, B and C). Furthermore, mice treated with G4451 showed lower hepatic inflammatory cell infiltration and fibrosis than those in the vehicle-treated cohort (Figure 7C), along with blunted activation of PKCδ and MAPK signaling (Figure 7D and Supplemental Figure 8B). The global gene profiles revealed by RNA-Seq clearly indicated that G4451 administration greatly suppressed MASH-related genes (Figure 7, E and F). Moreover, 8 weeks of G4451 treatment did not induce any gross side effects in mice (Supplemental Figure 8, C and D). Pharmacokinetic and toxicological studies demonstrated that the *t*_1/2_ of G4451 oral administration is 5.35 hours, and it is mainly distributed in the liver, kidney, and white fat tissues (Supplemental Figure 8, E and F).

**Figure 8:**
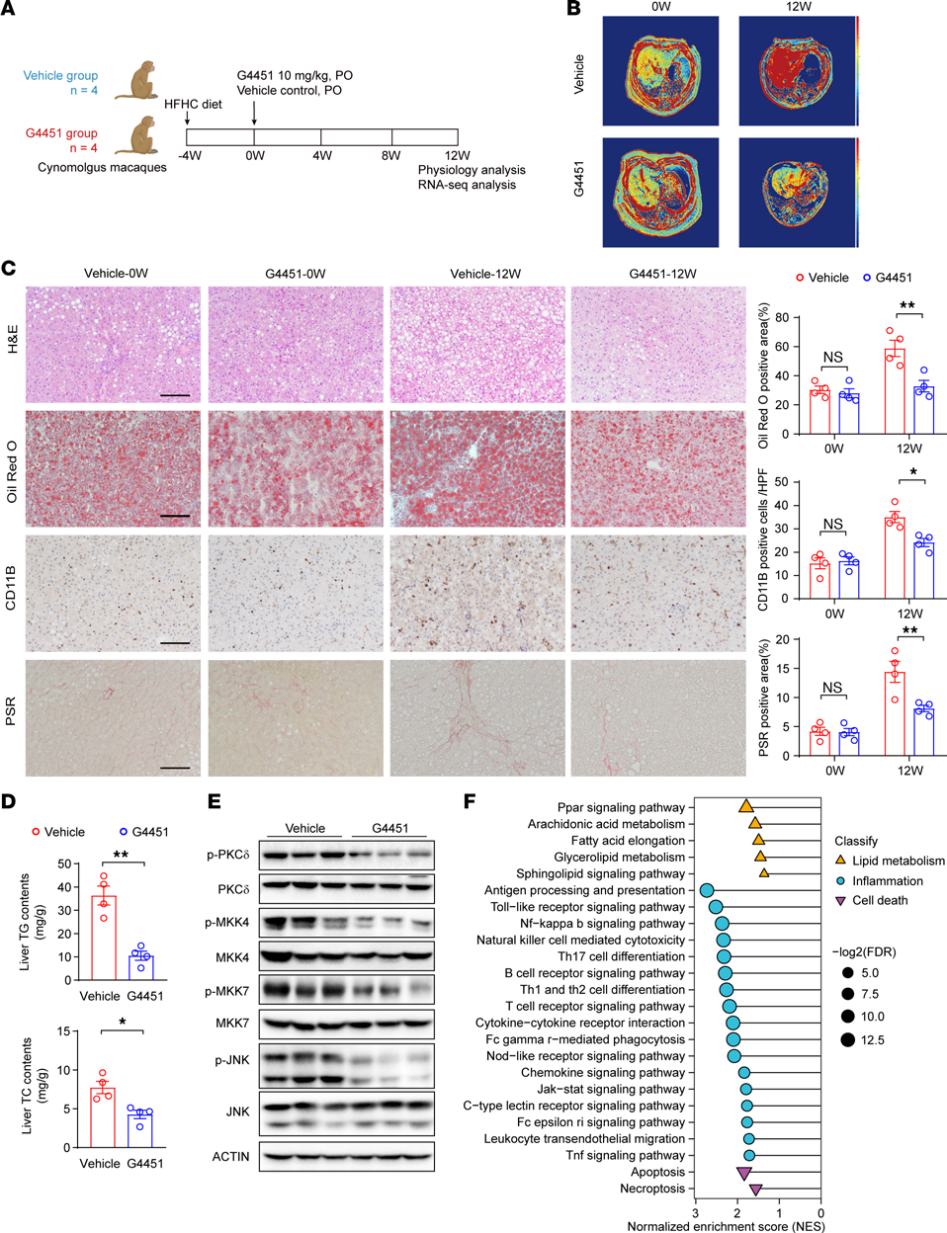
G4451 effectively blocks MASH in monkeys. (**A**) Illustration of the experimental procedure on monkeys fed an HFHC diet and treated with vehicle or G4451 (10 mg/kg/day). *n* = 4 monkeys. (**B**) Representative images of liver MRI from monkeys in week 0 (0W) and week 12 (12W) treated with vehicle or G4451. *n* = 4 monkeys. (**C**) Representative images of H&E, Oil Red O, CD11B, and Picrosirius red (PSR) staining on monkey liver sections in G4451- and vehicle-treated monkeys. Scale bars: 100 μm. *n* = 4 monkeys. (**D**) Triglyceride (TG) and total cholesterol (TC) content in 12W monkey liver tissues treated with vehicle or G4451. *n* = 4 monkeys. (**E**) Western blot showing the effect of G4451 on MAPK signaling pathway in HFHC-fed monkey liver. *n* = 3 monkeys. (**F**) KEGG pathway enrichment analysis in G4451- and vehicle-treated monkey livers. *n* = 3 monkeys. Data are shown as mean ± SEM. *P < 0.05; **P < 0.01; ***P < 0.001; NS, no significance, *P* > 0.05.

Finally, to evaluate the translational importance, we tested the therapeutic effect of G4451 in a diet-induced MASH model in nonhuman primates. A total of 8 cynomolgus macaques (*Macaca fascicularis*) with spontaneously developed MASLD were subjected to HFHC diet feeding for 4 weeks to further increase MASLD severity. The macaques were then randomly separated into 2 groups and orally administered G4451 at 10 mg/kg/d or vehicle for another 12 weeks (Figure 8A and Supplemental Figure 9A). The physical indexes were comparable between the G4451 and vehicle-treated groups at the baseline before administration (week 0) (Supplemental Table 1).

Notably, MRI and histological analysis indicated that G4451 treatment markedly alleviated hepatic steatosis, steatohepatitis, and liver fibrosis in HFHC-fed monkeys (Figure 8, B and C). Consistent with this finding, G4451 treatment effectively decreased the liver triglyceride and total cholesterol content (Figure 8D). But changes in blood biochemical indexes were not significant, possibly due to the relatively short drug treatment duration (Supplemental Table 1). We did not find any unfavorable changes in phenotypic features after 12 weeks of G4451 administration (Supplemental Table 1). At the molecular level, Western blot analysis showed a robust inhibition of MAPK signaling in the G4451-treated monkey livers (Figure 8E and Supplemental Figure 9B), and RNA-Seq indicated substantial reversal of transcriptome reprogramming related to lipid metabolism, cell death, and inflammation induced by HFHC diet feeding (Figure 8F and Supplemental Figure 9C). Altogether, these data indicated that this small-molecule inhibitor targeting GPR31-Gαi3 interaction is a promising therapeutic regimen for MASH.

## Discussion

MASLD has emerged as a silent but increasingly prevalent form of chronic liver disease with unmet treatment needs (27). In this study, leveraging both unbiased transcriptomic profiling and functional screening, we characterized a subfamily of metabolite-responsive GPCRs in MASH and identified GPR31 as a notable driver of MASH progression across different species. GPR31 exhibits conserved upregulation in humans, monkeys, and mice during MASH progression. The induction of GPR31 expression markedly aggravates steatohepatitis, and this detrimental effect is mediated through downstream interaction with Gαi3. Notably, we have identified a small-molecule inhibitor targeting GPR31 via potential conformational change in its C-terminus and interruption of GPR31-Gαi3 interaction. The safety and therapeutic efficacy of this GPR31 inhibitor were validated in both mouse and monkey preclinical models of MASH. Therefore, our study provides a therapeutic target and unconventional pharmacological strategy for MASH therapy.

Our knowledge of MASH pathobiology has been advanced substantially over the past 4 decades, but progress in approved pharmacotherapies is still slow (28). Despite the existence of various anti-MASH targets (e.g., ASK1, FGF19, FGF21, SCD1, ACC, FXR, and PPAR) and the drugs in the current pipeline of clinical studies, only one anti-MASH drug, resmetirom, has been approved by the FDA; a comprehensive evaluation of adverse events, effectiveness, cost-effectiveness, social acceptance, and accessibility is still needed (29). GPCRs are proven to be the best drug targets that can exert a wide range of pathophysiological functions (6). Currently, one-third of the drugs approved by the FDA act on 108 unique GPCR targets (6). In addition to the described importance of GPCRs in the immune and central nervous systems, accumulating evidence indicates the high relevance of GPCRs in metabolic tissues (islets, adipose tissue, liver, and intestine), and the disease indications for GPCR modifiers have implicated many common metabolic diseases, including diabetes, obesity, and fatty liver (6, 30). In this study, we focused on metabolite-sensing GPCRs, which function as the direct entry points of signaling for a variety of nutrient metabolites, such as free fatty acids, bile acids, lactate, cholesterol, and chemokines (8, 31, 32). Using integrated multimodal screening of metabolite-sensing GPCRs, we identified GPR31 as a key driver of pathological events in MASH.

GPR31 was initially identified as the high-affinity cognate receptor for the proinflammatory and bioactive lipid product 12-HETE, derived from ALOX12 (33, 34), a member of the extracellular acid–sensitive GPCR family (35). Increasing evidence has suggested that GPR31 plays an important role in diverse pathophysiological processes, such as pancreatic development (36), atherosclerosis (37), prostate cancer (38), hepatocellular carcinoma (39), platelet activation, and arterial thrombosis (40). Our recent study also demonstrated that the ALOX12-12-HETE-GPR31 axis is critical for hepatic ischemia/reperfusion injury (41). Here, we provided evidence indicating that GPR31 robustly promotes MASH development. The pathophysiological functions of GPR31 in MASH are largely mediated by the direct interaction of GPR31 with Gαi3 to activate downstream PKCδ-MAPK activation. The molecular discrimination of GPR31 under conditions of acute versus chronic liver damage might be largely due to the different pathological stimuli involved, a possibility that requires further investigation.

Given the lack of effective pharmacotherapy for MASH, it is important to explore multiple therapeutic approaches. Interestingly, and of potential therapeutic importance, we have identified a small-molecule inhibitor, G4451, which functions in a possible allosteric mechanism to block GPR31-Gαi3 interaction. In vivo testing in preclinical models from mice to monkeys indicated that G4451 is potentially safe and efficacious in suppressing MASH development, strongly arguing for G4451 as a promising drug candidate for MASH treatment. Preliminary pharmacokinetic and toxicological findings indicate the promising developmental potential of G4451. The drug-like properties and dosing strategy of compound G4451 require further chemistry, manufacturing, and controls and pharmacokinetic studies.

In addition, we also uncovered a mechanism of regulation for GPR31 activity via glycosylation at residue N5 and demonstrated that N5 glycosylation is essential for its proper targeting to cytomembrane and GPR31-Gαi3 interaction. Indeed, mutation of the GPR31 glycosylation site N5 largely reversed the phenotypic and molecular effects of GPR31 on MASH. This evidence is consistent with the notion that glycosylation is a common and important posttranslational modification for other proteins involved in MASLD/MASH, including ChREBP, CD36, and apolipoprotein B100 (42, 43). Our study reiterates the importance of targeting glycosylation and subsequent molecular events in treating metabolic diseases.

In conclusion, the present study provides proof-of-concept evidence that GPR31 induction strongly promotes MASH development by directly binding to Gαi3 to activate downstream MAPK signaling under metabolic overload. More importantly, we developed a small molecule that can specifically block the GPR31-Gαi3 interaction and robustly reverse lipid steatosis, inflammation, and fibrosis in both mice and monkeys. Thus, the outcome of this study expands the current mechanistic framework in MASH pathophysiology and demonstrates the conceptual feasibility of targeting metabolite-sensing GPCR. The glycosylation and interaction of GPR31with downstream G protein can serve as promising interventional targets for MASH therapy.

## Methods

### Sex as a biological variable.

Human samples were deliberately sourced from male and female individuals, with approximately equal representation. Analysis confirmed that the core findings and conclusions were consistent across both sexes. For mouse experiments, to avoid interference from estrogen, only male animals were used in this study. This decision was made because female mice exhibit greater interindividual variability in metabolic parameters compared with males, primarily due to the critical impact of estrogen and estrogen receptor signaling on hepatic lipid metabolism, a core pathological process in MASH.

### Cell lines.

The cell lines Huh7, HepG2, and HEK293T were purchased from the National Collection of Authenticated Cell Cultures. Cells were cultured in DMEM (Gibco, C11995) supplemented with 10% FBS (Tico, FBS EU500) and 1% penicillin-streptomycin (Gibco, 15140-122) in a 5% CO_2_ incubator with controlled humidity. To establish a cell model of hepatic steatosis/inflammation/lipotoxicity, mouse primary hepatocytes or Huh7 cells were stimulated with PA (0.5 mM; P0500, Sigma-Aldrich) dissolved in 0.5% fatty acid–free BSA (BAH66-0100, Equitech Bio) for the indicated times. Fatty acid–free BSA (0.5%) alone was used as a vehicle control.

### Plasmid construction.

All full-length, truncated, and mutant GPR31, G protein, STT3A, STT3B, and PKC constructs from the corresponding coding sequences were amplified from human cDNA and inserted into pcDNA5 or a phage vector. The HA or Flag tag was fused to the N-terminus of the protein. shRNA was inserted into pENTR vector with a U6 promoter. All plasmid DNA sequences were verified by sequencing (Tsingke). The sequences for shRNA are in Supplemental Table 2.

### Western blot analysis.

Lysates from tissues or cultured cells were collected in RIPA lysis buffer (P0013E, Beyotime) and sonicated on ice. Then, a BCA kit (23225, Thermo Fisher Scientific) was used to quantify the total protein concentration. Protein lysates were separated by 8%–10% SDS-PAGE and transferred to PVDF membranes (IPVH00010, MilliporeSigma). The membranes were then blocked with 5% skim milk for 30 minutes before incubation with primary antibodies overnight at 4°C and subsequent incubation with HRP-conjugated secondary antibodies. The information for the primary antibodies is as follows: Antibody specific for GPR31 was procured from Abcam (ab75579). Antibodies specific for β-actin (4967), phospho-JNK (Thr183/Tyr185) (4668), JNK (9252), phospho-p38 MAPK (Thr180/Tyr182) (4511), p38 (9212), phospho-MKK4 (Ser257/Thr261) (9156), MKK4 (9152), phospho-MKK7 (Ser271/Thr275) (4171), MKK7 (4172), phospho-PKCδ (Thr505) (9374), PKCδ (2058), phospho-PKA C (Thr197) (4781S), and Na,K-ATPase α1 (23565) were procured from Cell Signaling Technology. Antibodies specific for HA (TP00973GeA10) and Flag (TP00975GeA10) were procured from Biolight WellAnimal. Antibodies specific for GNAI3 (A13307), GNAS (A5546), GNB1 (A1867), and GNB2 (A9643) were procured from ABclonal. Antibodies specific for STT3A (12034-1-AP), STT3B (15323-1-AP), and PKA C α (67491-1-Ig) were procured from Proteintech.

### IP assay.

HEK293T cells were transfected with the indicated plasmids for 24 hours and were then lysed with IP lysis buffer (20 mM Tris-HCl, 150 mM NaCl, 1 mM EDTA, and 1% NP-40; pH 7.4) containing protease inhibitor cocktail (P8340, Sigma-Aldrich). After centrifugation at 4°C and 12,000*g* for 10 minutes, the supernatants were incubated with protein A/G agarose beads (11719386001, Roche) and specific antibodies for 4 hours at 4°C. The beads were then washed with IP lysis buffer 3 times for 5 minutes each. Then, the bead-interacting protein complexes were boiled in SDS loading buffer at 95°C for 10 minutes. Finally, the interacting proteins were identified by Western blotting.

### Quantitative PCR analysis.

Total RNA was extracted with TRIzol reagent (T9424, Sigma-Aldrich), and cDNA was then synthesized from 2 μg of RNA using HiScript III RT SuperMix for qPCR (+gDNA Wiper) (Vazyme, R323-01). Quantitative real-time PCR (qPCR) was performed with ChamQ SYBR qPCR Master Mix (Vazyme, Q341-02) in a LightCycler 480 system (Roche). The housekeeping gene β-actin was used as a control. The primers used in this study are listed in Supplemental Table 3.

### Human liver samples.

All individuals included in this study were free of excessive alcohol consumption, drug abuse, or hepatitis virus infection. Because the exact definition of liver pathology for MASLD is lacking, we continue to apply the definition established for nonalcoholic fatty liver disease (NAFLD). Samples with a NAFLD activity score (NAS) of 5 or higher were classified as MASH samples. Samples with a NAS of 2 or lower but without hepatic steatosis were classified as no MASLD samples. MASL was classified as NAS of between 3 and 4 (3 ≤ NAS ≤ 4). Two pathologists independently evaluated the NAS in a blinded manner. All individuals were Chinese, and donor characteristics are shown in Supplemental Table 4.

### Animals.

Male C57BL/6J mice (8–10 weeks) were purchased from GemPharmatech Co., Ltd. The mice were housed in a temperature-controlled environment (23°C ± 2°C) under specific pathogen–free conditions on a 12-hour light/12-hour dark cycle. The mice were fed an HFHC diet (protein, 14%; fat, 42%; carbohydrates, 44%; cholesterol, 0.2%; TP 26304, Trophic Diet) for 16 weeks to establish MASH pathology. A normal chow diet (containing 10% fat, 70% carbohydrate, and 20% protein; D12450B, Research Diets) served as a control. At the experimental endpoint, blood samples were collected for measurement of biochemical indexes.

To assess the safety of the GPR31 inhibitor G4451 in mice, male 8-week-old C57BL/6J mice were treated by HFHC for 6 weeks followed by orally administered G4451 (60 mg/kg/d, in 0.5% CMC-Na) for 10 weeks. The heart, liver, spleen, lung, and kidney tissues of vehicle- and G4451-treated mice were collected for histological analysis.

Hepatocyte-specific GPR31-KO mice were generated by knocking out the relevant coding gene GPR31b, instead of pseudogenes (Gpr31a, c or Gm6553), using CRISPR/Cas9 technology. According to the gene structure, the exon1 of Gpr31b-201 (ENSMUST00000091648.3) transcript is selected as the knockout region that contains all coding sequences. In brief, the sgRNA (Supplemental Table 5) was transcribed in vitro, and donor vector was constructed. The Cas9, sgRNA, and donor vector were microinjected into the fertilized eggs of C57BL/6J mice. The eggs were then transplanted to pseudo-pregnant female mice to obtain positive F0 mice. The genotype of F0 mice was confirmed by PCR and sequencing. The stable F1 generation mice were obtained by mating positive F0 generation mice with C57BL/6J mice, and the Gpr31b-floxed homozygote generation was obtained by mating heterozygote generation mice. At 2 weeks after birth, the toe tissues of mice were collected, and genomic DNA was extracted for the GPR31b*^fl/fl^* mice genotyping assay. GPR31b-Hep-KO mice were generated by mating GPR31b*^fl/fl^* mice with albumin-Cre transgenic mice (003574; The Jackson Laboratory). The liver tissues of mice were collected and genomic DNA was extracted for the genotyping assay of GPR31b-Hep-KO mice.

Considering the gene similarity of GPR31b with pseudogenes, the sgRNA sequences were well-designed to guarantee the specific knockout of GPR31b. Multiple PCR primer pairs were used for mouse strain identification. Primers of LoxP1-F1 and LoxP1-R1; LoxP1-F3 and LoxP1-R3; LoxP2-F2 and LoxP2-R2; and LoxP2-F4 and LoxP2-R4 were used to identify the genotypes of GPR31b*^fl/fl^* mice by collecting mouse toe tissue. Primers of Alb-Cre-C and Alb-Cre-W and of Alb-Cre-C and Alb-Cre-K were used to identify Alb-Cre mice by collecting mouse toe tissue. Primers of Hep-KO-F1 and Hep-KO-R2 and of Cyc-F5 and Cyc-R5 were used to identify the genotypes of GPR31-Hep-KO mice by collecting mouse liver tissue (Supplemental Table 6).

Liver-specific GPR31-WT and GPR31-N5Q transgenic mice were generated using a previously described strategy (44). To generate the hepatocyte-specific GPR31-WT (GPR31-HepTg) and GPR31-N5Q (GPR31-N5Q-HepTg) transgenic mice, a Sleeping Beauty transposase system was applied. In brief, a liver-specific pT3 plasmid carrying GPR31 (pT3-alb-3xflag-h-GPR31) (30 mg per mouse) and the SB100X transposase plasmid (2 mg per mouse) were injected into mice via the tail vein.

For experiments involving nonhuman primates, 8 male cynomolgus monkeys (*Macaca fascicularis*) 8–12 years old with body weights of 6–12 kg were purchased from Topgene Biotechnology. The monkeys were purchased following the legal and regulatory guidelines stipulated by the Chinese government and were approved by both the Department of Forestry of Hubei Province and Guangdong Province. Monkeys that passed the physical examination and met the standards of the quarantine inspection were used. The monkeys were screened by liver biopsy, and the NAS was evaluated by 2 independent pathologists in a blinded manner. Monkeys with hepatic steatosis were selected for experiments. The monkeys were randomly assigned to the vehicle or G4451 group (*n* = 4 monkeys per group). The monkeys were fed an HFHC diet (containing lard, 10%; sucrose, 15%; cholesterol, 1%; corn flour, 14.5%; wheat flour, 14.5%) to accelerate MASH development. G4451 (10 mg/kg, dissolved in 1% DMSO and 15% β-cyclodextrin in saline) or vehicle was orally administered to the monkeys daily. Monthly physical examinations were performed, and liver biopsy and MRI were performed after these examinations. At the experimental endpoint, the monkeys were fasted overnight and anesthetized with Zoletil and xylazine (5:2, v/v; 0.03 mL/kg) before liver biopsy or MRI as previously described (45).

### Primary hepatocyte isolation.

Primary hepatocytes were isolated from 8-week-old male C57BL/6 mice by a collagenase perfusion and gradient centrifugation method, as previously described (44). In brief, the liver was perfused with liver perfusion medium (17701-038, Thermo Fisher Scientific) and digested with liver digestion medium (17701-034, Thermo Fisher Scientific). Then, the liver tissues were filtered through a 70 μm cell strainer (352350, Falcon). After that, the cell suspension was centrifuged at 50*g* for 3 minutes and was then cultured in DMEM supplemented with 10% FBS and 1% penicillin-streptomycin at 37°C. Plates coated with rat tail collagen overnight were prepared for seeding.

### Biochemical analysis.

Animal serum was prepared for analysis of triglyceride, total cholesterol, alanine aminotransferase (ALT), and aspartate aminotransferase (AST) using an automatic ADVIA 2400 Biochemical Analyzer (Siemens) according to the manufacturer’s instructions.

### Liver lipid analysis.

Liver tissues were homogenized and centrifuged. Then, the supernatants were used for analysis of triglyceride and total cholesterol content with commercially available kits (290-63701 for triglyceride and 294-65801 for total cholesterol, Wako) according to the manufacturer’s instructions.

### Histological analysis.

Liver tissues from mice and monkeys were divided and fixed overnight with 10% formalin for paraffin sectioning and H&E staining or embedded in OCT compound and cryosectioned for Oil Red O staining (O0625, Sigma-Aldrich). The extent of liver fibrosis was assessed by Picrosirius red (26357-02, Hede Biotechnology) staining of paraffin sections.

### Immunohistochemistry.

For immunohistochemical analysis of CD11b, tissue sections were permeabilized with 0.1% Triton X-100 before blocking with normal goat serum for 30 minutes. Then, the sections were incubated with anti-CD11b antibodies (BM3925, Boster Biological Technology) at 4°C overnight before incubation with secondary antibodies. Images were acquired with a light microscope (Olympus). At least 8 different optical fields per animal were imaged.

### BODIPY staining.

For BODIPY staining of neutral lipid droplets for microscopy, hepatocyte cells were treated with 0.5 mM PA (P0500, Sigma-Aldrich) and 1 mM OA for 24 hours. Then, the cells were fixed with 4% paraformaldehyde and stained with BODIPY (D3922, Thermo Fisher Scientific) and Hoechst 33258 (C1011, Beyotime). Lipid accumulation was visualized and quantified by a laser scanning confocal microscope (TCS SP8, Leica) or an Operetta CLS high-content analysis system (PerkinElmer).

For BODIPY staining for quantification by flow cytometry, Huh7 cells were incubated with BODIPY staining solution for 15 minutes in the dark at 37°C after treatment with 0.5 mM PA and 1 mM OA for 16 hours. The cells were then resuspended in flow cytometry buffer, and flow cytometry (Accuri TMC6 plus, BD Biosciences) was performed for quantification of intracellular neutral lipid droplets.

### Colocalization.

Briefly, Huh7 cells were transfected with Flag-tagged GPR31 or GPR31 mutant (N5Q, N158Q, and DNQ) plasmids for 18 hours. After fixation, permeabilization, and blocking, the cells were incubated with anti-Flag and anti-Na/K ATPase antibodies for 3 hours at room temperature, followed by washing and incubation with fluorescent secondary antibodies for 1 hour. Nuclei were labeled with DAPI. Confocal microscopy was performed at room temperature using a laser-scanning confocal microscope (TCS SP8, Leica).

### Pharmacokinetics.

For pharmacokinetic characteristics, male Wistar rats at the age of 8–10 weeks were used for the pharmacokinetics assay. G4451 was delivered to the rats by intragastric administration at the dose of 50 mg/kg or by intravenous administration at a dose of 1 mg/kg. Plasma samples were collected before and at 0.083, 0.167, 0.5, 1, 2, 4, 8, 12, and 24 hours after G4451 administration. G4451 concentrations in plasma were examined by liquid chromatography–MS/MS assay. For the tissue accumulation assay, male C57BL6/J mice were treated with G4451 at the dosage of 50 mg/kg. Since the maximum absorption time point in plasma is about 2 hours after G4451 oral administration, major tissues were collected from mice at 2 hours after drug treatment for G4451 tissue distribution.

### Toxicity studies.

For the cell chromosomal aberration test, we treated CHO cells with G4451 at 17, 50, and 150 μg/mL for 6 hours using cyclophosphamide as a positive control. Then, 4 μg/mL colchicine was administered 4 hours before the cells were fixed and stained for chromosomal aberration calculation. For the bone marrow micronucleus test, we treated mice with G4451 at 240, 700, and 2,000 mg/kg for 2 days using cyclophosphamide as a positive control. Mice were euthanized at 6 hours after the last G4451 administration, and bone marrow samples were collected for micronucleus number calculation.

### Computational virtual screening.

The 3D structure of human GPR31 protein (319 amino acids; UniProt, O00270) was reconstructed by I-TASSER. The GPR31 docking grid was maximized for 5 million compounds from Chemdiv and Enamine for subsequent molecular docking.

PDB files were converted to the PDBQT format as macromolecules before virtual screening. The grid (ligand docking search space) was located as described above. Then, Autodock Vina 1.1.2 was used for the subsequent molecular docking. Protein-ligand interactions were visualized using Pymol version 1.7.4.5.

### The structure information and synthesis procedure for G4451.

The structure information and synthesis procedure for G4451 is shown in Supplemental Figure 10. The structure of G4451 is C_32_H_34_F_3_N_5_O_3_; its exact mass is 593.261 (LC-MS, [M+H]^+^ = 594.1). The ^1^H NMR (400 MHz, DMSO-d6) of G4451 is as follows: δ 10.20 (s, 1H), 8.66 (s, 1H), 8.01 (s, 1H), 7.98–7.92 (m, 2H), 7.81 (d, J = 8.4 Hz, 1H), 7.70 (dd, J = 8.9, 2.5 Hz, 1H), 7.62–7.50 (m, 4H), 7.47 (t, J = 8.0 Hz, 1H), 7.27 (d, J = 7.7 Hz, 1H), 7.11 (d, J = 8.9 Hz, 1H), 3.87 (d, J = 13.1 Hz, 1H), 3.70–3.50 (m, 4H), 3.32–3.20 (m, 4H), 3.16–2.99 (m, 3H), 1.89–1.72 (m, 2H), 1.54 (d, J = 7.3 Hz, 2H), 1.34 (ddd, J = 31.3, 21.5, 4.5 Hz, 4H).

The ^13^C NMR (100 MHz, CD3OD) of G4451 is as follows: δ 171.2, 168.5, 157.4, 148.1, 142.1, 136.0, 134.9, 132.9, 131.9, 131.9 (q, J = 33.9 Hz), 130.3, 129.6, 128.6, 125.6 (q, J = 269.9 Hz), 124.8, 123.8, 121.4 (d, J = 7.3 Hz), 120.0 (d, J = 3.5 Hz), 118.1 (d, J = 3.8 Hz), 56.3, 55.8, 46.7, 43.6, 30.3, 27.2, 26.6, 25.2.

### Mass spectrometry.

GPR31 interactome analysis was performed in human hepatocyte cells with or without overexpression of Flag-tagged GPR31. In brief, cell lysates were immunoprecipitated with an anti-Flag antibody and then subjected to LC-MS/MS analysis. Interacting proteins with more than 2 unique peptides were included for further analysis.

### RNA-Seq and data processing.

Total RNA was extracted as described above and used for cDNA library construction with the MGIEasy RNA Library Prep Kit (1000006384, MGI Tech Co., Ltd.). Single-end libraries were sequenced using a MGISEQ 2000 instrument (BGI Tech). The fragments per kilobase of exon model per million mapped fragments (FPKM) values of genes were calculated with StringTie (version 1.3.3b). Differential gene expression was analyzed with DESeq2 (version 1.2.10). Genes with a fold change of greater than 1.5 and a corresponding adjusted *P* value of less than 0.05 were identified as DEGs. GSEA was performed on the Java GSEA (version 4.0.3) platform with the Signal2Noise metric to generate a ranked list and a gene set permutation type. Gene sets with *P* values of less than 0.05 and FDR values of less than 0.25 were considered statistically significant.

### Statistics.

All data are presented as mean ± SEM and were analyzed by SPSS software. Differences between 2 groups were assessed by unpaired 2-tailed Student’s *t* test. The number of animals required to achieve a value of 0.05 and a 1- β value of 0.8 was predetermined on the basis of preliminary experiments. One-way ANOVA was used for comparisons among more than 2 groups and was followed by Bonferroni’s post hoc test (for data with homogeneity of variance) or Tamhane’s T2(M) post hoc test (for heteroscedastic data). Differences with *P* values of less than 0.05 were considered significant.

### Study approval.

Human sample collection and use adhered to the principles of the Declaration of Helsinki and were approved by the Renmin Hospital of Wuhan University Review Board or Zhongnan Hospital of Wuhan University Review Board. Written informed consent was obtained from all study participants or their families. A separate table including human donor characteristics has been provided in the supplemental material.

All animal protocols in this study were approved by the IACUC of Renmin Hospital of Wuhan University or by the IACUC of Zhongnan Hospital of Wuhan University. The animals received humane care based on the *Guide for the Care and Use of Laboratory Animals* (National Academies Press, 2011).

### Data availability.

All RNA-Seq data are available in NCBI SRA (Sequence Read Archive) by the following accession numbers: PRJNA1213866 (Figure 1F and Supplemental Figure 1I), PRJNA1213868 (Figure 2, D and I, and Figure 3, B and D), PRJNA1213869 (Figure 2, H and I, and Figure 3, A and C), PRJNA1213778 (Figure 3L and Supplemental Figure 3M), PRJNA1213749 (Figure 4M), PRJNA1213873 (Figure 5G and Supplemental Figure 6B), PRJNA1213750 (Figure 6C), PRJNA1213871 (Figure 7, E and F), PRJNA1213870 (Figure 8F and Supplemental Figure 9C), PRJNA1213779 (Supplemental Figure 3G), PRJNA1213995 (Supplemental Figure 4, E–G), PRJNA1214008 (Supplemental Figure 4, J and K), PRJNA1213865 (Supplemental Figure 5G), and PRJNA1213872 (Supplemental Figure 6, J and K). Values for all data points in graphs are reported in the Supporting Data Values file. Materials such as cell lines and cDNA clones are available from the corresponding authors.

## Author contributions

XJZ, JF, XC, HS, and HY performed experiments, analyzed data, and wrote the manuscript. KW, WL, LB, HD, RT, JS, WQ, and LF performed molecular biological experiments. HT, JZ, ST, ZW, JW, XZ, and TZ performed animal experiments and data analysis. HX and RL performed MRI experiments. JC, PZ, and ZGS helped design the project and edited the manuscript. JJ performed the computer simulation for the protein interactions. XC and TT performed omics analysis. XJZ, YH, YW, and HL designed experiments, edited the manuscript, and supervised the study.

## Supplementary Material

Supplemental data

Unedited blot and gel images

Supporting data values

## Version 1 09/02/2025

Electronic publication
