# Supplementary material for: Integrated screening identifies GPR31 as a key driver and druggable target for metabolic dysfunction–associated steatohepatitis
Source: J Clin Invest. 2025 Sep 2;135(17):e173193. doi: 10.1172/JCI173193 (PMC12404741; doi:10.1172/JCI173193)
Supplement: Supplemental data [file jci-135-173193-s055.pdf]

Supplementary information for

Integrated screening identifies GPR31 as a key driver and druggable target for metabolic dysfunction-associated steatohepatitis

Xiao-Jing Zhang[1,2,*,#], Jiajun Fu[1,#], Xu Cheng[1,#], Hong Shen[3,#], Hailong Yang[1,#], Kun Wang[1], Wei Li[2], Han Tian[2], Tian Tian[1], Junjie Zhou[1], Song Tian[1,4], Zhouxiang Wang[1,4], Juan Wan[1], Lan Bai[1], Hongfei Duan[1], Xin Zhang[1], Ruifeng Tian[5], Haibo Xu[6], Rufang Liao[6], Toujun Zou[1,2], Jing Shi[1], Weiyi Qu[5], Liang Fang[7], Jingjing Cai[1,8], Peng Zhang[1,4], Zhi-Gang She[1,2], Jingwei Jiang[9], Yufeng Hu[1]*, Yibin Wang[10,*], Hongliang Li[1,2,11,*]

[1] State Key Laboratory of New Targets Discovery and Drug Development for Major Diseases, Gannan Innovation and Translational Medicine Research Institute; School of Pharmacy; First Affiliated Hospital; Gannan Medical University, Ganzhou, China.

[2] Department of Cardiology, Renmin Hospital of Wuhan University, Wuhan, China.

[3] State Key Laboratory of Quality Research in Chinese Medicine, Institute of Chinese Medical Sciences, University of Macau, Macao, China.

[4] School of Basic Medical Sciences, Wuhan University, Wuhan, China.

[5] Department of Cardiology, Zhongnan Hospital of Wuhan University, Wuhan, China.

[6] Department of Radiology, Zhongnan Hospital of Wuhan University, Wuhan, China.

[7] Department of Gastroenterology, Huanggang Central hospital of Yangtze University, Huanggang Institute of Translational Medicine, Huanggang, China.

[8] Department of Cardiology, The Third Xiangya Hospital, Central South University, Changsha, China.

[9] Jiangsu key lab of Drug Screening, China Pharmaceutical University, Nanjing, China.

[10] Signature Research Program in Cardiovascular and Metabolic Diseases, Duke-NUS Medical School, Singapore.

[11] Medical Science Research Center, Zhongnan Hospital of Wuhan University, Wuhan, China.

[#]These authors contribute equally

*Corresponding authors.

Hongliang Li, lihl@whu.edu.cn, No.1 Harmony Avenue, Ganzhou, Jiangxi, China, 341000

Xiao-Jing Zhang, zhangxjing@whu.edu.cn, No.1 Harmony Avenue, Ganzhou, Jiangxi, China, 341000

Yibin Wang, yibinwang@duke-nus.edu.sg, Signature Research Program in Cardiovascular and Metabolic Diseases, Duke-NUS Medical School, 8 College Road, Singapore, 169857

Yufeng Hu, huyf@gimi.ac.cn, No.1 Harmony Avenue, Ganzhou, Jiangxi, China, 341000

Supplementary Tables

Table S1. The physical indexes for monkeys at the baseline (0W) and 12 weeks (12W) following G4451 administration.

	0W		12W	
	Vehicle (n=4)	G4451 (n=4)	Vehicle (n=4)	G4451 (n=4)
ALT(U/L)	28.35±13.49	49.33±10.46	41.85±11.58	56.30±23.14
AST(U/L)	27.53±3.54	31.23±1.98	32.28±3.83	37.35±5.58
CREA(uM)	88.14±8.53	108.46±9.14	89.22±6.81	97.2±6.49
UREA(mM)	3.41±0.59	5.74±0.29	3.40±0.82	2.97±0.49
TC(mM)	13.39±3.00	7.12±1.07	17.10±2.65	21.29±0.91
TG(mM)	0.83±0.49	0.26±0.04	1.03±0.57	1.09±0.45
LDH(U/L)	289.80±42.16	305.33±64.29	276.43±33.64	317.68±7.50
CK(U/L)	106.90±45.13	187.65±95.12	75.18±20.42	64.63±4.86
HDL-c(mM)	2.29±0.55	1.07±0.20	1.69±0.44	1.25±0.15
LDL-c(mM)	6.00±2.02	2.26±0.44	8.04±2.10	9.91±0.75

Table S2. Sequence for shRNA.

Gene	Forward primer (5’-3’)
Gpr31	GCCTCATCGTGTTCTGCAATG
Gnas	GCCAAGTACTTCATTCGAGAT
Gnai3	GCAGGAGTGATTAAACGGTTA
Gnb1	GCCATTTGCTTCTTTCCAAAT
Gnb2	GCCCTGCCCATGCCCACACTA
Stt3a	GCATGCTACTCACCTACTACA
Stt3b	GCAGGTGCTGTGTTCCTTAGT

Table S3. Primers used for qPCR.

Human.

Gene	Forward primer (5’-3’)	Reverse primer (5’-3’)
ACTB	GTGACGTTGACATCCGTAAAGA	GCCGGACTCATCGTACTCC
GPR31	ACCAGGTGCCACAGTTTCTAC	CGGAGTCTTTTCTGGAGAGCC
IL6	GAGTAGTGAGGAACAAGCCAGA	AAGCTGCGCAGAATGAGATGA
IL8	CTAGGACAAGAGCCAGGAAGAA	GGGGTGGAAAGGTTTGGAGTA
TNF	TGGCGTGGAGCTGAGAGATA	TGATGGCAGAGAGGAGGTTG

Table S4. The physical indexes for clinical individuals

	no MASLD	MASL	MASH
Man	7	7	7
Woman	7	7	7
Age	37.5±3.08	35.14±2.92	36.93±2.67
BMI	31.72±0.67	34.11±1.06$^{\#\#\#}$	38.25±1.14***
Systolic pressure (mmHg)	110±3.75	110.85±0.85	110±0
Diastolic pressure (mmHg)	72.38±1.85	74.85±0.15	75±0
Total bilirubin (μmol/L)	11.12±1.08	15.04±2.04	13.71±1.7
Direct bilirubin (μmol/L)	3.44±0.74	2.51±0.25	2.36±0.28
Indirect bilirubin (μmol/L)	8.09±1.03	12.52±1.82	11.36±1.44
Alanine aminotransferase ALT (U/L)	30.07±5.7	28.5±3.9$^{\#}$	54.79±10.94*
Aspartate aminotransferase AST (U/L)	37.11±12.05	24.71±3.36	32.64±4.83
Alkaline phosphatase ALP (U/L)	88.4±9.95	85.36±5.46	87.29±6.63
Gamma glutamyltransferase (U/L)	31.77±4.85	30.93±4.35	49.86±8.9
Total cholesterol (mmol/L)	4.06±0.41	4.83±0.23$^{\#}$	5.32±0.17*
Triglycerides (mmol/L)	1.29±0.25	1.58±0.22	2.12±0.3**
HDL-c (mmol/L)	1.01±0.13	1.23±0.08	1.08±0.07
LDL-c (mmol/L)	2.52±0.36	3.1±0.2	3.52±0.17
Fasting blood glucose (mmol/L)	5.2±0.24	5.93±0.6	7.94±1.11

* represent comparation to no MASLD. *, $P < 0.05$; **, $P < 0.01$; ***, $P < 0.001$.

# represent comparation between MASL and MASH. #, $P < 0.05$; ###, $P < 0.001$.

Table S5. The sgRNA information of GPR31b and multiple pseudogenes

Official Symbol	Gene ID	sgRNA1+PAM	sgRNA2+PAM
**Gpr31b**	436440	GGCCTCAGAGGAAGTAAATTGGG	AGGAAGGTGATGCATGCCACCGG

Table S6. The identification information and genotyping report for GPR31b-HepKO

PCR No.	Primer	Sequence	Band Size	Sample	Genotype
① LOXP1	F1	CAATCGACTTGGTCTCCTTCCTTG	WT: none Targeted: 434bp	Toe	*Gpr31b*-flox
	R1	CCAACTGACCTTGGGCAAGAACAT			
② LOXP2	F2	GCATTCTACGAAGCATGGTATCC	WT: 158bp Targeted: 261bp		
	R2	TCACAACAGCCAAAAGGCATGAG			
③ LOXP1	F3	AGAGGAAGTAAATTGGGCAG	WT: 81bp Targeted: none		
	R3	ACATGCTCACTCATGCTACCATG			
④ LOXP2	F4	TCAGAGCTTACCCACCGGTG	WT:216bp Targeted:none		
	R4	ACCATGCGACACAGAAACTC			
⑤-1 Alb-Cre	C	TTGGCCCCTTACCATAACTG	WT:351bp Targeted:none		Alb-Cre
	W	TGCAAACATCACATGCACAC			
⑤-2 Alb-Cre	C	TTGGCCCCTTACCATAACTG	WT: none Targeted: 390bp		
	K	GAAGCAGAAGCTTAGGAAGATGG			
⑥ HepKO	F1	CAATCGACTTGGTCTCCTTCCTTG	WT: none Targeted: 575bp	Liver	*Gpr31b*-HepKO
	R2	TCACAACAGCCAAAAGGCATGAG			
⑦ Cyc	F5	ATGTGCCATTTCACCGTGAC	WT: none Targeted: 649bp		
	R5	CCTCAATCCCAGAGTCTGCTTT			

52 # Supplementary Figure

Figure S1

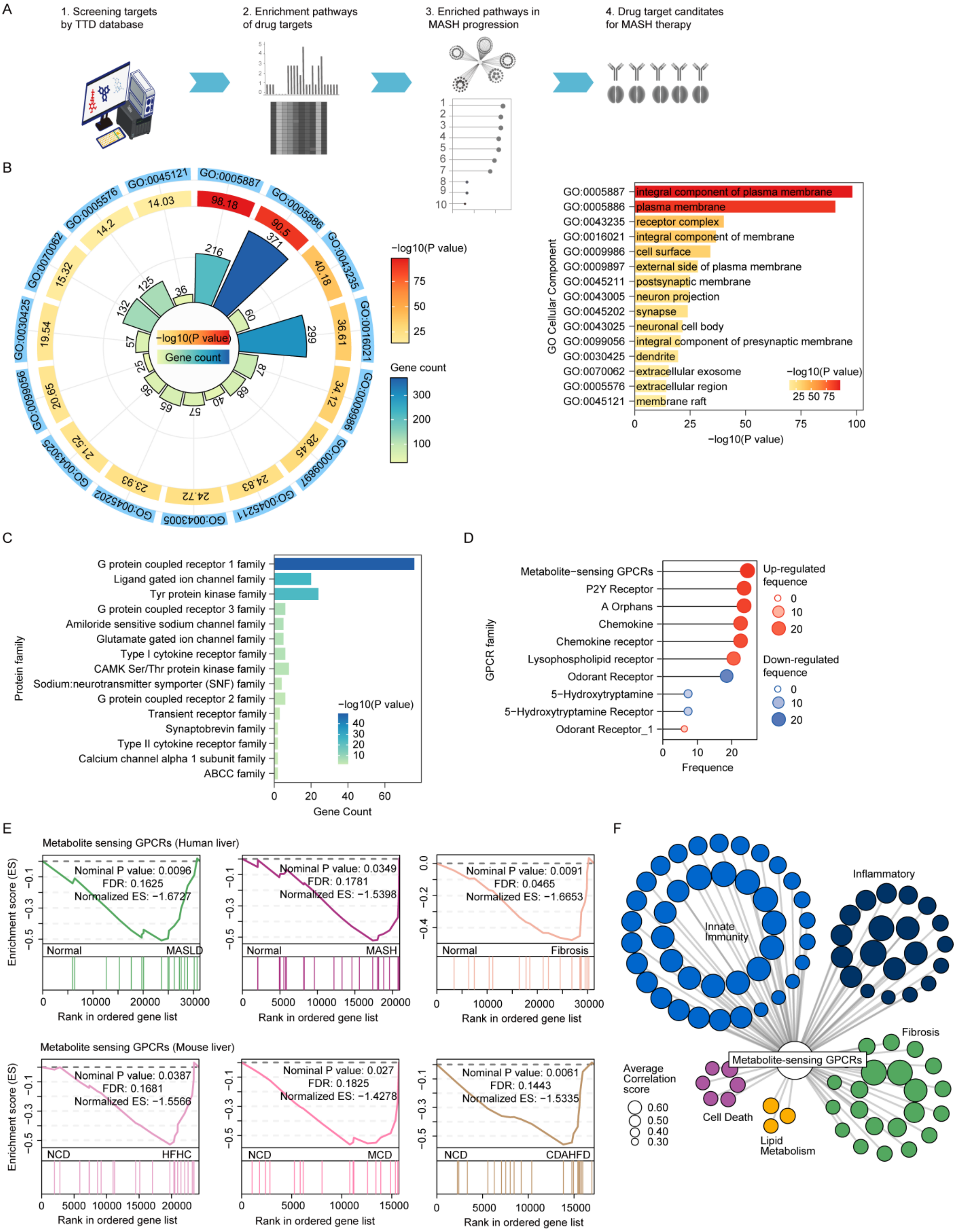

53

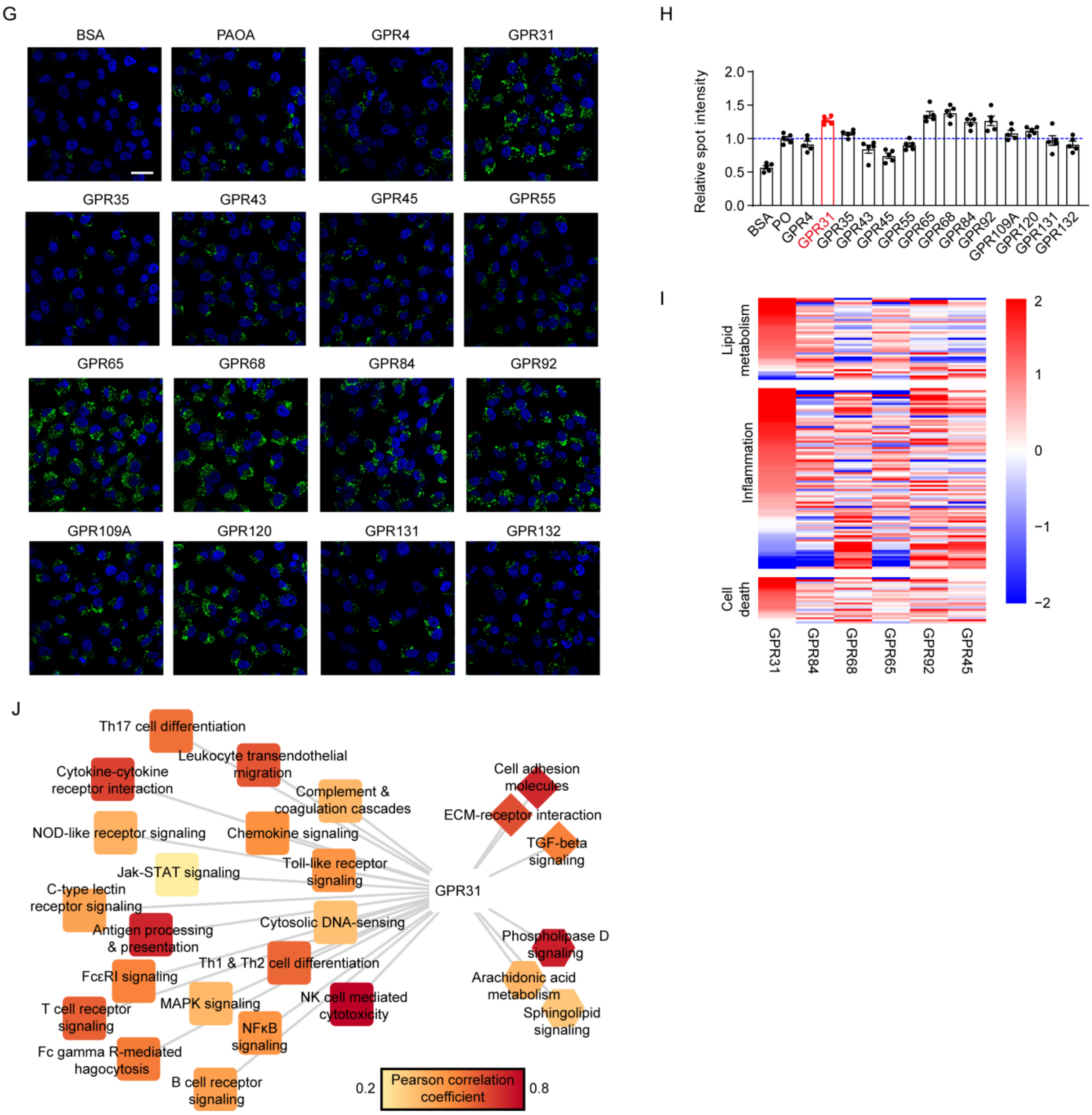

Figure S1. The metabolite-sensing GPCR subfamily was identified as the subfamily with the most significant correlation with MASH

(A) Screening strategy to identify the druggable target protein family in MASH progression.

(B) Druggable targets profile based on Therapeutic Target Database.

(C) Protein families about plasma membrane proteins based on Therapeutic Target Database.

(D) Enrichment frequence of GPCRs subfamilies in MASH transcriptomics.

(E) The enrichment of metabolite sensing GPCRs in samples from clinical individuals and mouse models by GSEA.

(F) The relationships between metabolite sensing GPCRs and the key events in MASH.

(G) High-content screening of tested GPCRs on lipid droplet formation in PAOA challenged hepatocytes. Scale bars, 30 μm. n = 5.

(H) Quantitative results for BODIPY staining of lipid droplets with tested GPCRs overexpression. n = 5.

(I) Heatmap analysis of genes related to inflammation, lipid metabolism and cell death in hepatocytes with overexpression of tested GPCRs.

(J) The relationships between GPR31 and the key events in MASH.

Data are shown as mean ± SEM.

Figure S2

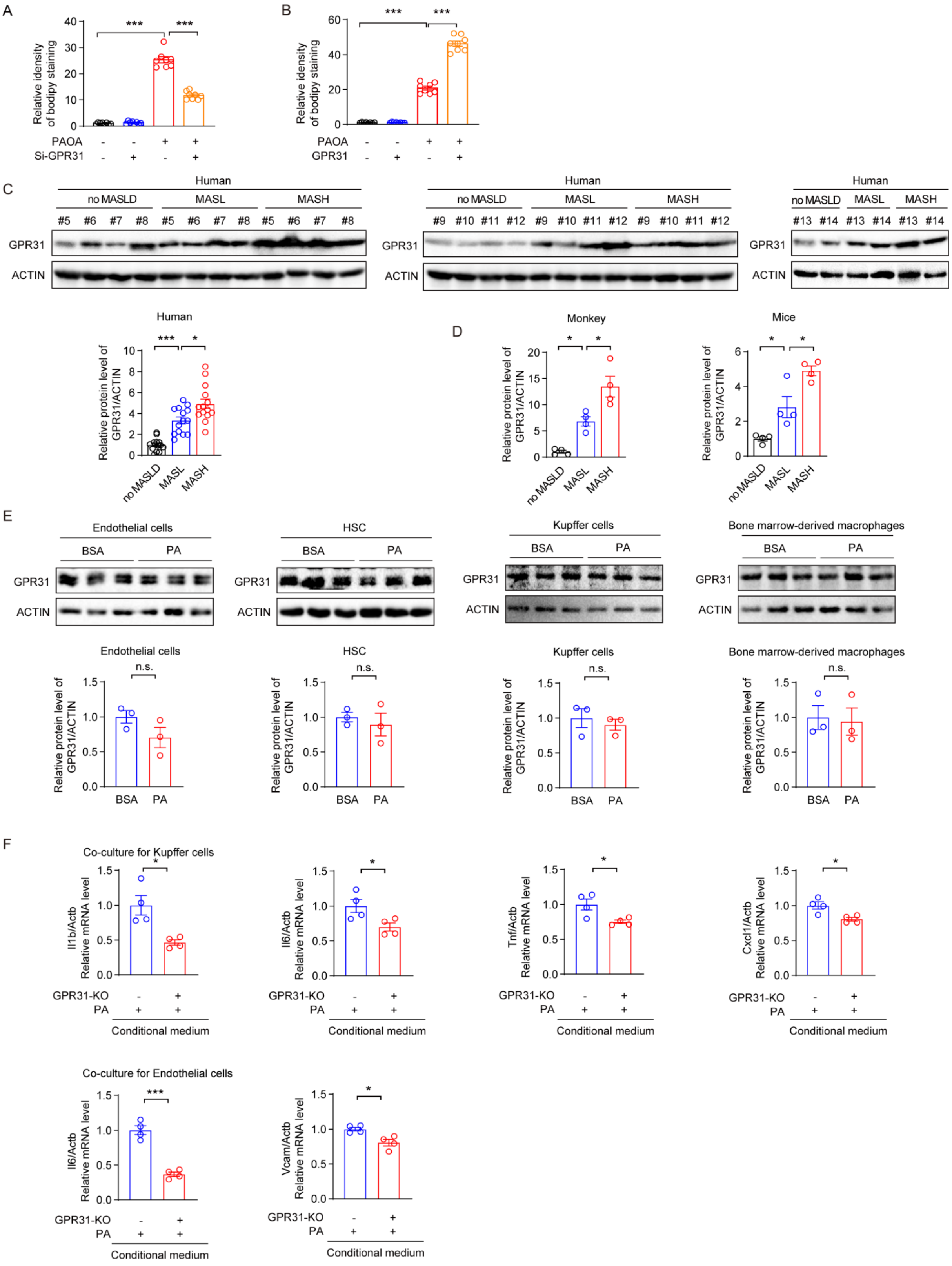

Figure S2 GPR31 function in liver cells

(A) Quantification of BODIPY staining for figure 2B. n = 3 with 8 images quantified.

(B) Quantification of BODIPY staining for figure 2F. n = 3 with 8 images quantified.

(C) Protein levels and quantification of hepatic GPR31 in a cohort of MASH patients and MASLD patients against a control group of age- and gender- matched healthy subjects. n = 14 persons.

(D) Quantification of GPR31 protein levels in Figure 2L and M. n = 4.

(E) Protein levels and quantification of GPR31 in mouse primary hepatic endothelial cells, hepatic stellate cells (HSC), kupffer cells and bone marrow-derived macrophages treated with or without palmitic acid (PA) for 12 h. n = 3 mice.

(F) The WT or GPR31-KO mouse primary hepatocytes were first challenged with BSA or PA for 12 hours followed by incubation with fresh DMEM without PA for an additional 12 hours. The conditional medium was then collected for kupffer cells and endothelial cells incubation for another 12 hours. n = 4 mice.

Data are shown as mean ± SEM. *, $P < 0.05$; **, $P < 0.01$; ***, $P < 0.001$; n.s., no significance, $P > 0.05$.

Figure S3

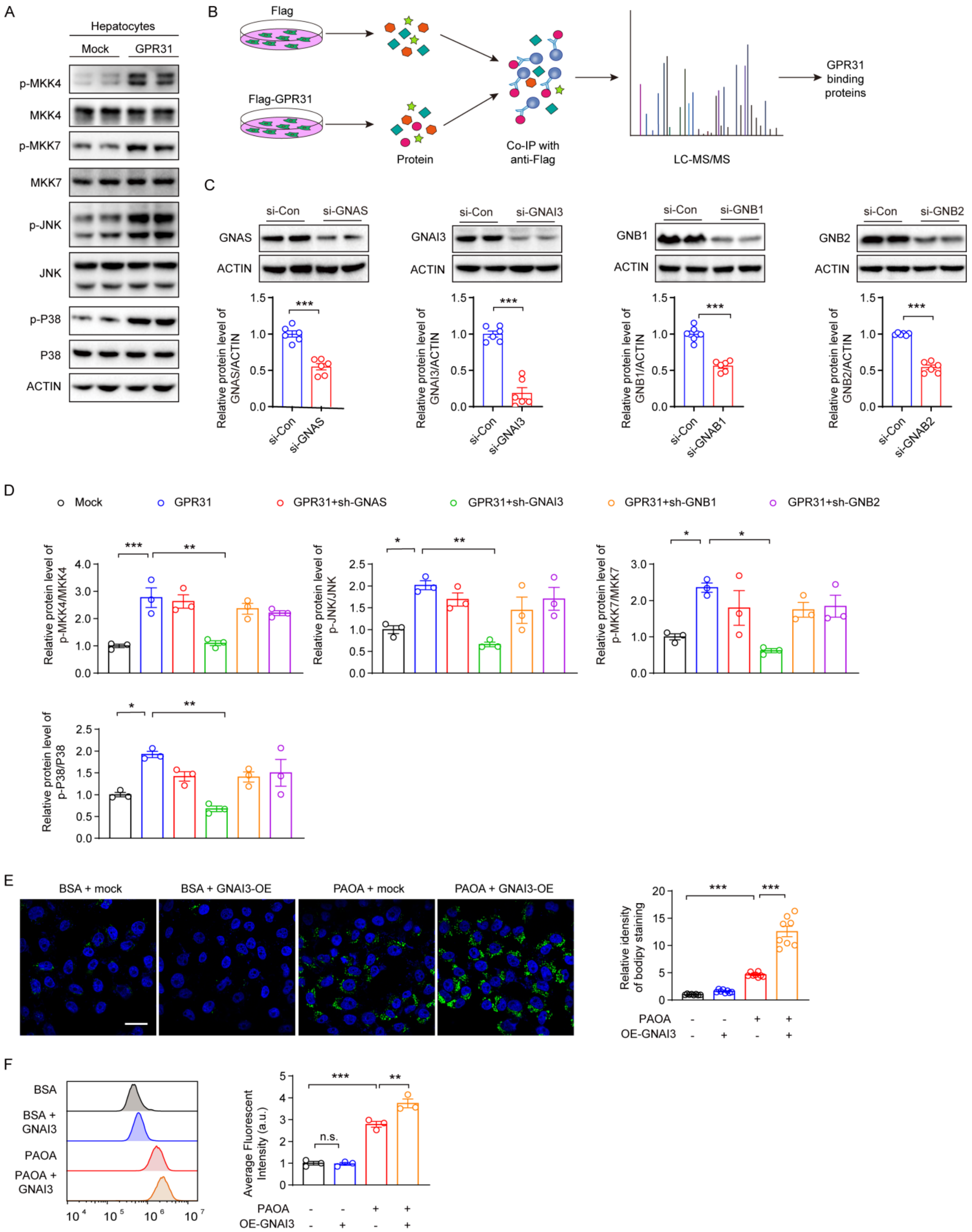
A
Hepatocytes
Mock
GPR31
p-MKK4
MKK4
p-MKK7
MKK7
p-JNK
JNK
p-P38
P38
ACTIN
B
Flag
Flag-GPR31
Protein
Co-IP with anti-Flag
LC-MS/MS
GPR31 binding proteins
C
si-Con
si-GNAS
si-GNAI3
si-GNB1
si-GNB2
GNAS
GNAI3
GNB1
GNB2
ACTIN
Relative protein level of GNAS/ACTIN
Relative protein level of GNAI3/ACTIN
Relative protein level of GNB1/ACTIN
Relative protein level of GNB2/ACTIN
D
Mock
GPR31
GPR31+sh-GNAS
GPR31+sh-GNAI3
GPR31+sh-GNB1
GPR31+sh-GNB2
Relative protein level of p-MKK4/MKK4
Relative protein level of p-JNK/JNK
Relative protein level of p-MKK7/MKK7
Relative protein level of p-P38/P38
E
BSA + mock
BSA + GNAI3-OE
PAOA + mock
PAOA + GNAI3-OE
Relative idensity of bodipy staining
PAOA
OE-GNAI3
F
BSA
BSA + GNAI3
PAOA
PAOA + GNAI3
Average Fluorescent Intensity (a.u.)
n.s.

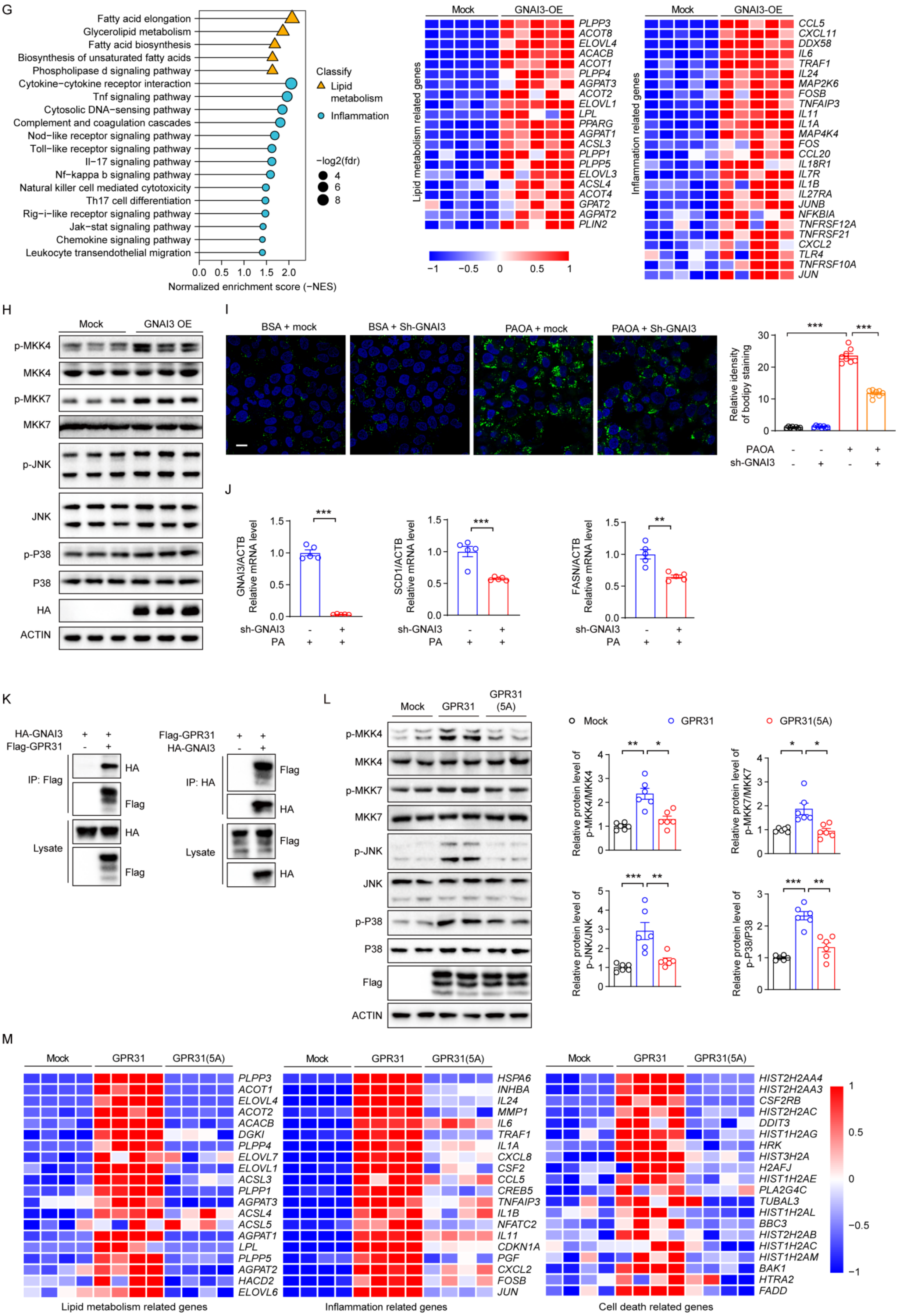
G
Fatty acid elongation
Glycerolipid metabolism
Fatty acid biosynthesis
Biosynthesis of unsaturated fatty acids
Phospholipase d signaling pathway
Cytokine−cytokine receptor interaction
Tnf signaling pathway
Cytosolic DNA−sensing pathway
Complement and coagulation cascades
Nod−like receptor signaling pathway
Toll−like receptor signaling pathway
Il−17 signaling pathway
Nf−kappa b signaling pathway
Natural killer cell mediated cytotoxicity
Th17 cell differentiation
Rig−i−like receptor signaling pathway
Jak−stat signaling pathway
Chemokine signaling pathway
Leukocyte transendothelial migration
Normalized enrichment score (−NES)
Classify
Lipid metabolism
Inflammation
−log2(fdr)
Mock
GNAI3-OE
Lipid metabolism related genes
PLPP3 ACOT8 ELOVL4 ACACB ACOT1 PLPP4 AGPAT3 ACOT2 ELOVL1 LPL PPARG AGPAT1 ACSL3 PLPP1 PLPP5 ELOVL3 ACSL4 ACOT4 GPAT2 AGPAT2 PLIN2
Inflammation related genes
CCL5 CXCL11 DDX58 IL6 TRAF1 IL24 MAP2K6 FOSB TNFAIP3 IL11 IL1A MAP4K4 FOS CCL20 IL18R1 IL7R IL1B IL27RA JUNB NFKBIA TNFRSF12A TNFRSF21 CXCL2 TLR4 TNFRSF10A JUN
H
Mock
GNAI3 OE
p-MKK4
MKK4
p-MKK7
MKK7
p-JNK
JNK
p-P38
P38
HA
ACTIN
I
BSA + mock
BSA + Sh-GNAI3
PAOA + mock
PAOA + Sh-GNAI3
Relative idensity of bodipy staining
PAOA
sh-GNAI3
J
GNAI3/ACTB Relative mRNA level
SCD1/ACTB Relative mRNA level
FASN/ACTB Relative mRNA level
sh-GNAI3
PA
K
HA-GNAI3
Flag-GPR31
IP: Flag
Lysate
Flag-GPR31
HA-GNAI3
IP: HA
HA
Flag
L
Mock
GPR31
GPR31 (5A)
p-MKK4
MKK4
p-MKK7
MKK7
p-JNK
JNK
p-P38
P38
Flag
ACTIN
Mock
GPR31
GPR31(5A)
Relative protein level of p-MKK4/MKK4
Relative protein level of p-MKK7/MKK7
Relative protein level of p-JNK/JNK
Relative protein level of p-P38/P38
M
Mock
GPR31
GPR31(5A)
PLPP3 ACOT1 ELOVL4 ACOT2 ACACB DGKI PLPP4 ELOVL7 ELOVL1 ACSL3 PLPP1 AGPAT3 ACSL4 ACSL5 AGPAT1 LPL PLPP5 AGPAT2 HACD2 ELOVL6
Lipid metabolism related genes
HSPA6 INHBA IL24 MMP1 IL6 TRAF1 IL1A CXCL8 CSF2 CCL5 CREB5 TNFAIP3 IL1B NFATC2 IL11 CDKN1A PGF CXCL2 FOSB JUN
Inflammation related genes
HIST2H2AA4 HIST2H2AA3 CSF2RB HIST2H2AC DDIT3 HIST1H2AG HRK HIST3H2A H2AFJ HIST1H2AE PLA2G4C TUBAL3 HIST1H2AL BBC3 HIST2H2AB HIST1H2AC HIST1H2AM BAK1 HTRA2 FADD
Cell death related genes

Figure S3 GNAI3 exacerbate lipid droplet formation

(A) Effect of GPR31 overexpression on the phosphorylation of key proteins in the MAPK signaling pathway in hepatocytes. n = 3.

(B) Scheme describing the procedure to identify GPR31 interacting proteins by IP-MS in hepatocytes overexpressed with Flag-GPR31.

(C) The knockdown effects of GNAS, GNAI3, GNB1 and GNB2 validated by western blotting. n = 6.

(D) Quantification of protein levels in Figure 3G. n = 3.

(E) BODIPY staining of lipid droplets in hepatocytes with or without GNAI3 overexpression. Scale bars, 30μm. n = 3 with 8 images quantified.

(F) BODIPY staining for quantification by flow cytometry in control and GNAI3 overexpressed hepatocytes. n = 3.

(G) Pathway and heatmap analysis of GNAI3 overexpressed hepatocytes. n = 5.

(H) Effect of GNAI3 overexpression on the phosphorylation of key proteins in the MAPK signaling pathway in hepatocytes. n = 3.

(I) BODIPY staining of lipid droplets in hepatocytes with or without GNAI3 knockdown. Scale bars, 30μm. n = 3 with 8 images quantified.

(J) Effect of GNAI3 knockdown on SCD1 and FASN mRNA level. n = 5.

(K) Coimmunoprecipitation (Co-IP) assays were performed to examine the interaction of GPR31 and GNAI3. n = 3.

(L) Effect of GPR31 or its docking mutant GPR31(5A) overexpression on the phosphorylation of key proteins in the MAPK signaling pathway in hepatocytes. n = 6.

(M) Transcriptomic profiling of hepatocytes transfected with GPR31 or GPR31(5A). Differentially expressed genes in pathways of lipid metabolism, inflammatory response and cell death are presented. n = 4.

Data are shown as mean ± SEM. *, $P < 0.05$; **, $P < 0.01$; ***, $P < 0.001$; n.s., no significance, $P > 0.05$.

Figure S4

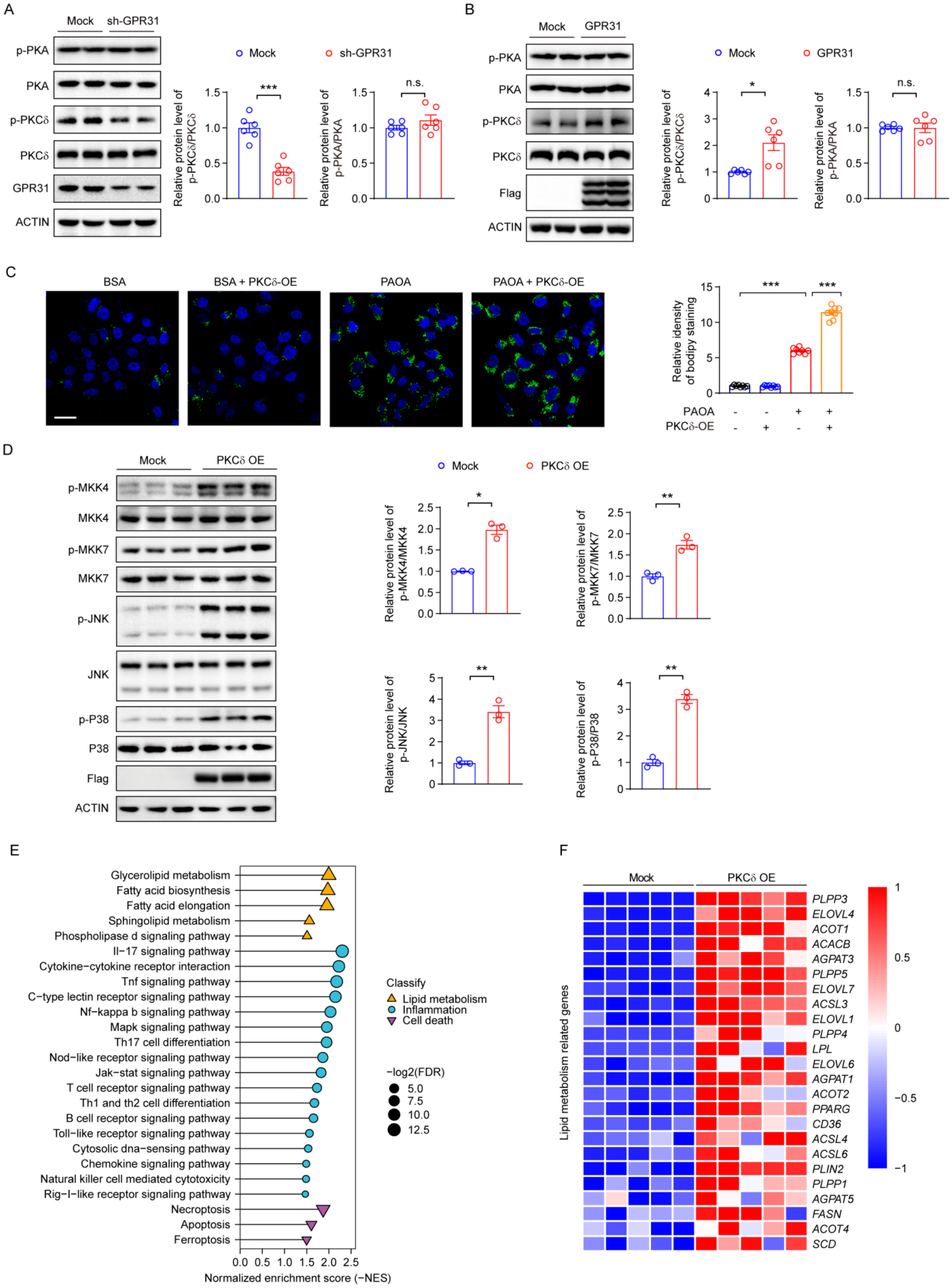
A
Mock
sh-GPR31
p-PKA
PKA
p-PKCδ
PKCδ
GPR31
ACTIN
Relative protein level of p-PKCδ/PKCδ
Relative protein level of p-PKA/PKA
***
n.s.
B
Mock
GPR31
Flag
*
C
BSA
BSA + PKCδ-OE
PAOA
PAOA + PKCδ-OE
Relative idensity of bodipy staining
PAOA
PKCδ-OE
D
PKCδ OE
p-MKK4
MKK4
p-MKK7
MKK7
p-JNK
JNK
p-P38
P38
Relative protein level of p-MKK4/MKK4
Relative protein level of p-MKK7/MKK7
Relative protein level of p-JNK/JNK
Relative protein level of p-P38/P38
**
E
Glycerolipid metabolism
Fatty acid biosynthesis
Fatty acid elongation
Sphingolipid metabolism
Phospholipase d signaling pathway
Il−17 signaling pathway
Cytokine−cytokine receptor interaction
Tnf signaling pathway
C−type lectin receptor signaling pathway
Nf−kappa b signaling pathway
Mapk signaling pathway
Th17 cell differentiation
Nod−like receptor signaling pathway
Jak−stat signaling pathway
T cell receptor signaling pathway
Th1 and th2 cell differentiation
B cell receptor signaling pathway
Toll−like receptor signaling pathway
Cytosolic dna−sensing pathway
Chemokine signaling pathway
Natural killer cell mediated cytotoxicity
Rig−I−like receptor signaling pathway
Necroptosis
Apoptosis
Ferroptosis
Normalized enrichment score (−NES)
Classify
Lipid metabolism
Inflammation
Cell death
−log2(FDR)
5.0
7.5
10.0
12.5
F
Lipid metabolism related genes
PLPP3
ELOVL4
ACOT1
ACACB
AGPAT3
PLPP5
ELOVL7
ACSL3
ELOVL1
PLPP4
LPL
ELOVL6
AGPAT1
ACOT2
PPARG
CD36
ACSL4
ACSL6
PLIN2
PLPP1
AGPAT5
FASN
ACOT4
SCD

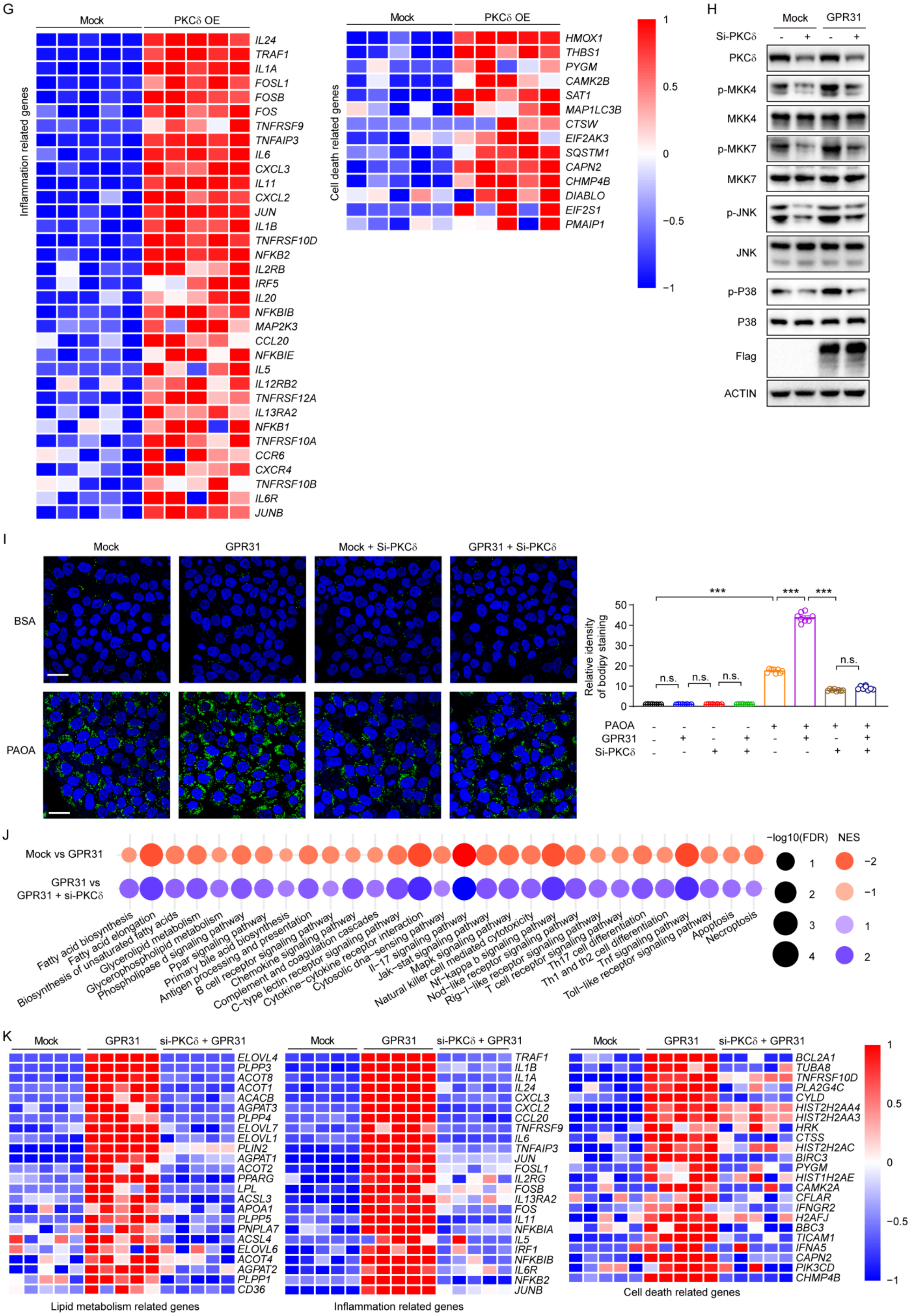

G
Mock
PKCδ OE
Inflammation related genes
IL24
TRAF1
IL1A
FOSL1
FOSB
FOS
TNFRSF9
TNFAIP3
IL6
CXCL3
IL11
CXCL2
JUN
IL1B
TNFRSF10D
NFKB2
IL2RB
IRF5
IL20
NFKBIB
MAP2K3
CCL20
NFKBIE
IL5
IL12RB2
TNFRSF12A
IL13RA2
NFKB1
TNFRSF10A
CCR6
CXCR4
TNFRSF10B
IL6R
JUNB
Cell death related genes
HMOX1
THBS1
PYGM
CAMK2B
SAT1
MAP1LC3B
CTSW
EIF2AK3
SQSTM1
CAPN2
CHMP4B
DIABLO
EIF2S1
PMAIP1
1
0.5
0
−0.5
−1
H
Mock
GPR31
Si-PKCδ
PKCδ
p-MKK4
MKK4
p-MKK7
MKK7
p-JNK
JNK
p-P38
P38
Flag
ACTIN
I
Mock
GPR31
Mock + Si-PKCδ
GPR31 + Si-PKCδ
BSA
PAOA
Relative idensity of bodipy staining
***
n.s.
PAOA
GPR31
Si-PKCδ
J
Mock vs GPR31
GPR31 vs GPR31 + si-PKCδ
−log10(FDR)
NES
Fatty acid biosynthesis
Fatty acid elongation
Biosynthesis of unsaturated fatty acids
Glycerolipid metabolism
Glycerophospholipid metabolism
Phospholipase d signaling pathway
Ppar signaling pathway
Primary bile acid biosynthesis
Antigen processing and presentation
B cell receptor signaling pathway
Chemokine signaling pathway
Complement and coagulation cascades
C-type lectin receptor signaling pathway
Cytokine−cytokine receptor interaction
Cytosolic dna−sensing pathway
Il−17 signaling pathway
Jak−stat signaling pathway
Mapk signaling pathway
Natural killer cell mediated cytotoxicity
Nf−kappa b signaling pathway
Nod−like receptor signaling pathway
Rig−I−like receptor signaling pathway
T cell receptor signaling pathway
Th17 cell differentiation
Th1 and th2 cell differentiation
Tnf signaling pathway
Toll−like receptor signaling pathway
Apoptosis
Necroptosis
K
Mock
GPR31
si-PKCδ + GPR31
ELOVL4
PLPP3
ACOT8
ACOT1
ACACB
AGPAT3
PLPP4
ELOVL7
ELOVL1
PLIN2
AGPAT1
ACOT2
PPARG
LPL
ACSL3
APOA1
PLPP5
PNPLA7
ACSL4
ELOVL6
ACOT4
AGPAT2
PLPP1
CD36
Lipid metabolism related genes
TRAF1
IL1B
IL1A
IL24
CXCL3
CXCL2
CCL20
TNFRSF9
IL6
TNFAIP3
JUN
FOSL1
IL2RG
FOSB
IL13RA2
FOS
IL11
NFKBIA
IL5
IRF1
NFKBIB
IL6R
NFKB2
JUNB
Inflammation related genes
BCL2A1
TUBA8
TNFRSF10D
PLA2G4C
CYLD
HIST2H2AA4
HIST2H2AA3
HRK
CTSS
HIST2H2AC
BIRC3
PYGM
HIST1H2AE
CAMK2A
CFLAR
IFNGR2
H2AFJ
BBC3
TICAM1
IFNA5
CAPN2
PIK3CD
CHMP4B
Cell death related genes

126

127

Figure S4. GPR31 aggravates MAPK-mediated MASH in a PKCδ dependent manner

(A) Effect of GPR31 knockdown on the phosphorylation of PKA and PKCδ in hepatocytes. n = 6.

(B) Effect of GPR31 overexpression on the phosphorylation of PKA and PKCδ in hepatocytes. n = 6.

(C) BODIPY staining of lipid droplets in hepatocytes after BSA or PAOA stimulation, with or without PKCδ overexpression. Scale bars, 30μm. n = 3 with 8 images quantified.

(D) Effect of PKCδ overexpression on the phosphorylation of key proteins in the MAPK signaling pathway in hepatocytes. n = 3.

(E) Enriched pathway analysis of hepatocytes with or without PKCδ overexpression. n = 5.

(F) Heat maps showing different expressed genes in pathways of lipid metabolism in hepatocytes with or without PKCδ overexpression. n = 5.

(G) Heat maps showing different expressed genes in pathways of inflammatory response and cell death in hepatocytes with or without PKCδ overexpression. n = 5.

(H) Western blotting of the phosphorylation of key proteins in the MAPK signaling pathway in hepatocytes with or without GPR31 overexpression and PKCδ depletion. n = 3.

(I) BODIPY staining of lipid droplets in hepatocytes with or without GPR31 overexpression and PKCδ depletion. Scale bars, 30μm. n = 3 with 8 images quantified.

(J) Enriched pathway analysis of hepatocytes with or without GPR31 overexpression and PKCδ depletion. n = 5.

(K) Heat maps showing different expressed genes in hepatocytes with or without GPR31 overexpression and PKCδ depletion. n = 5.

Data are shown as mean ± SEM. *, $P < 0.05$; **, $P < 0.01$; ***, $P < 0.001$; n.s., no significance, $P > 0.05$.

Figure S5

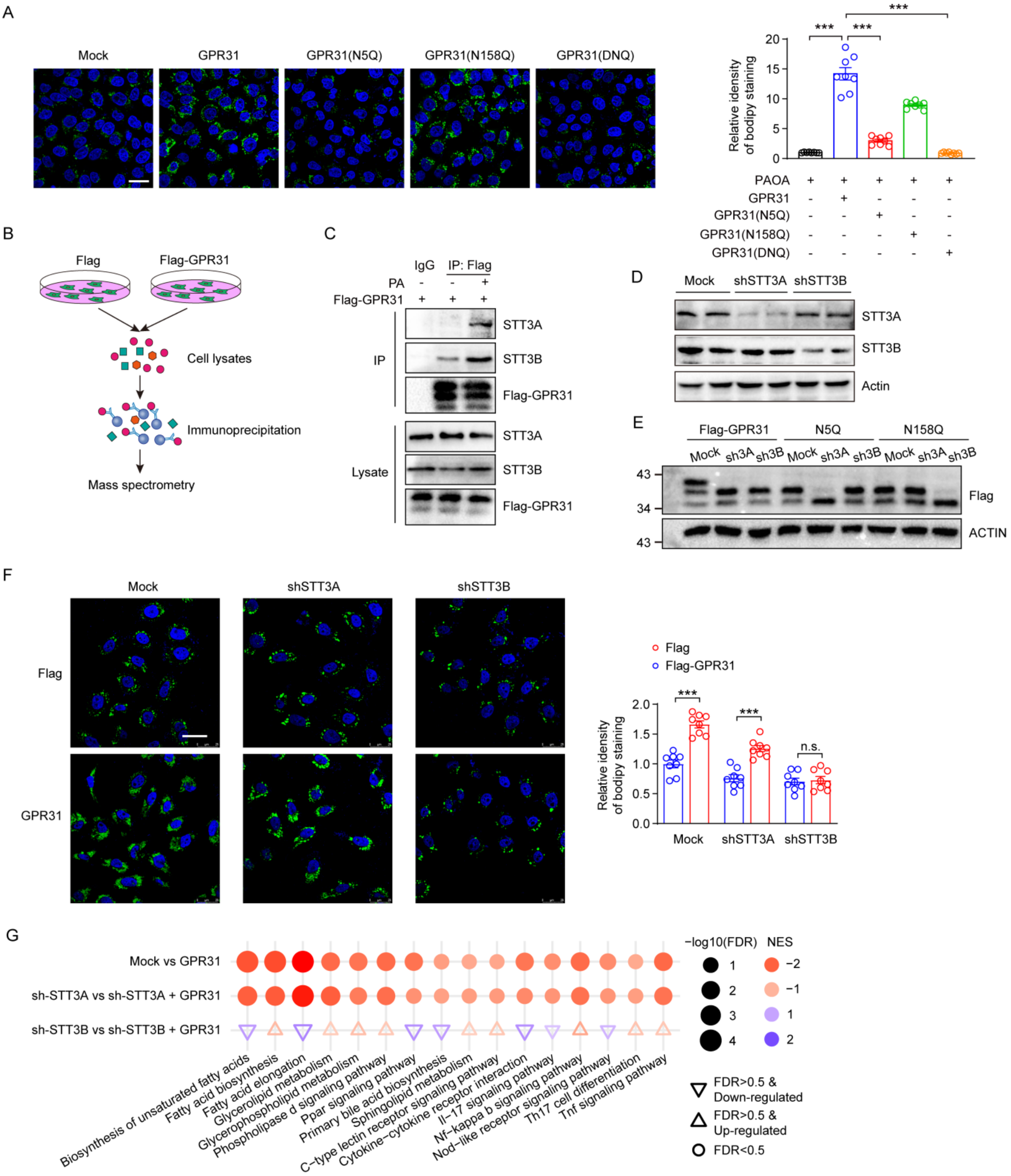

Figure S5. Glycosylation of GPR31 at Asn5 is mediated by STT3B and determines its cytomembrane localization

(A) BODIPY staining of lipid droplets in GPR31-wild type and its nonglycosylatable mutants overexpressed hepatocytes. Scale bars, 30μm. n = 3 with 8 images quantified.

(B) Schematic of the interaction proteomics assayed by cells transfected with Flag or Flag-GPR31.

(C) The interaction of GPR31 and two conventional oligosaccharyltransferases STT3A and STT3B. n = 3.

(D) The protein levels of STT3A and STT3B after STT3A or STT3B knockdown. n = 3.

(E) The immunoblot of GPR31 in cells transfected with GPR31-wild type or its nonglycosylatable mutants after STT3A or STT3B knockdown. n = 3.

(F) BODIPY staining and quantitative results of lipid droplets in Flag or Flag-GPR31 overexpressed hepatocytes after STT3A or STT3B knockdown. Scale bars, 30μm. n = 3.

(G) Enriched pathway analysis of Flag or Flag-GPR31 overexpressed hepatocytes after STT3A or STT3B knockdown.

Data are shown as mean ± SEM. ***, $P < 0.001$; n.s., no significance, $P > 0.05$.

Figure S6

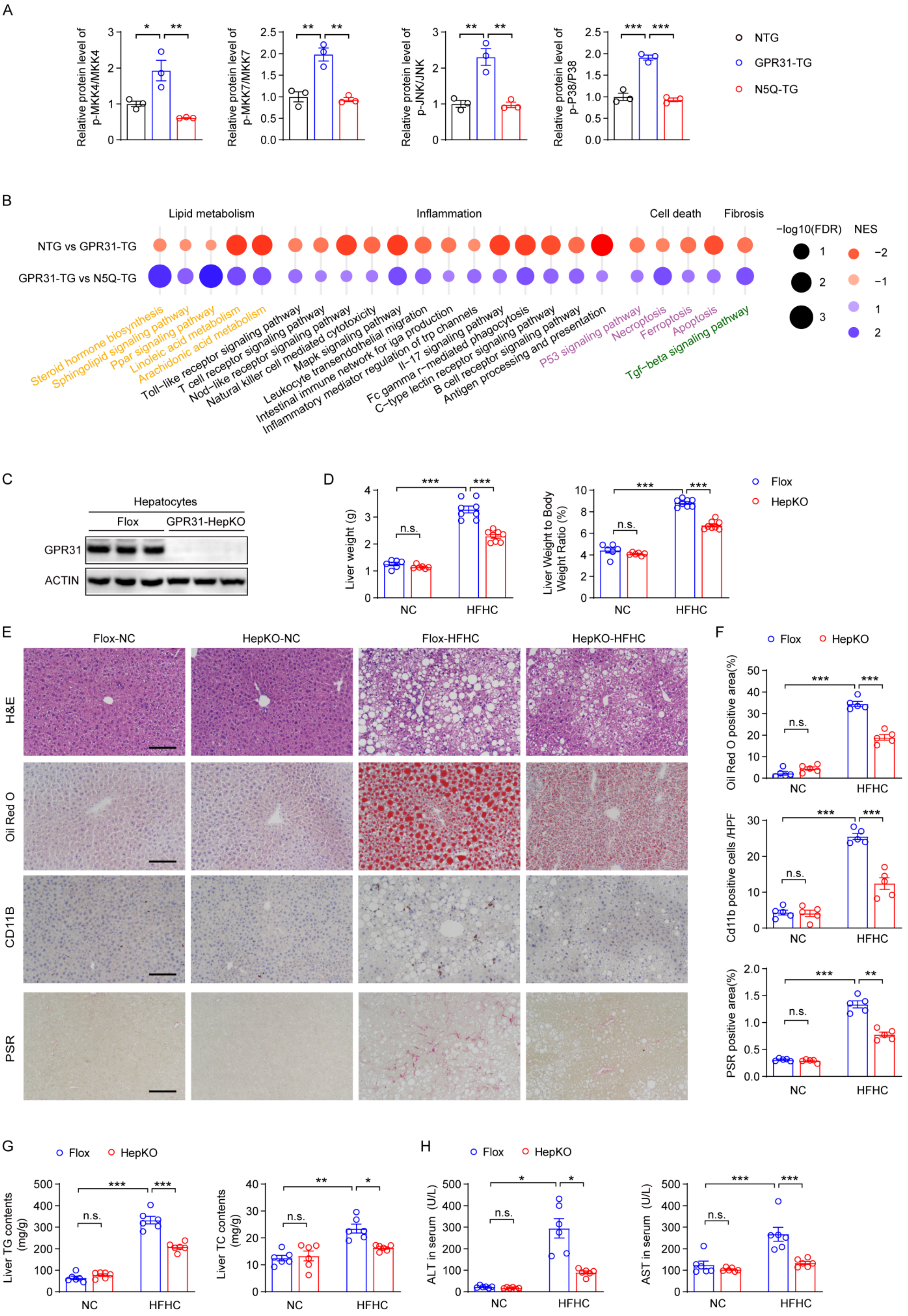
A
Relative protein level of p-MKK4/MKK4
Relative protein level of p-MKK7/MKK7
Relative protein level of p-JNK/JNK
Relative protein level of p-P38/P38
NTG
GPR31-TG
N5Q-TG
B
Lipid metabolism
Inflammation
Cell death
Fibrosis
NTG vs GPR31-TG
GPR31-TG vs N5Q-TG
−log10(FDR)
NES
Steroid hormone biosynthesis
Sphingolipid signaling pathway
Ppar signaling pathway
Linoleic acid metabolism
Arachidonic acid metabolism
Toll-like receptor signaling pathway
T cell receptor signaling pathway
Nod-like receptor signaling pathway
Natural killer cell mediated cytotoxicity
Mapk signaling pathway
Leukocyte transendothelial migration
Intestinal immune network for iga production
Inflammatory mediator regulation of trp channels
Il-17 signaling pathway
Fc gamma r-mediated phagocytosis
C-type lectin receptor signaling pathway
B cell receptor signaling pathway
Antigen processing and presentation
P53 signaling pathway
Necroptosis
Ferroptosis
Apoptosis
Tgf-beta signaling pathway
C
Hepatocytes
Flox
GPR31-HepKO
GPR31
ACTIN
D
Liver weight (g)
Liver Weight to Body Weight Ratio (%)
NC
HFHC
Flox
HepKO
E
Flox-NC
HepKO-NC
Flox-HFHC
HepKO-HFHC
H&E
Oil Red O
CD11B
PSR
F
Oil Red O positive area(%)
Cd11b positive cells /HPF
PSR positive area(%)
G
Liver TG contents (mg/g)
Liver TC contents (mg/g)
H
ALT in serum (U/L)
AST in serum (U/L)

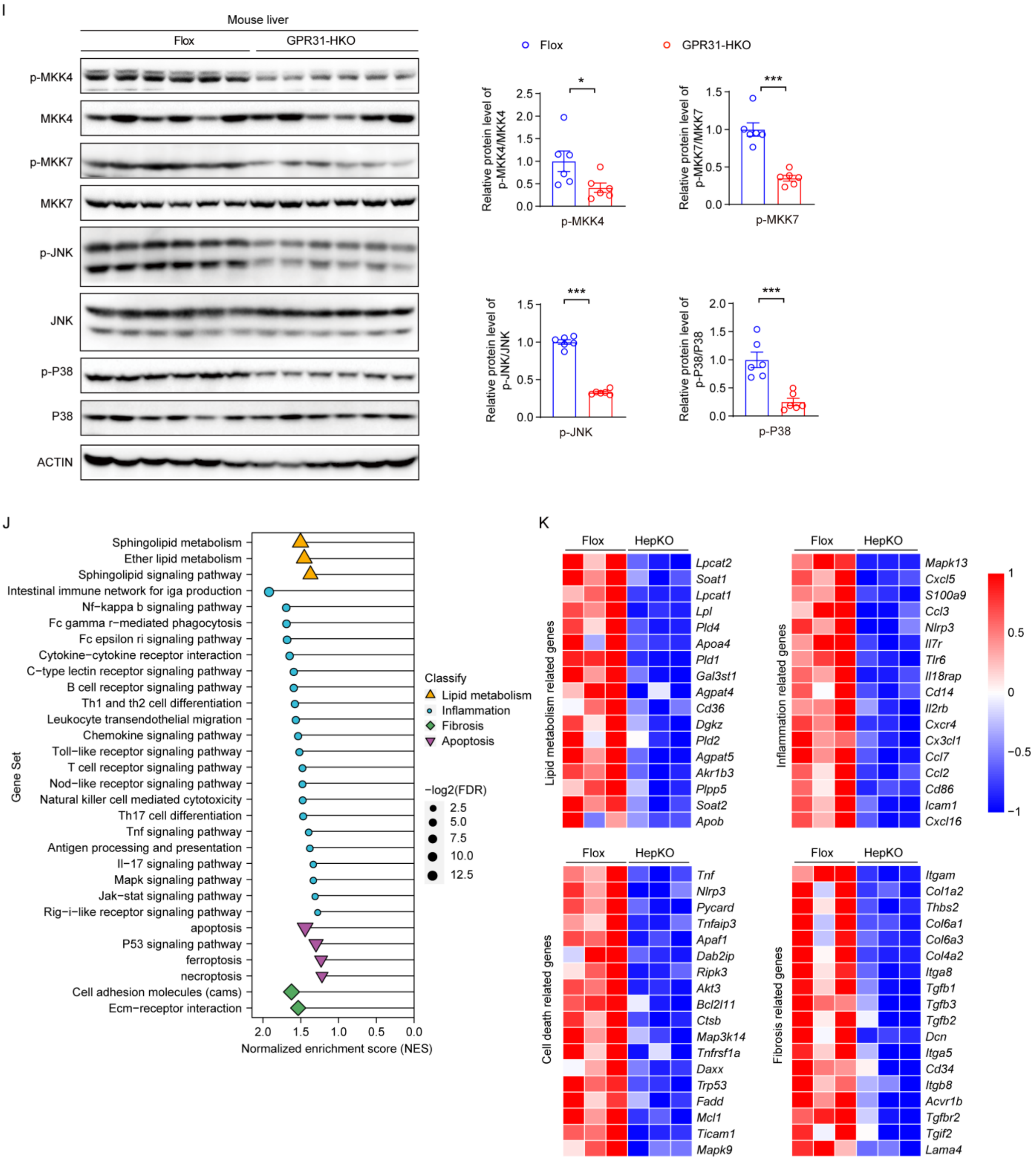

Figure S6 Targeted deletion of GPR31 in hepatocytes protects against MASH in mice

(A) Quantification of the protein levels in Figure 5F. n = 3 mice.

(B) Dot blot representing pairwise GSEA comparison of RNA-seq data obtained from GPR31-WT and GPR31-N5Q transgenic mouse livers. n = 3 mice.

(C) Validation of GPR31-flox and GPR31-HepKO mutant mice by western blot analysis. n = 3 mice.

(D) Liver weight and liver weight/body weight ratio in Flox (GPR31$^{flox/flox}$) and GPR31-HKO mice. n = 8 mice.

(E) H&E, oil red O, CD11B and picosirus red (PSR) staining of the liver sections in Flox and

GPR31-HKO mice. n = 5 mice. Scale bars, 100 mm.

(F) Quantitative results of oil red O, CD11B and picosirus red (PSR) staining in (D). n = 5 mice.

(G) Hepatic TG and TC contents of the mice in the indicated group. n = 6 mice.

(H) Serum ALT and AST levels of the mice in the indicated group. n = 6 mice.

(I) Effect of GPR31-HKO on the phosphorylation of key proteins in the MAPK signaling pathway in mouse liver. n = 6 mice.

(J) Enriched pathway analysis of liver tissues from HFHC-16W Flox and GPR31-HKO mice. n = 3 mice.

(K) Heat maps showing different expressed genes in pathways of lipid metabolism, inflammatory response, cell death and fibrosis in liver tissues from HFHC-16W Flox and GPR31-HKO mice. n = 3 mice.

Data are shown as mean ± SEM. *, $P < 0.05$; **, $P < 0.01$; ***, $P < 0.001$; n.s., no significance, $P > 0.05$.

Figure S7

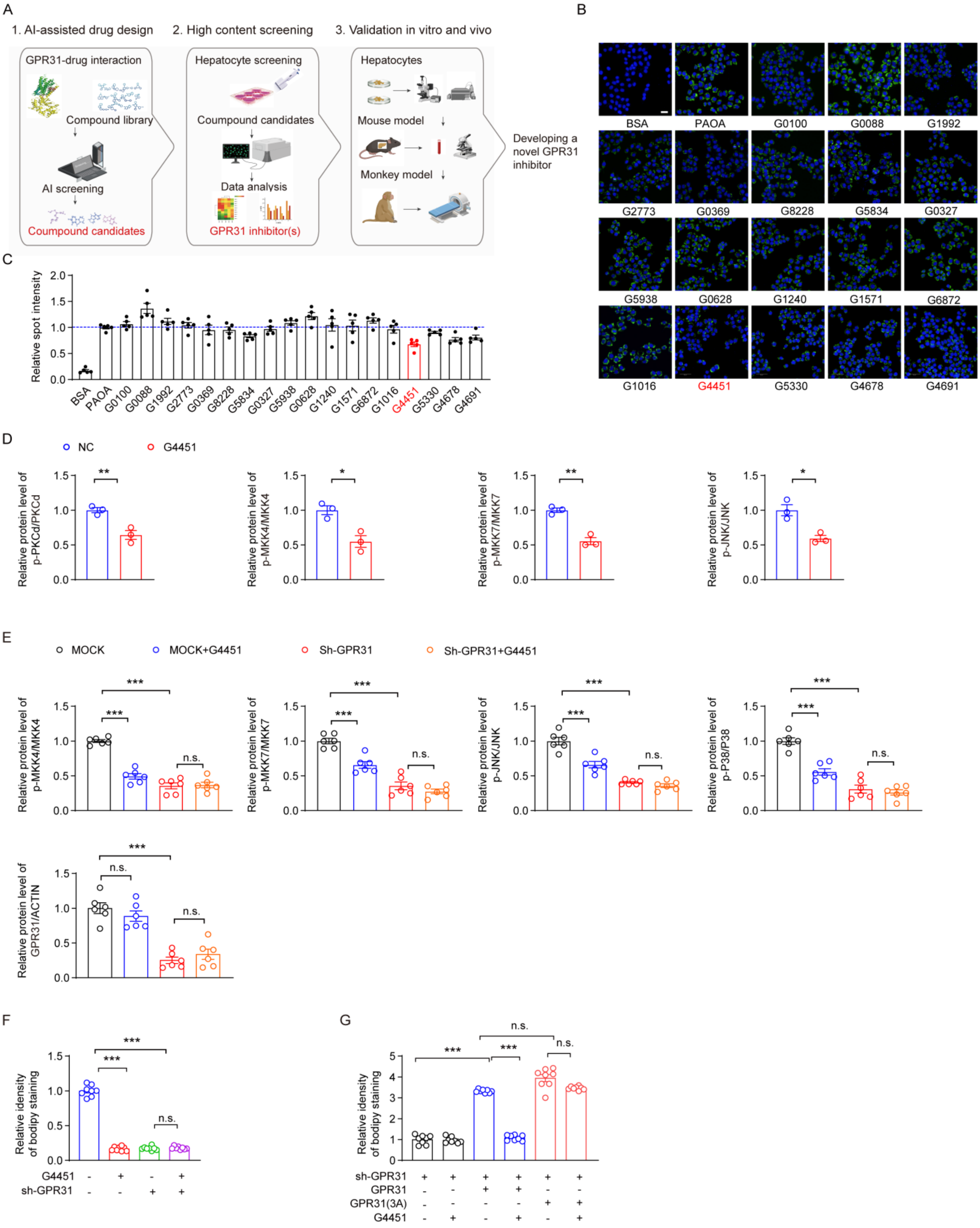

A
1. AI-assisted drug design
2. High content screening
3. Validation in vitro and vivo
GPR31-drug interaction
Compound library
AI screening
Coumpound candidates
Hepatocyte screening
Coumpound candidates
Data analysis
GPR31 inhibitor(s)
Hepatocytes
Mouse model
Monkey model
Developing a novel GPR31 inhibitor
B
BSA
PAOA
G0100
G0088
G1992
G2773
G0369
G8228
G5834
G0327
G5938
G0628
G1240
G1571
G6872
G1016
G4451
G5330
G4678
G4691
C
Relative spot intensity
D
NC
G4451
Relative protein level of p-PKCd/PKCd
Relative protein level of p-MKK4/MKK4
Relative protein level of p-MKK7/MKK7
Relative protein level of p-JNK/JNK
E
MOCK
MOCK+G4451
Sh-GPR31
Sh-GPR31+G4451
Relative protein level of p-MKK4/MKK4
Relative protein level of p-MKK7/MKK7
Relative protein level of p-JNK/JNK
Relative protein level of p-P38/P38
Relative protein level of GPR31/ACTIN
F
Relative idensity of bodipy staining
G4451
sh-GPR31
G
Relative idensity of bodipy staining
sh-GPR31
GPR31
GPR31(3A)
G4451

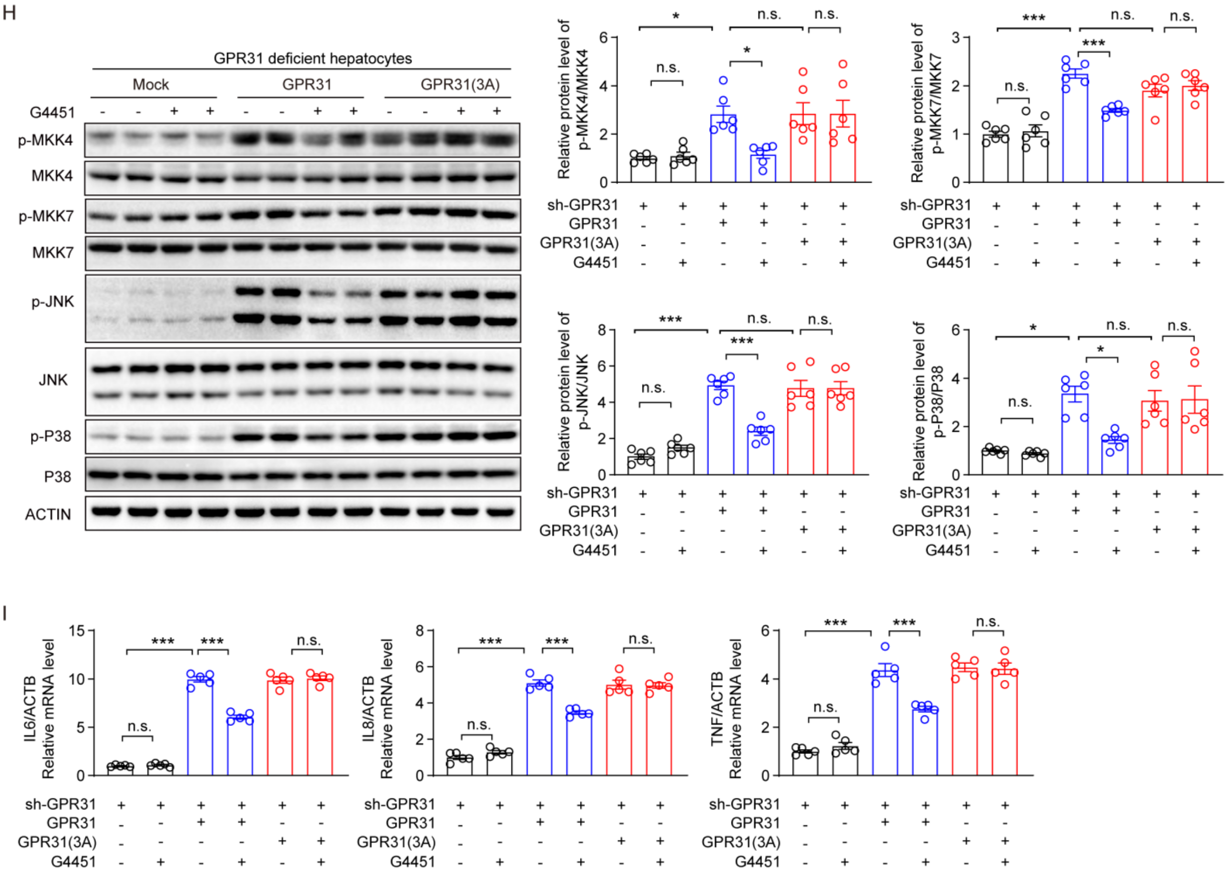

Figure S7 G4451 was developed as a new anti-MASH candidate via blockade of the GPR31-Gαi3 interaction

(A) Design and screening of small-molecule inhibitors of GPR31 using homology remodeling based on GPR31.

(B) High-content screening of potential inhibitors of GPR31 by BODIPY staining of lipid droplets in hepatocytes. Scale bars, 30μm. n = 5.

(C) Quantitative results of lipid droplets in (B). n = 5.

(D) Quantification of the protein levels in Figure 6B. n = 3.

(E) Quantification of the protein levels in Figure 6D. n = 6.

(F) Quantification of BODIPY staining for Figure 6E. n = 3 with 8 images quantified.

(G) Quantification of BODIPY staining for Figure 6L. n = 3 with 8 images quantified.

(H) Western blotting showing the phosphorylation of key proteins in the MAPK signaling pathway in GPR31 deficient hepatocytes with GPR31 overexpression, GPR31(3A) overexpression, or G4451 treatment. n = 6.

(I) mRNA levels of inflammation factors in GPR31 deficient hepatocytes with GPR31 overexpression, GPR31(3A) overexpression, or G4451 treatment. n = 5.

Data are shown as mean ± SEM. *, $P < 0.05$; **, $P < 0.01$; ***, $P < 0.001$; n.s., no significance, $P > 0.05$.

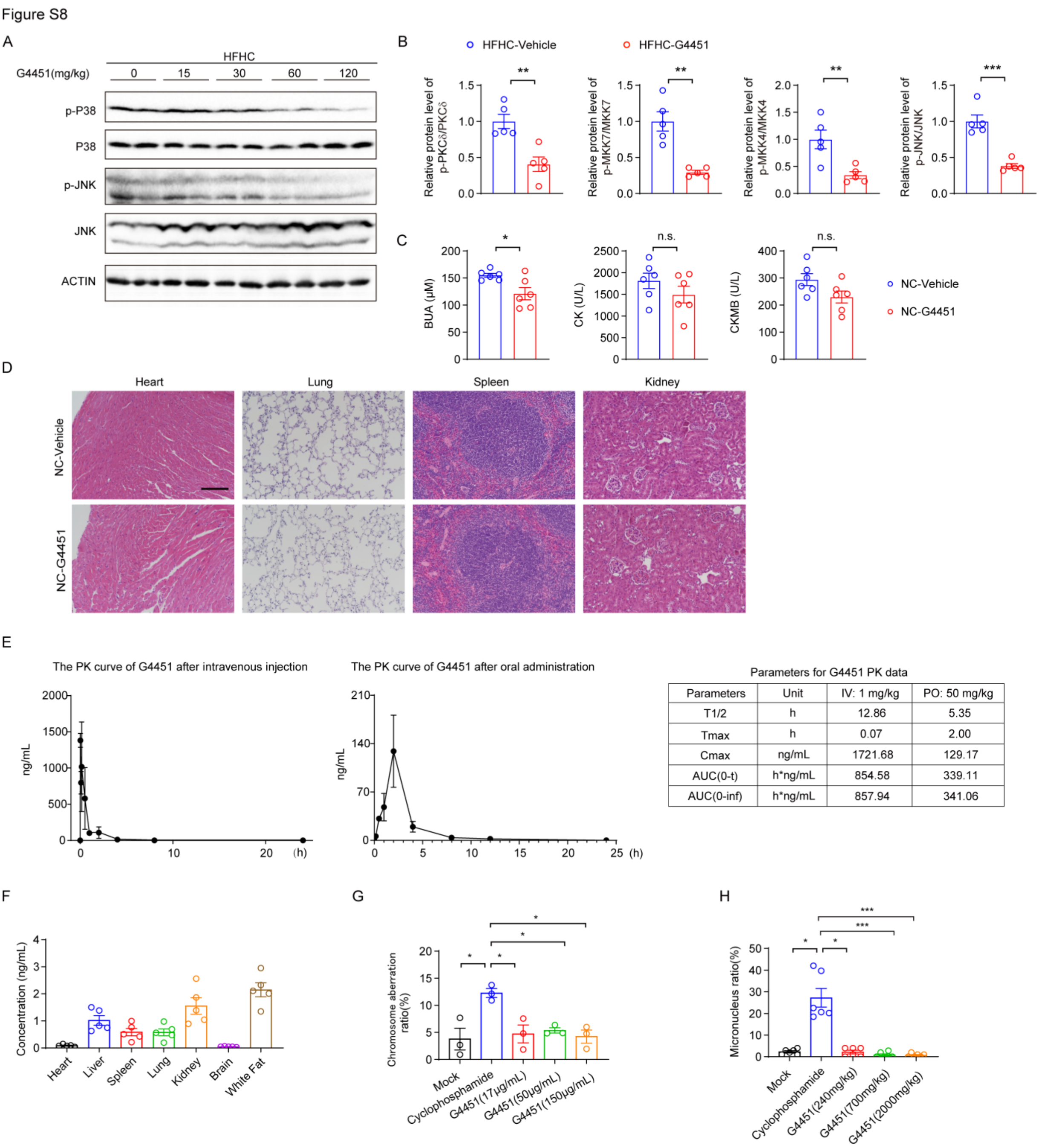

Parameters	Unit	IV: 1 mg/kg	PO: 50 mg/kg
T1/2	h	12.86	5.35
Tmax	h	0.07	2.00
Cmax	ng/mL	1721.68	129.17
AUC(0-t)	h*ng/mL	854.58	339.11
AUC(0-inf)	h*ng/mL	857.94	341.06

Figure S8 G4451 has no side effect in mice

(A) Western blotting showing the phosphorylation of key proteins in the MAPK signaling pathway by G4451 treatment.

(B) Quantification of the protein levels in Figure 7D. n = 5 mice.

(C) Serum BUA, CK and CKMB contents in mice treated with vehicle or G4451. n = 6 mice.

(D) Representative images of H&E staining on mouse heart, lung, spleen and kidney sections in G4451- and vehicle-treated mice fed a NC diet. n = 6 mice. Scale bars, 100 μm.

(E) The PK data of G4451 in Wistar rats. n = 3 rats.

(F) The tissue accumulation of G4451 by oral administration. n = 5 mice.

(G) The chromosomal aberration assay of G4451 in CHO cells. n = 3.

(H) The bone marrow micronucleus assay of G4451 in mouse. n = 6.

Data are shown as mean ± SEM. *, $P < 0.05$; ***, $P < 0.001$;n.s., no significance, $P > 0.05$.

Figure S9

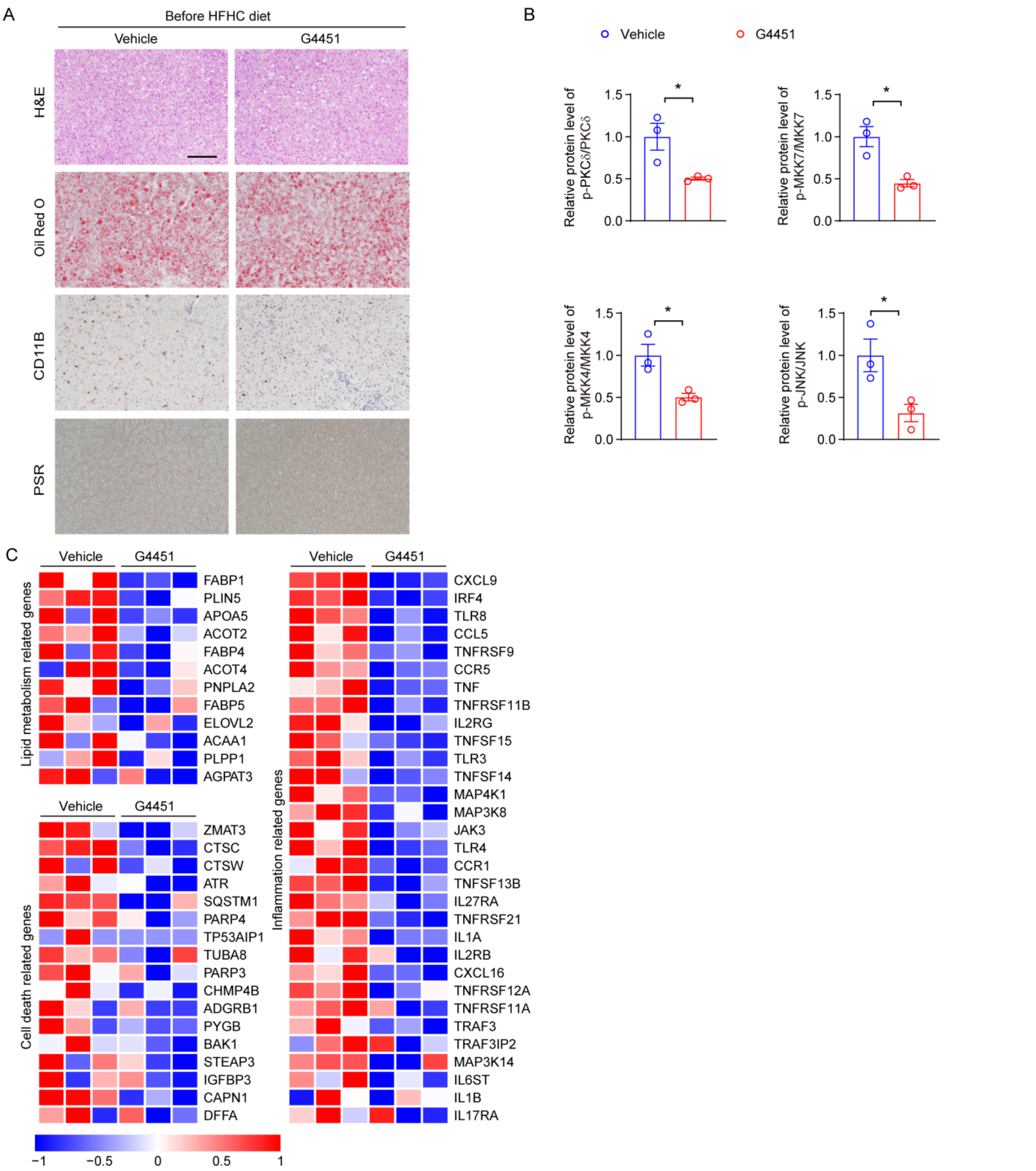

Figure S9 G4451 effectively blocks MASH in monkeys

(A) Representative images of H&E, Oil Red O, CD11B and picosirus red (PSR) staining on monkey liver sections before HFHC diet. Scale bars, 100 μm. n = 4 monkeys.

(B) Quantification of the protein levels in Figure 8E. n = 3 monkeys.

(C) Heat maps showing different expressed genes in pathways of lipid metabolism, inflammatory response and fibrosis in liver tissues from HFHC monkeys treated with vehicle or G4451. n = 3 monkeys.

Data are shown as mean ± SEM. *, $P < 0.05$.

Figure S10

A

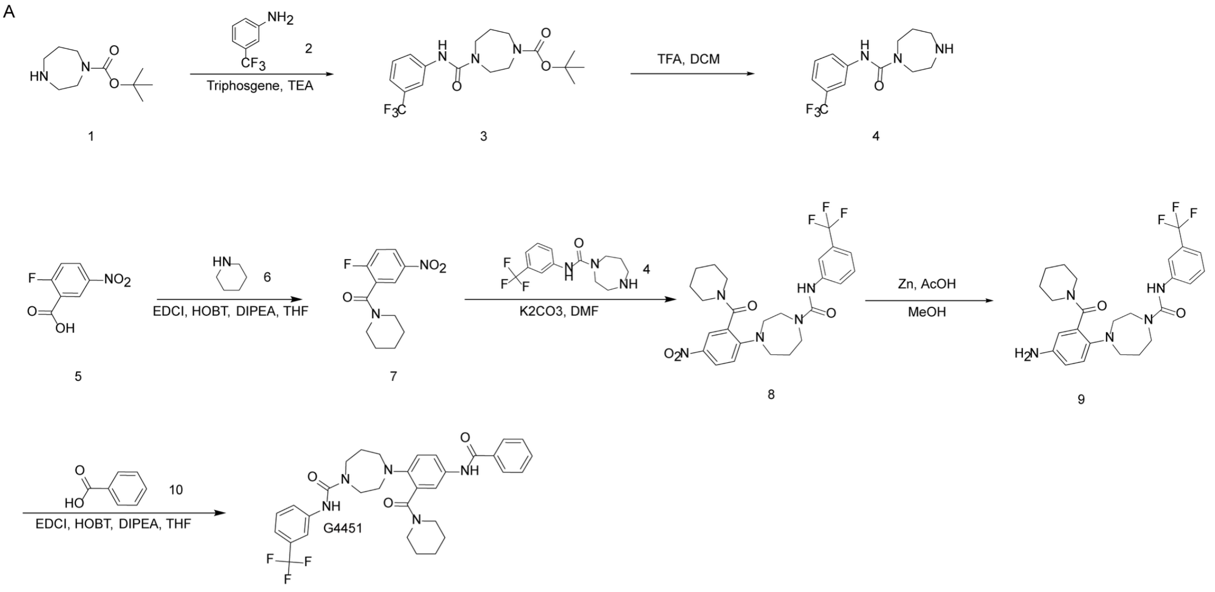

B

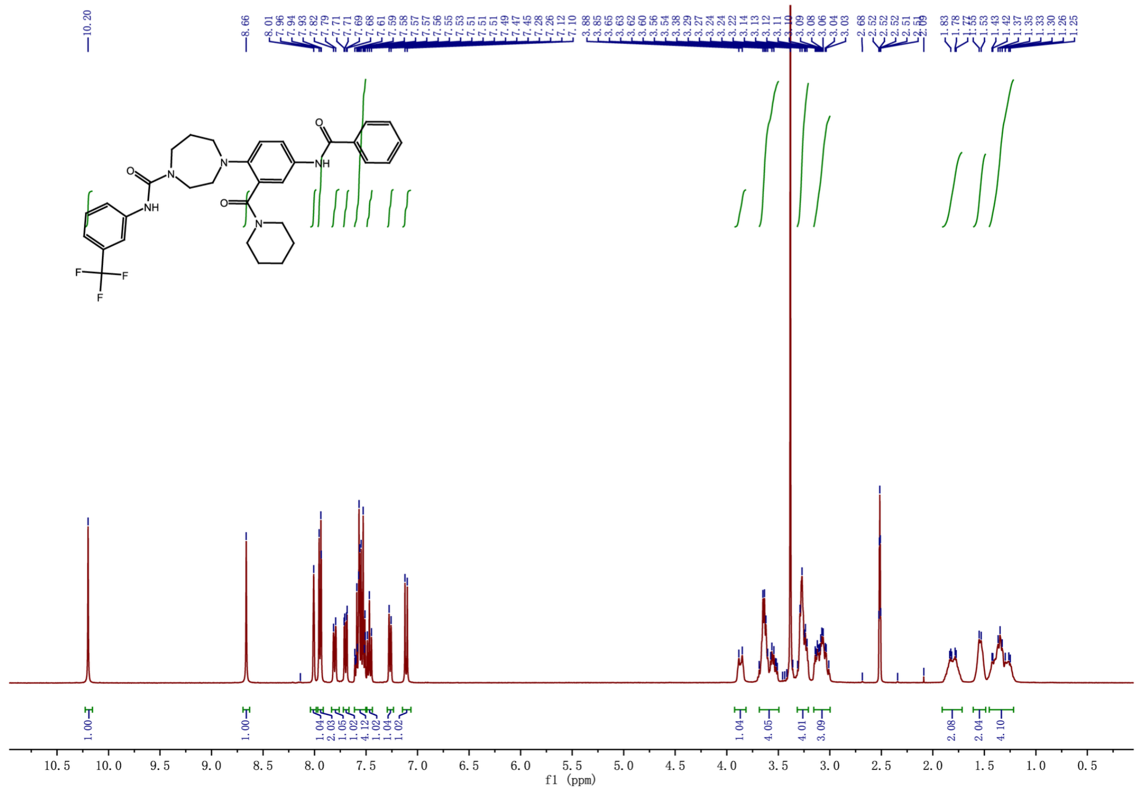

Figure S10 The structure information and synthesis procedure of G4451

(A) The synthetic procedure of G4451. 1-Boc-hexahydro-1,4-diazepine and 3-Aminobenzotrifluoride reacted by condensation reaction to form compound 3, then deprotected by TFA to give intermediate 4. Amide 7 was obtained from 2-Fluoro-5-nitrobenzoic acid and piperidine, then reacted with intermediate 4 by substitution

reaction to give compound 8, then reduced to give aniline 9. Finally, G4451 was obtained from aniline 9 and benzoic acid through acid amine condensation.

(B) The ^{1}H NMR of G4451.
