# Supplementary material for: Integrated screening identifies GPR31 as a key driver and druggable target for metabolic dysfunction–associated steatohepatitis
Source: J Clin Invest. 2025 Sep 2;135(17):e173193. doi: 10.1172/JCI173193 (PMC12404741; doi:10.1172/JCI173193)

**Figure 2**

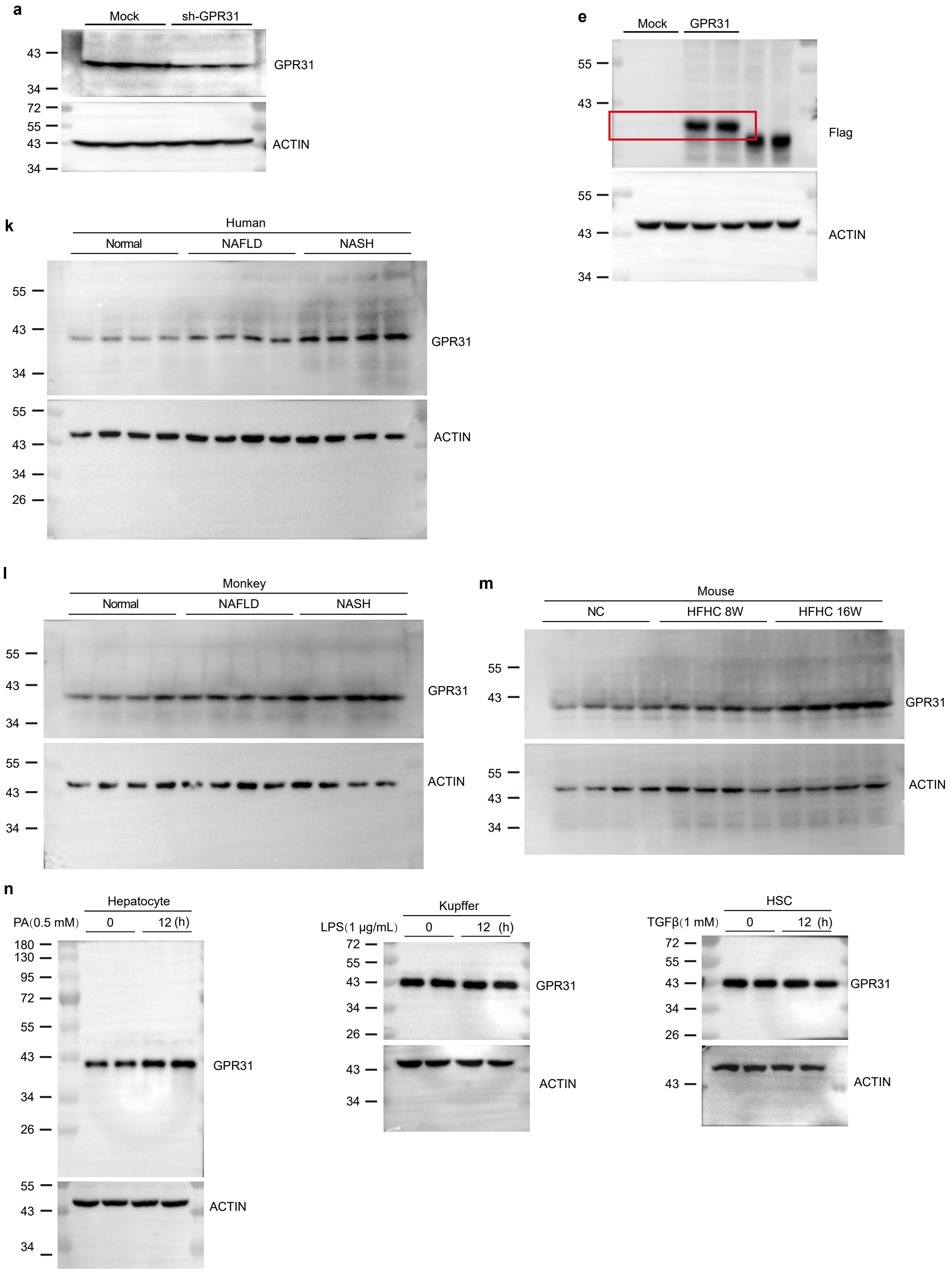
a
Mock
sh-GPR31
GPR31
ACTIN
e
Mock
GPR31
Flag
ACTIN
k
Human
Normal
NAFLD
NASH
GPR31
ACTIN
l
Monkey
Normal
NAFLD
NASH
GPR31
ACTIN
m
Mouse
NC
HFHC 8W
HFHC 16W
GPR31
ACTIN
n
Hepatocyte
PA(0.5 mM)
0
12 (h)
GPR31
ACTIN
Kupffer
LPS(1 μg/mL)
0
12 (h)
GPR31
ACTIN
HSC
TGFβ(1 mM)
0
12 (h)
GPR31
ACTIN
180
130
95
72
55
43
34
26

**Figure 3**

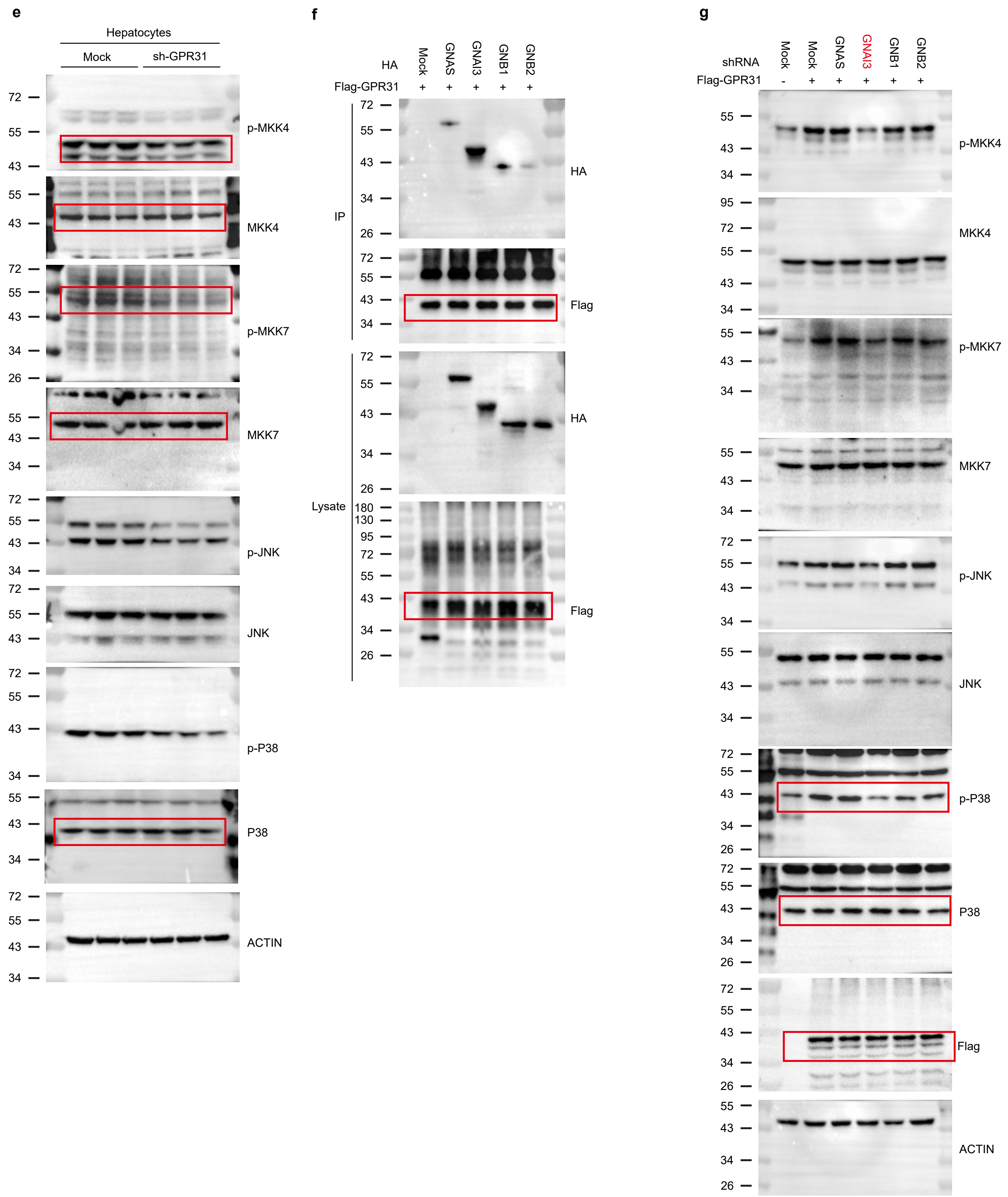

e
Hepatocytes
Mock
sh-GPR31
p-MKK4
MKK4
p-MKK7
MKK7
p-JNK
JNK
p-P38
P38
ACTIN
f
HA
Flag-GPR31
Mock
GNAS
GNAI3
GNB1
GNB2
IP
Lysate
HA
Flag
HA
Flag
g
shRNA
Flag-GPR31
Mock
Mock
GNAS
GNAI3
GNB1
GNB2
p-MKK4
MKK4
p-MKK7
MKK7
p-JNK
JNK
p-P38
P38
Flag
ACTIN

h

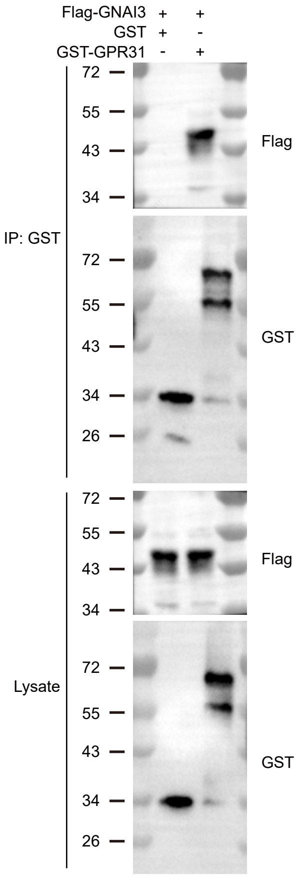
Flag-GNAI3 + +
GST + -
GST-GPR31 - +
IP: GST
Lysate
72
55
43
34
26
Flag
GST

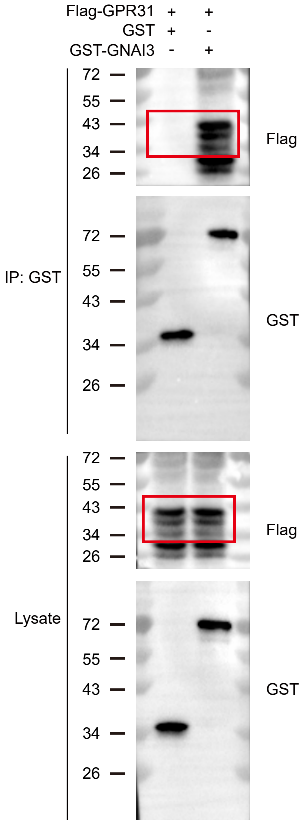
Flag-GPR31 + +
GST + -
GST-GNAI3 - +
IP: GST
Lysate
72
55
43
34
26
Flag
GST

j

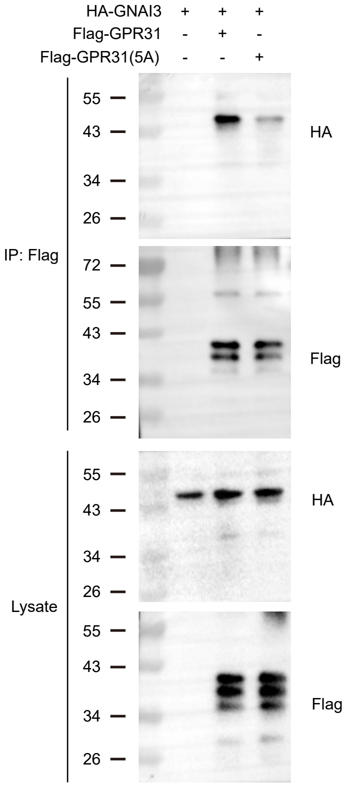
HA-GNAI3 + + +
Flag-GPR31 - + -
Flag-GPR31(5A) - - +
IP: Flag
Lysate
72
55
43
34
26
HA
Flag

Figure 4

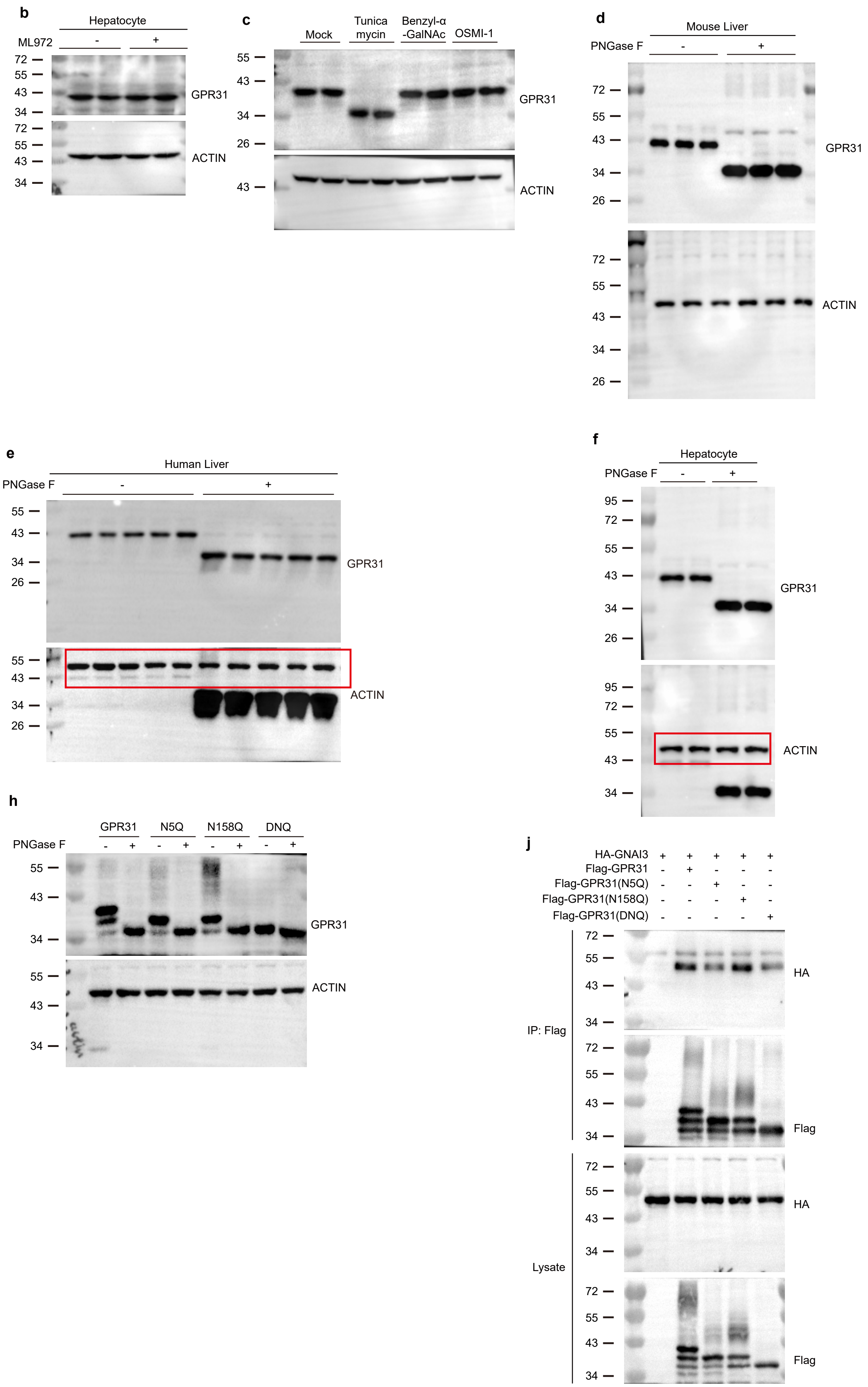
b
Hepatocyte
ML972
-
+
72
55
43
34
GPR31
ACTIN
c
Mock
Tunica mycin
Benzyl-α -GalNAc
OSMI-1
55
43
34
26
GPR31
ACTIN
d
Mouse Liver
PNGase F
GPR31
ACTIN
e
Human Liver
PNGase F
GPR31
ACTIN
f
Hepatocyte
PNGase F
95
GPR31
ACTIN
h
GPR31
N5Q
N158Q
DNQ
PNGase F
GPR31
ACTIN
j
HA-GNAI3
Flag-GPR31
Flag-GPR31(N5Q)
Flag-GPR31(N158Q)
Flag-GPR31(DNQ)
IP: Flag
Lysate
HA
Flag

**Figure 4**

k

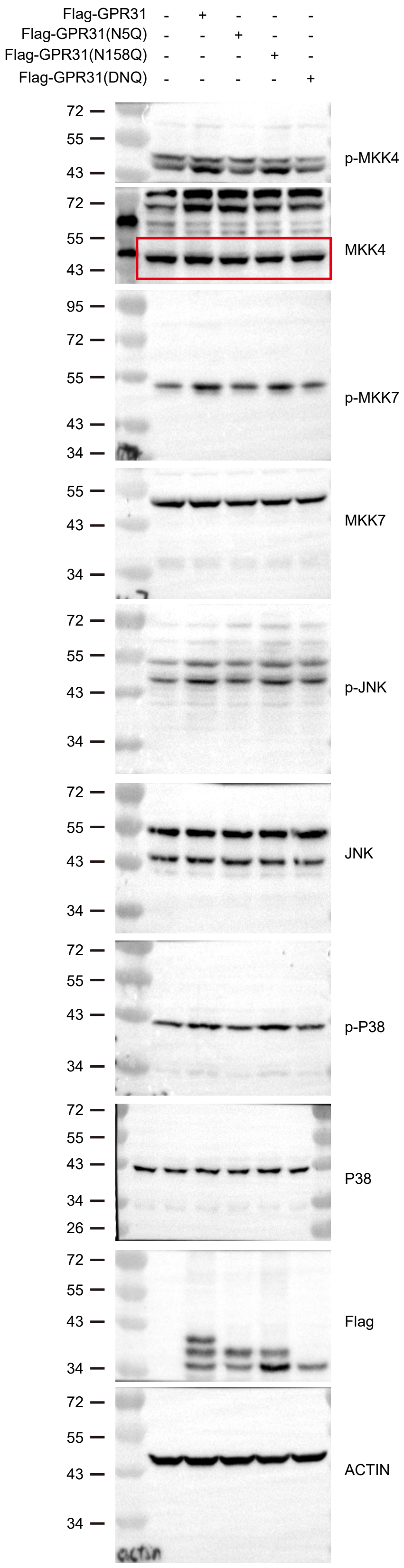


**Figure 5**

a

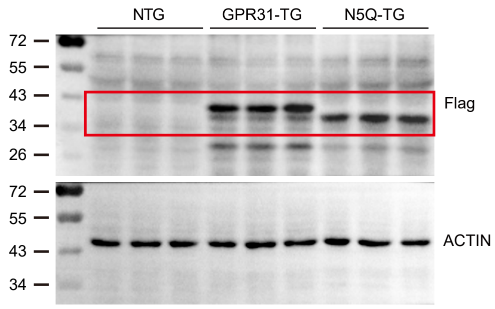


f

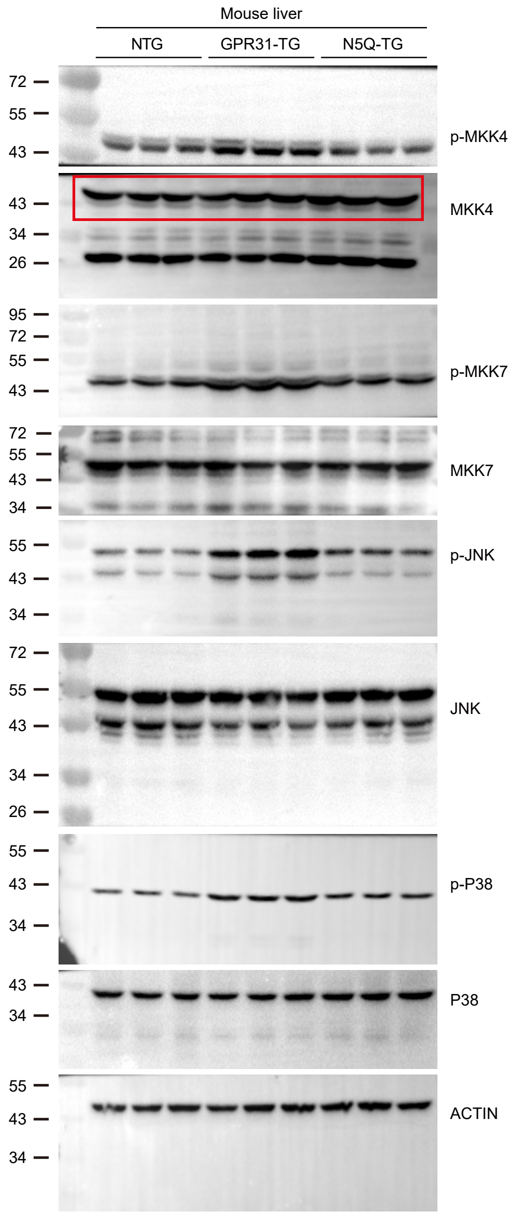

Figure 6

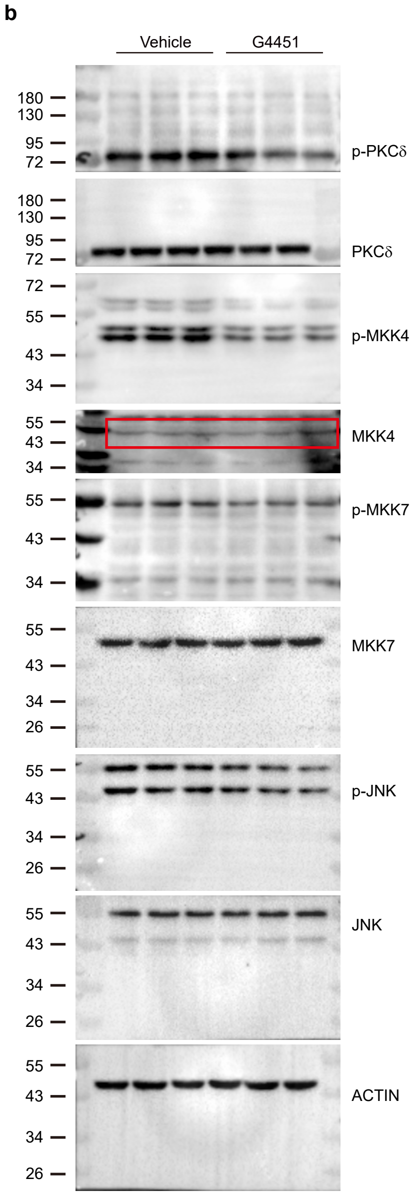


Figure 6

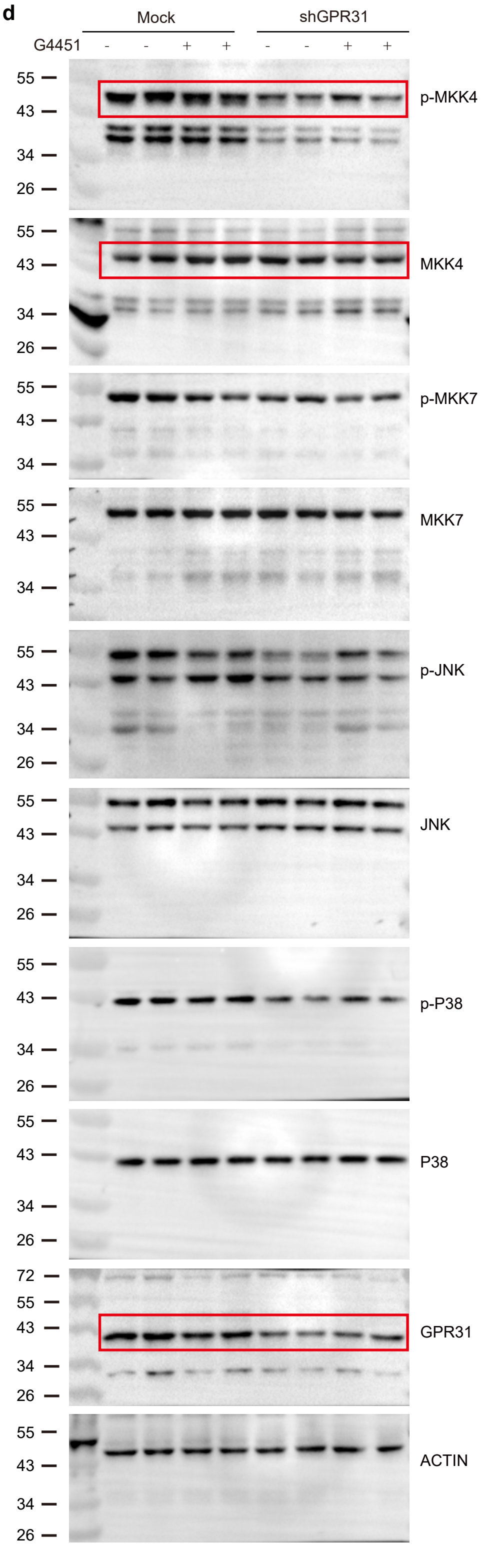

**Figure 6**

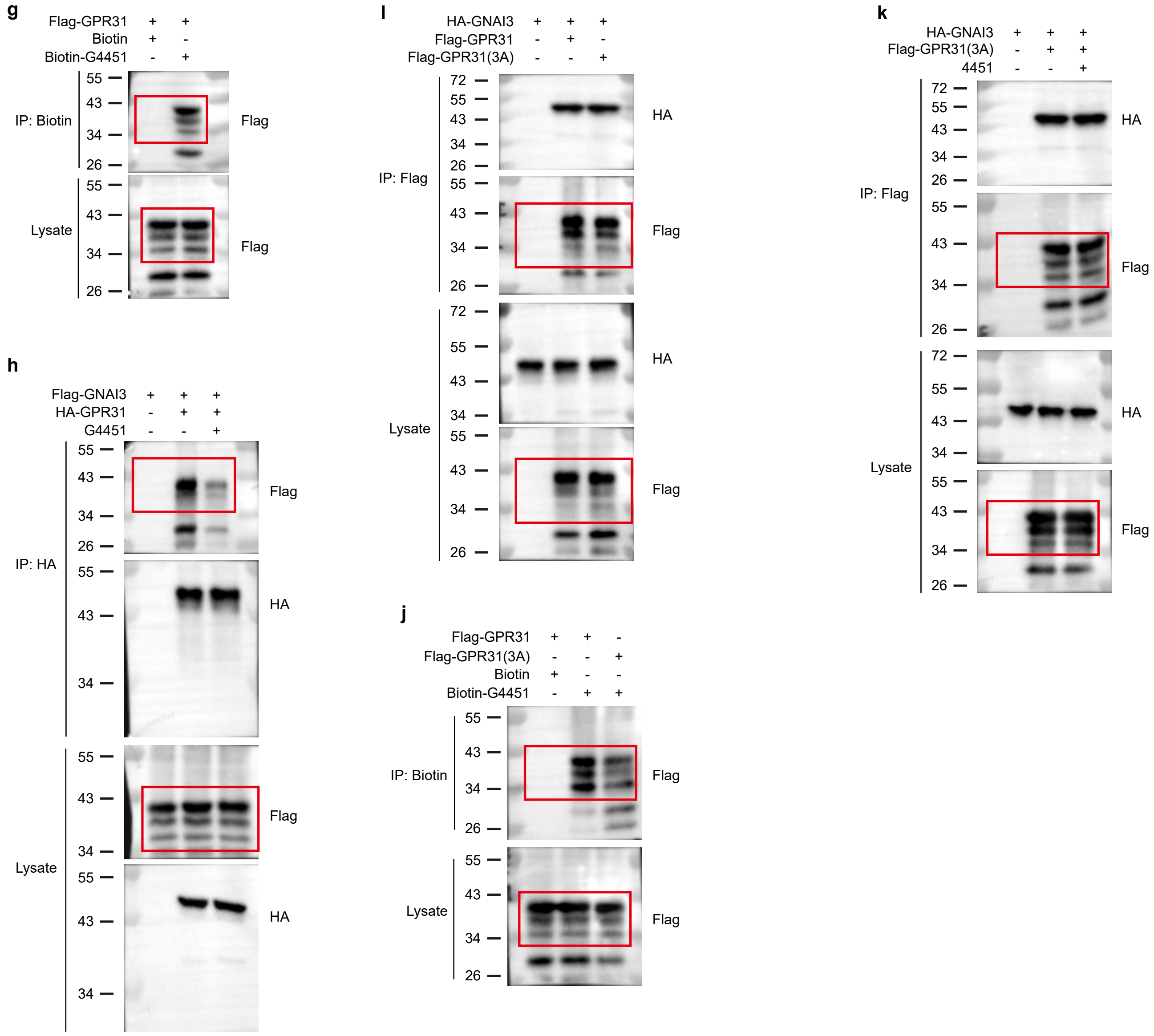
g
Flag-GPR31 + +
Biotin + -
Biotin-G4451 - +
IP: Biotin
Lysate
Flag
55
43
34
26
h
Flag-GNAI3 + + +
HA-GPR31 - + +
G4451 - - +
IP: HA
Lysate
Flag
HA
i
HA-GNAI3 + + +
Flag-GPR31 - + -
Flag-GPR31(3A) - - +
IP: Flag
Lysate
HA
Flag
72
j
Flag-GPR31 + + -
Flag-GPR31(3A) - - +
Biotin + - -
Biotin-G4451 - + +
IP: Biotin
Lysate
Flag
k
HA-GNAI3 + + +
Flag-GPR31(3A) - + +
4451 - - +
IP: Flag
Lysate
HA
Flag

**Figure 7**

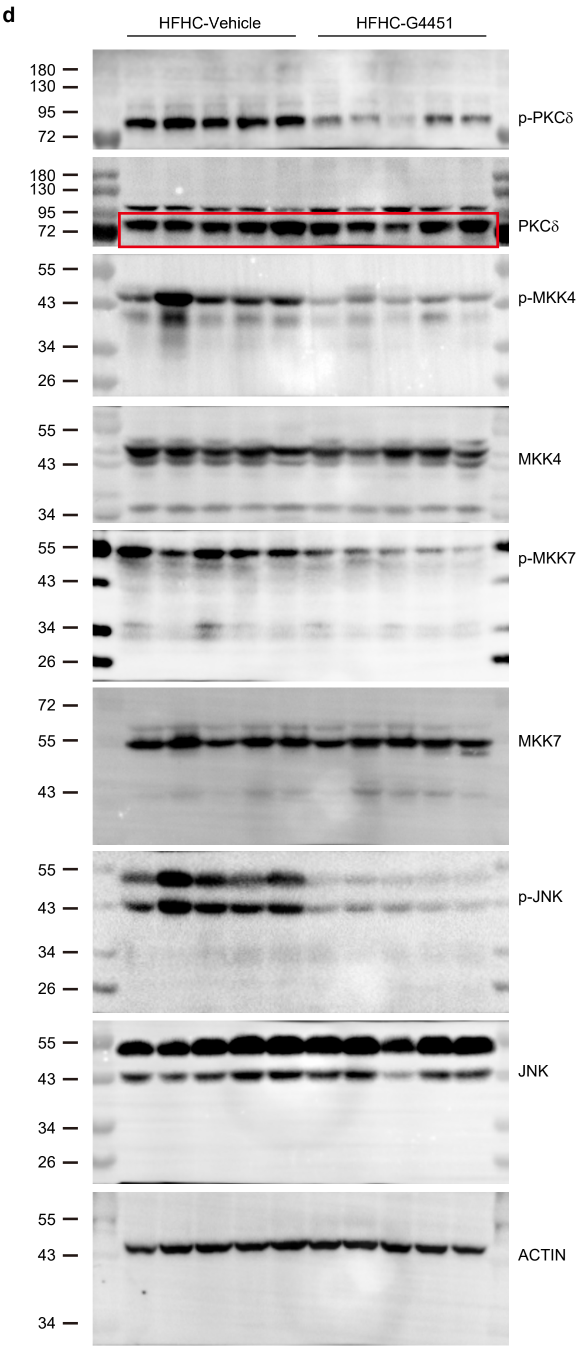


**Figure 8**

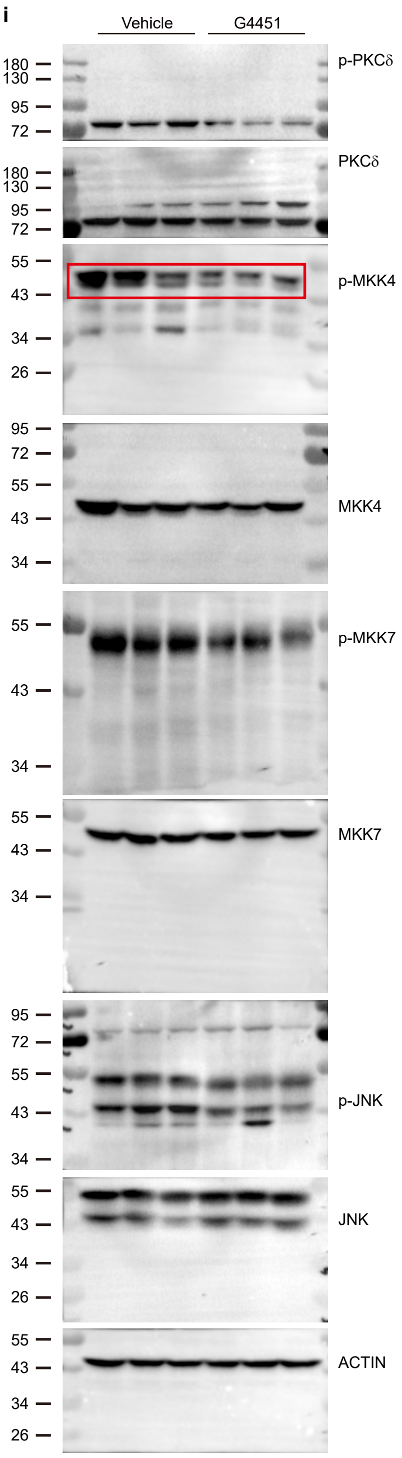

**Figure S2**

c

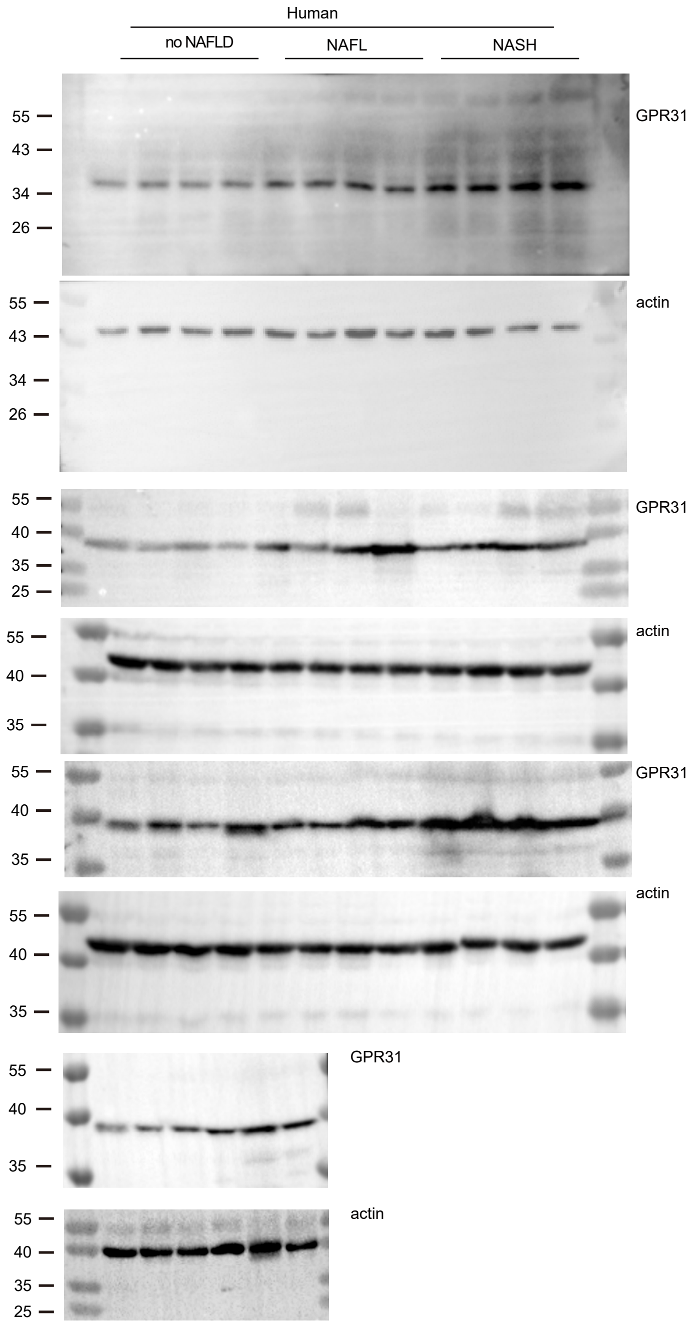
Human
no NAFLD
NAFL
NASH
GPR31
actin
GPR31
actin
GPR31
actin
GPR31
actin
55
43
34
26
40
35
25

e

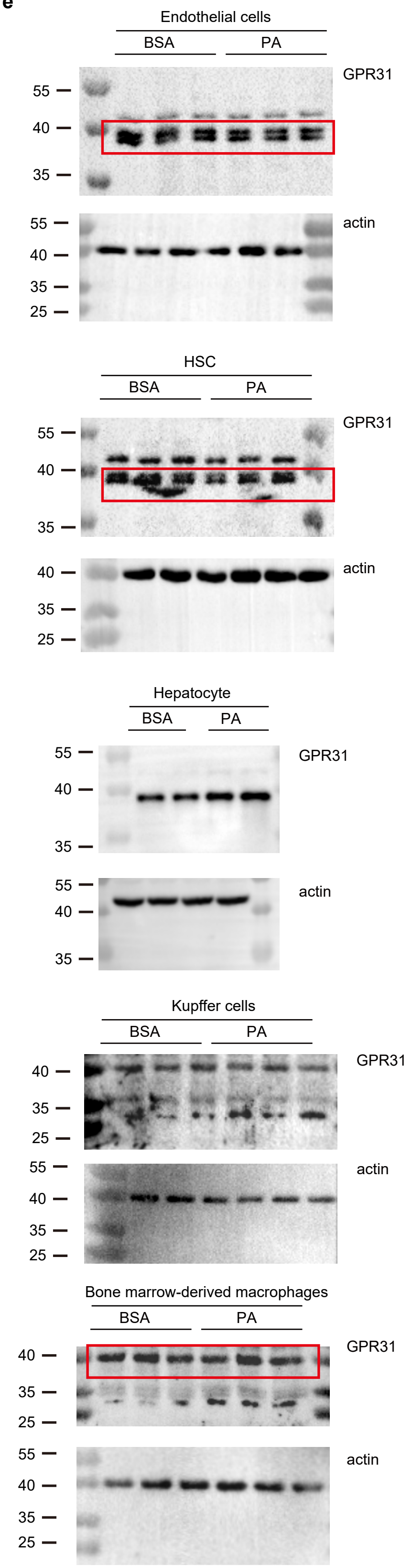
Endothelial cells
BSA
PA
GPR31
actin
HSC
BSA
PA
GPR31
actin
Hepatocyte
BSA
PA
GPR31
actin
Kupffer cells
BSA
PA
GPR31
actin
Bone marrow-derived macrophages
BSA
PA
GPR31
actin
55
40
35
25

**Figure S3**

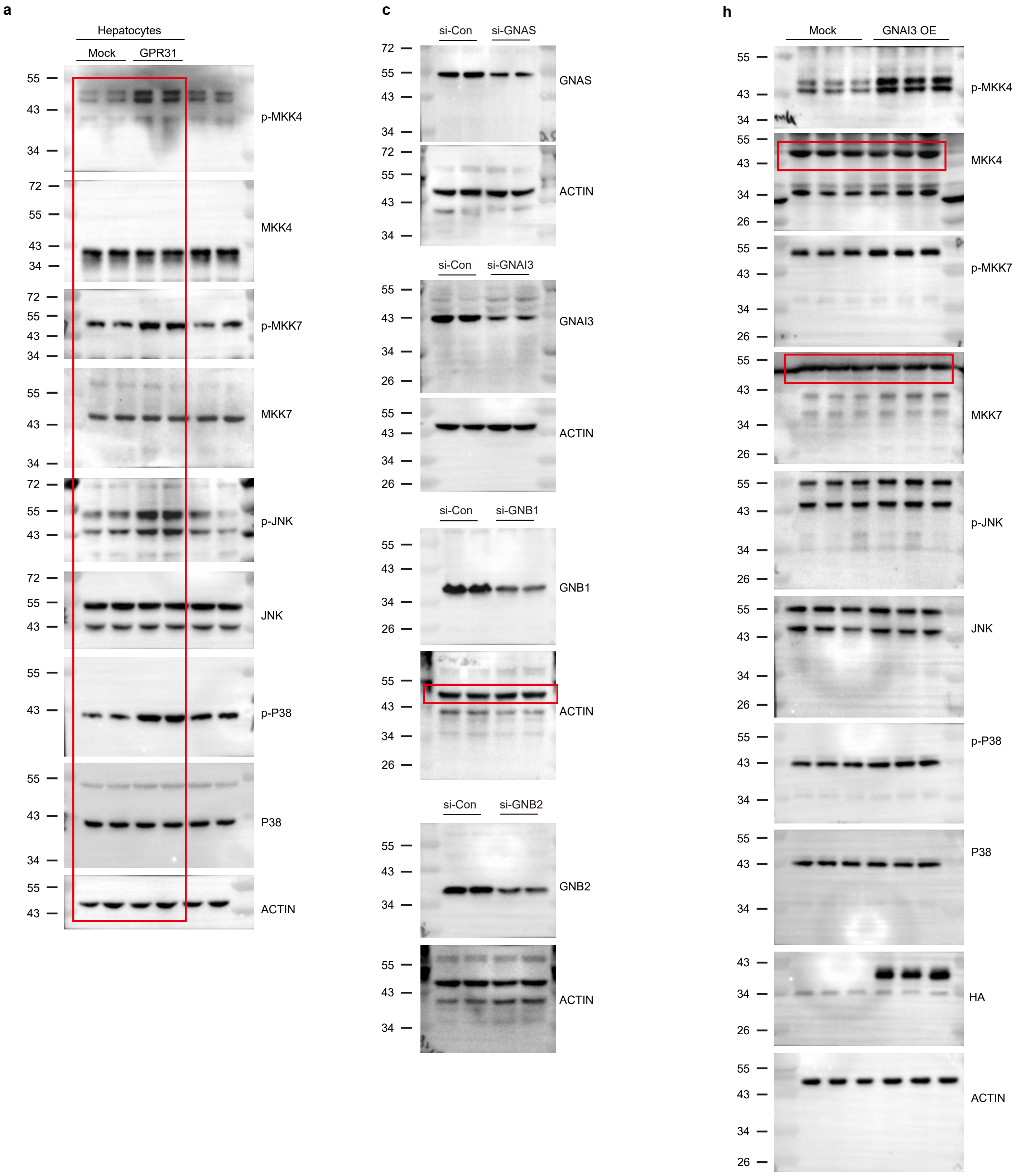
a
Hepatocytes
Mock
GPR31
p-MKK4
MKK4
p-MKK7
MKK7
p-JNK
JNK
p-P38
P38
ACTIN
c
si-Con
si-GNAS
GNAS
ACTIN
si-GNAI3
GNAI3
si-GNB1
GNB1
si-GNB2
GNB2
h
GNAI3 OE
HA
72
55
43
34
26

## Figure S3

k

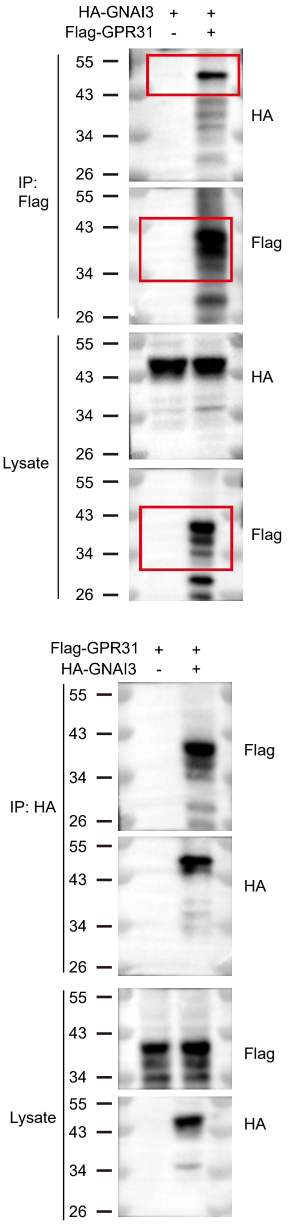
HA-GNAI3 + +
Flag-GPR31 - +
IP: Flag
55
43
34
26
HA
Flag
Lysate
Flag-GPR31 + +
HA-GNAI3 - +
IP: HA

l

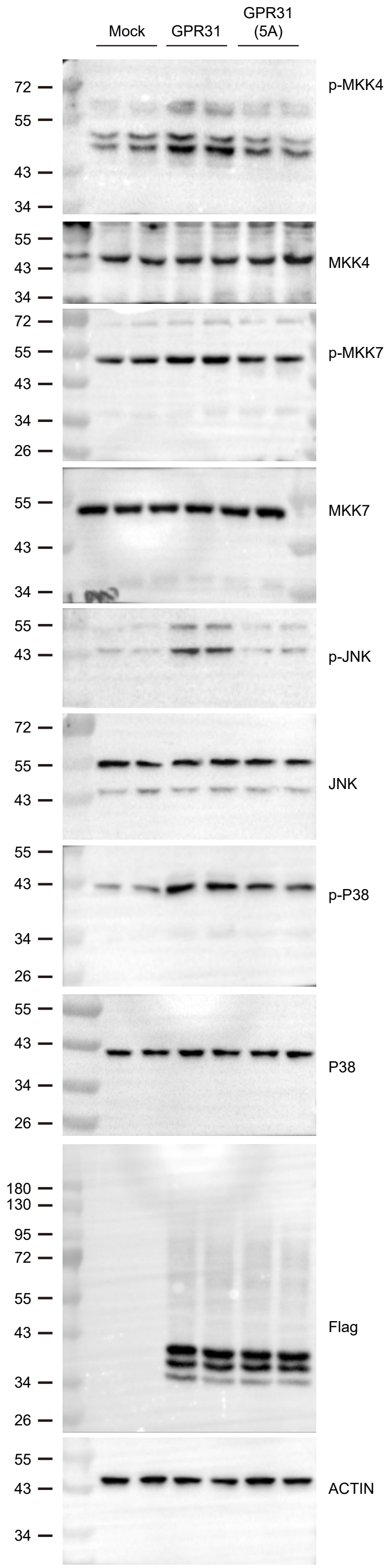
Mock
GPR31
GPR31 (5A)
p-MKK4
MKK4
p-MKK7
MKK7
p-JNK
JNK
p-P38
P38
Flag
ACTIN
180
130
95
72
55
43
34
26

**Figure S4**

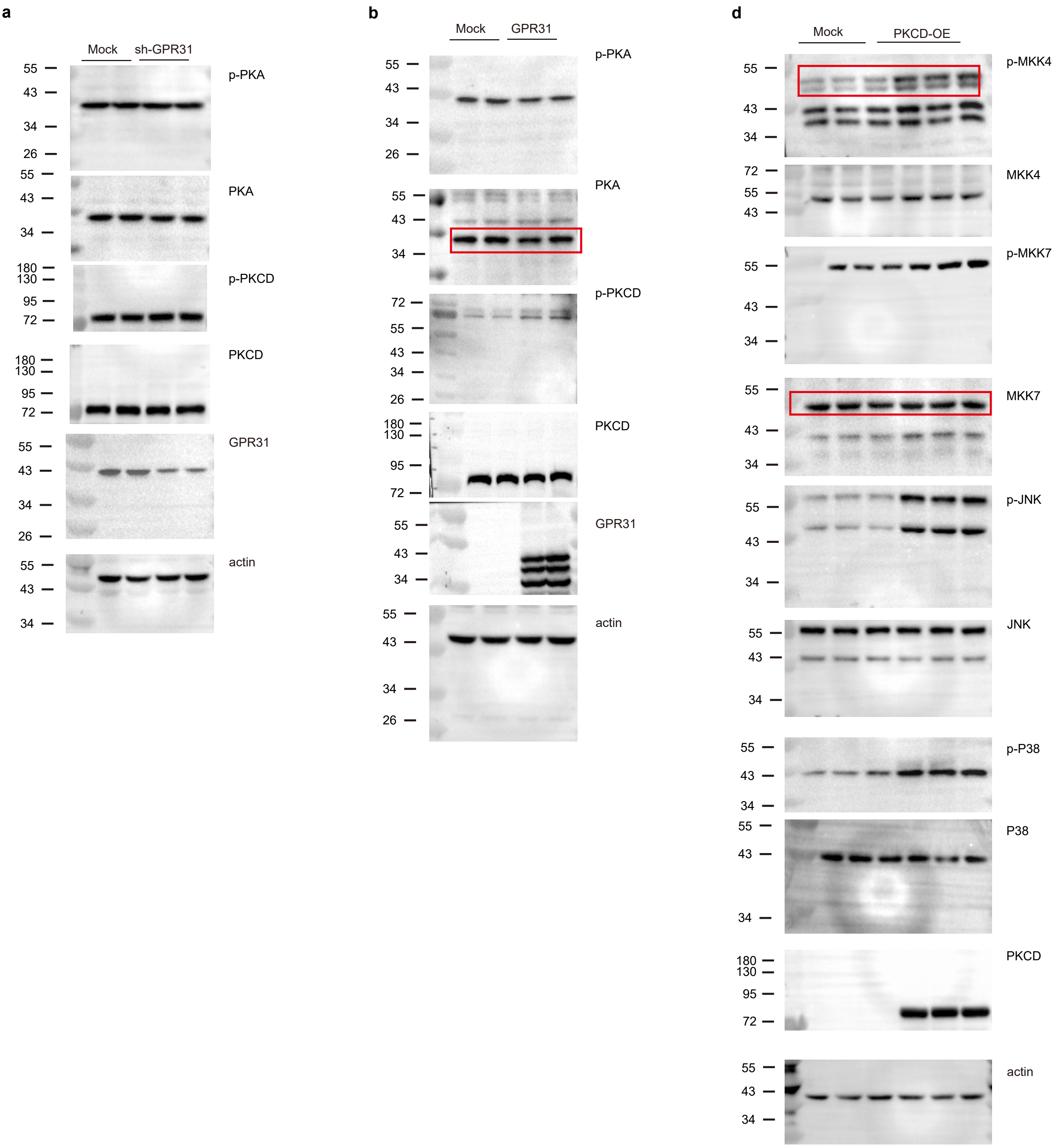
a
Mock
sh-GPR31
p-PKA
PKA
p-PKCD
PKCD
GPR31
actin
b
Mock
GPR31
p-PKA
PKA
p-PKCD
PKCD
GPR31
actin
d
Mock
PKCD-OE
p-MKK4
MKK4
p-MKK7
MKK7
p-JNK
JNK
p-P38
P38
PKCD
actin

# Figure S4

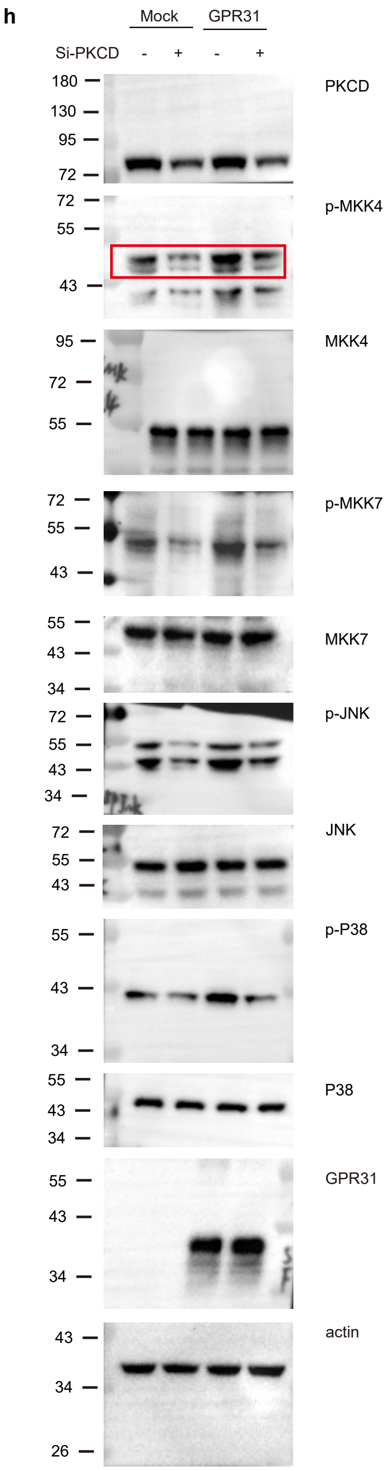


# Figure S5

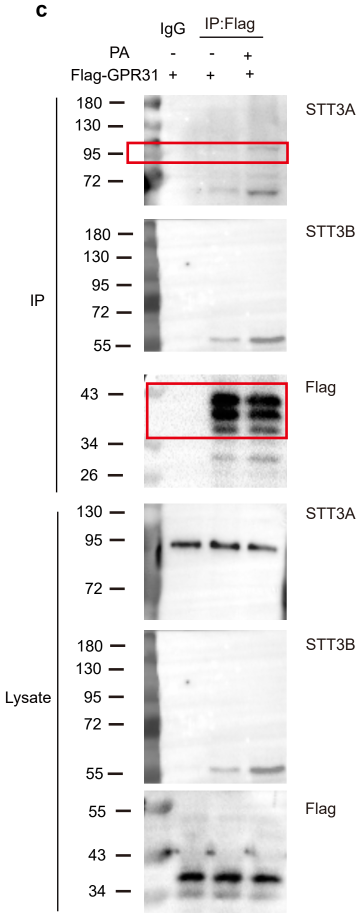


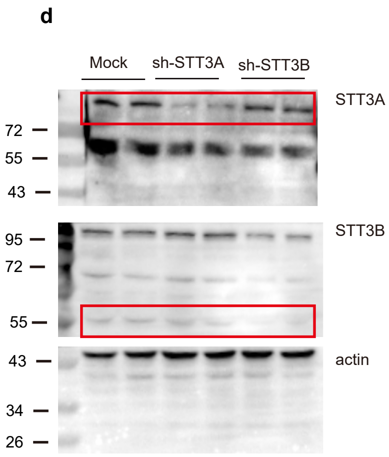


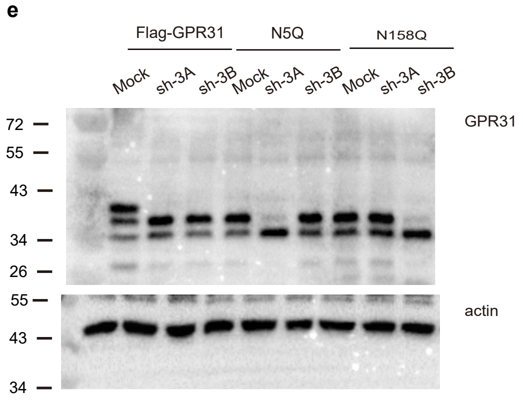

**Figure S6**

c

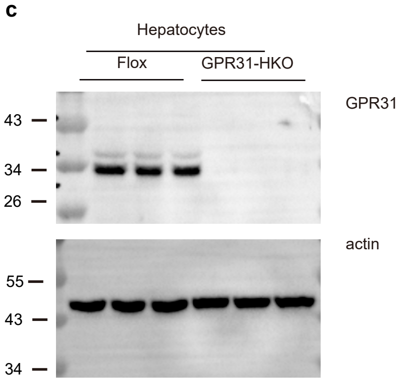
Hepatocytes
Flox
GPR31-HKO
43
34
26
GPR31
55
43
34
actin

i

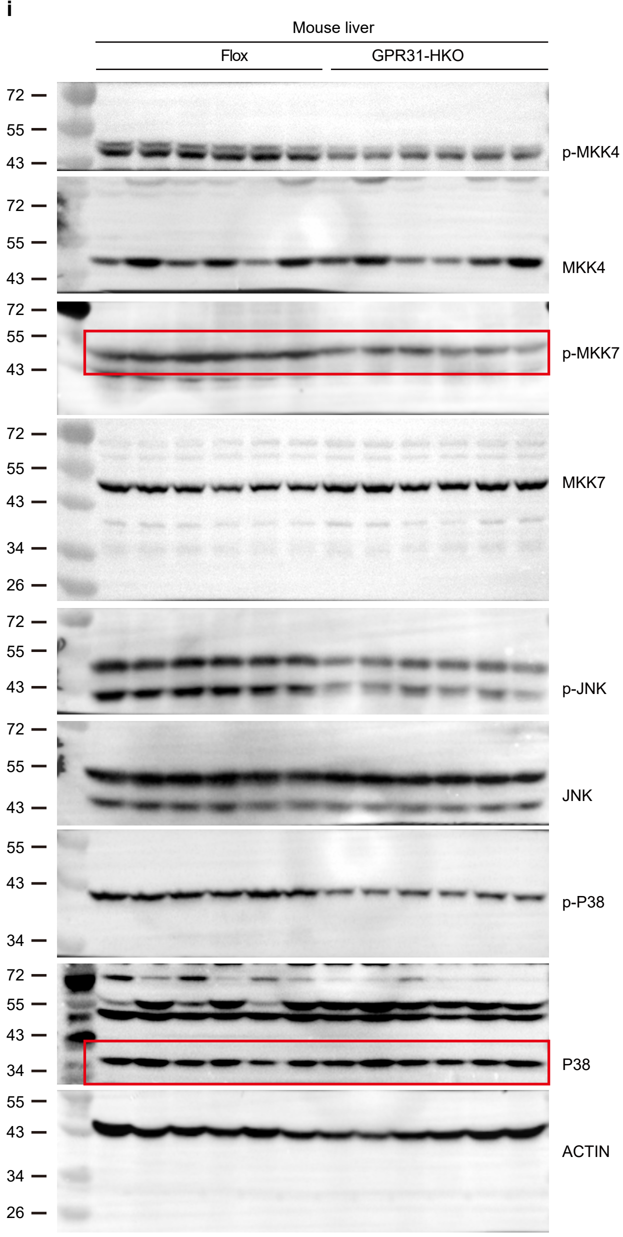
Mouse liver
Flox
GPR31-HKO
72
55
43
p-MKK4
MKK4
p-MKK7
MKK7
34
26
p-JNK
JNK
p-P38
P38
ACTIN

**Figure S7**

**h**

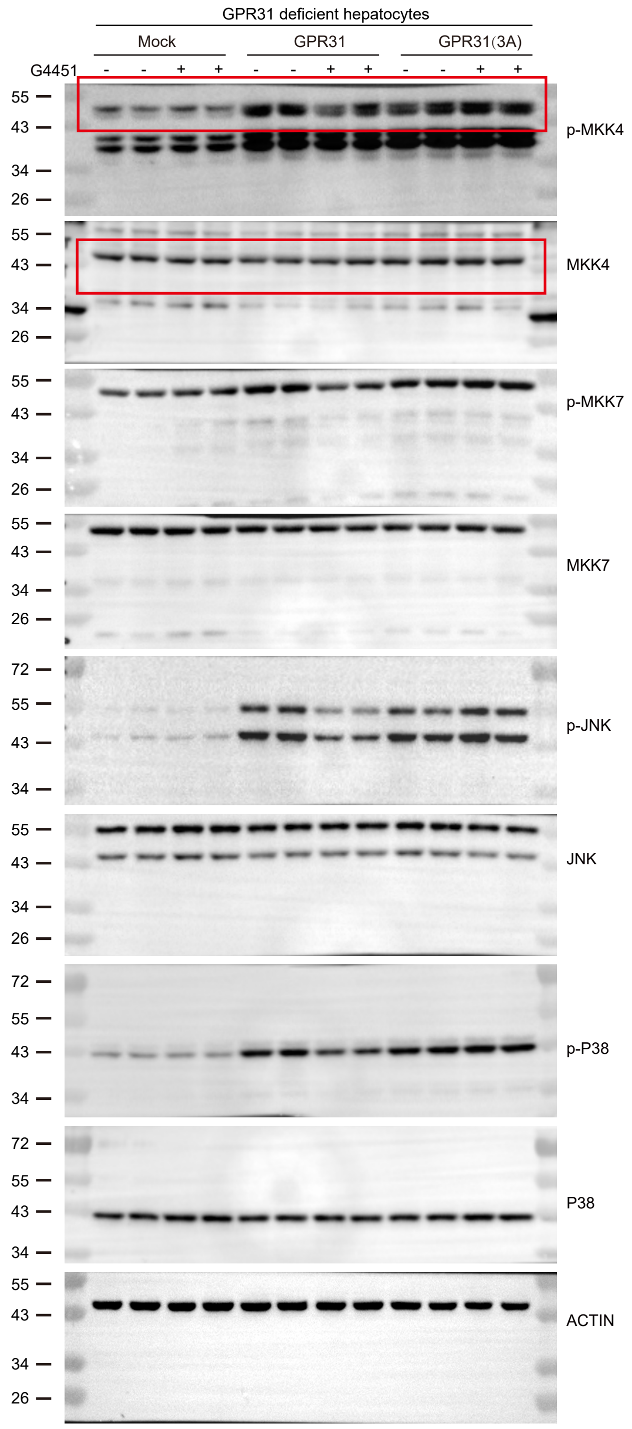


**Figure S8**

**a**

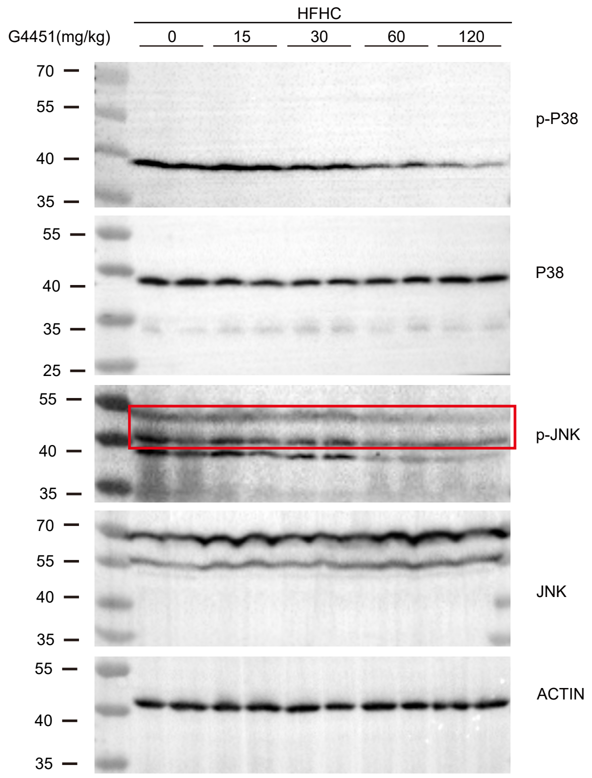

Supplement: Unedited blot and gel images [file jci-135-173193-s056.pdf]
